# Supplementary material for: An atlas of photonic and plasmonic materials for cathodoluminescence microscopy
Source: Nanophotonics. 2025 Jul 3;14(15):2647–71. doi: 10.1515/nanoph-2025-0135 (PMC12322731; doi:10.1515/nanoph-2025-0135)
Supplement: Supplementary file 1 — Supplementary Material Details [file j_nanoph-2025-0135_suppl_001.pdf]

Supplementary information: An atlas of photonic and plasmonic materials for cathodoluminescence microscopy

Sven Ebel,[1] Yonas Lebsir,[1] Torgom Yezekyan,[1] N. Asger Mortensen,[1,2] and Sergii Morozov[1]

[1]*POLIMA—Center for Polariton-driven Light–Matter Interactions, University of Southern Denmark, Campusvej 55, DK-5230 Odense M, Denmark*
[2]*Danish Institute for Advanced Study, University of Southern Denmark, Campusvej 55, DK-5230 Odense M, Denmark*

Contents

Calculating transition radiation spectra 4
Uncorrected cathodoluminescence spectra 5
Angular resolved cathodoluminescence profiles 8
Self-absorption in the cathodoluminescence from TMD bulk crystals 11
Raman spectroscopy 12
Materials 12
- Gold (Au) 13
- Silver (Ag) 14
- Copper (Cu) 15
- Aluminum (Al) 16
- Titanium (Ti) 17
- Platinum (Pt) 18
- Nickel (Ni) 19
- Chromium (Cr) 20
- Silicon (Si) 21
- Germanium (Ge) 22
- Bi_2Te_3 23
- Sb_2S_3 24
- 2H-MoS_2 25
- 3R-MoS_2 26
- 2H-$MoSe_2$ 27
- 2H-WS_2 28
- 2H-WSe_2 29
- 1T'-ReS_2 30

2H-$MoTe_2$ 31
Td-WTe_2 32
1T-TiS_2 33
2H-$NbSe_2$ 34
2H-$TaSe_2$ 35
cBN 36
hBN (micro powder) 37
hBN (Crystal 2D Semiconductors) 38
hBN (Crystal HQ Graphene) 39
Diamond (micro crystals) 40
Diamond (nano crystals with NV centers) 41
Diamond (nano crystals with SiV centers) 42
Graphite 43
Quartz (SiO_2) 44
100 nm SiO_2 on silicon 45
Menzel coverslip 46
BK7 47
Mica 48
α-Si_3N_4 (nano powder) 49
Si_3N_4 (20 nm membrane) 50
β-Si_3N_4 51
4H-SiC 52
6H-SiC 53
TiO_2 54
ITO 55
ZrO_2 56
Ta_2O_5 57
MoO_3 58
$LiNbO_3$ 59
Al_2O_3 60
Yb_2O_3 61
GaN 62
GaP 63
MgF_2 64

CaF_2 65
BaF_2 66
AlN 67
PDMS 68
PC 69
C-tape 70
HSQ 71
PMMA 72
SU-8 73

Calculating transition radiation spectra

The transition radiation (TR) calculations presented here follow the theoretical framework of Sec. IV in Ref. [1], assuming a swift electron traveling along the *z*-axis at constant velocity *v* and crossing the vacuum–metal interface, where the metal extends in the region *z* < 0. We assign the vacuum medium *j* the index 1, and the metal medium the index 2. The electric field generated by the moving electron in every medium *j* can be separated into the contribution of the external electron field for an infinite bulk material plus the field reflected at the surface:

$$\boldsymbol{E} = \boldsymbol{E}_j^{ext} + \boldsymbol{E}_j^{ref}$$

The reflected component of the field originates from induced surface charges and currents. The bulk field $\boldsymbol{E}_j^{ext}$ decays evanescently from the electron's trajectory.

Using Faraday`s law the corresponding magnetic fields can be derived with:

$$\boldsymbol{H}_j^{ext}(\boldsymbol{q}_{\parallel}, z, \omega) = sgn(v)\frac{4\pi i e q_{\parallel}}{c} e^{i\omega z/v} \frac{\hat{\varphi}_{\boldsymbol{q}}}{q^2 - k^2\epsilon_1}$$

$$\boldsymbol{H}_j^{ref}(\boldsymbol{q}_{\parallel}, z, \omega) = \frac{-2\pi}{q_{zj}}\begin{cases} e^{iq_{z1}z}(\boldsymbol{q}_{\parallel}, q_{z1}) \times \boldsymbol{h}_1, & j = 1, \\ e^{-iq_{z2}z}(\boldsymbol{q}_{\parallel}, -q_{z2}) \times \boldsymbol{h}_2, & j = 2, \end{cases}$$

where $\boldsymbol{q}_{\parallel}$and $\hat{\varphi}_{\boldsymbol{q}} = \hat{z} \times \boldsymbol{q}_{\parallel}$ form the set $\{\boldsymbol{q}_{\parallel}, \hat{\varphi}_{\boldsymbol{q}}, \hat{z}\}$ building a three-dimensional reference frame. Further we introduce $q_{zj} = \sqrt{k^2\epsilon_j - q_{\parallel}{}^2}$, $q = \sqrt{\boldsymbol{q}_{\parallel}{}^2 + (\omega/v)^2}$ and $\boldsymbol{h}_j$ which denotes the generated surface currents. k and ω are the absolute of the wavevector and the angular frequency of the generated field, respectively. As the homogenous field $\boldsymbol{H}_j^{ext}$ decays evanescently away from the electron trajectory, the TR emission must arise from $\boldsymbol{H}^{ref}$which can be expressed for the far field of medium 1.

$$\boldsymbol{H}^{ref}(r, \omega) \xrightarrow{kr \to \infty} f_H(\theta, \omega)\frac{e^{ikr}}{r}$$

The far field amplitude is given by:

$$f_H(\theta, \omega) = ik\cos\theta \frac{2ieq_{\parallel}/c}{q_{z1}\epsilon_2 + q_{z2}\epsilon_1}\frac{1}{|v|}\left(\frac{\omega\epsilon_2 + vq_{z2}\epsilon_1}{q^2 - k^2\epsilon_1} - \frac{\omega\epsilon_1 + vq_{z2}\epsilon_1}{q^2 - k^2\epsilon_2}\right)\hat{\varphi}$$

In this expression ϵ_1 and ϵ_2 denote the complex permittivity of the media forming he interface. This expression can now be evaluated at $\boldsymbol{q}_{\parallel} = \mathrm{k}\sin\theta$, with θ being the emission angle with respect to the surface normal, and by performing the angular integral we obtain the TR probability:

$$\Gamma_{\mathrm{TR}}(\omega) = \frac{1}{2\pi\hbar k}\int_0^{\pi/2} \sin\theta\, |f_H(\theta, \omega)|^2 d\theta$$

As the far field amplitude is a function of electron velocity, the electron energy affects the TR amplitude. This is because the electron field extends further from its trajectories for higher energies and thereby inducing more surface currents and increasing the TR emission. Am increase in beam current will affect the TR probability by scaling the TR response linearly, as $\Gamma_{\mathrm{TR}}(\omega)$ is given as the TR probability per electron. This has the consequence that an increase in beam current will only affect the TR amplitude constantly over all wavelengths. We evaluate this expression for 30 keV electrons and several metals (Au, Pt, Ti) forming the vacuum-metal interface.

[1] F. J. García de Abajo, “Optical excitations in electron microscopy,” Reviews of Modern Physics, vol. 82, no. 1. American Physical Society (APS), pp. 209–275, Feb. 03, 2010. doi: 10.1103/revmodphys.82.209.

Uncorrected cathodoluminescence spectra

In this section we provide the recorded cathodoluminescence (CL) spectra before correction for system response function.

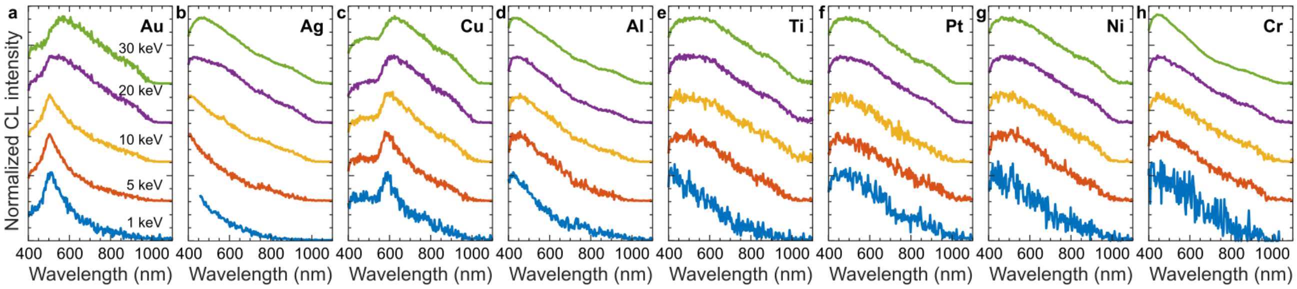

Fig. S1 CL of elemental metals. Normalized uncorrected CL spectra measured in a range of electron beam energies (see panel **h** for color coding, the spectra are vertically offset for clarity of presentation) for: **a** mono-crystalline Au, **b** Ag, **c** Cu, **d** Al, **e** Ti, **f** Pt, **g** Ni, **h** Cr.

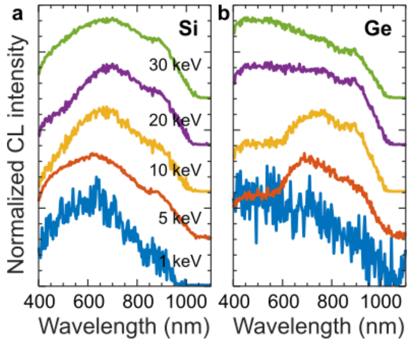

Fig. S2 CL of elemental metalloids. Normalized uncorrected CL spectra measured in a range of electron beam energies for: **a** Si, and **b** Ge.

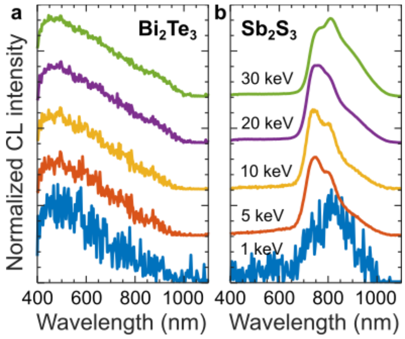

Fig. S3 CL of topological insulators. Normalized uncorrected CL spectra measured in a range of electron beam energies for: **a** Bi_2Te_3, and **b** Sb_2S_3

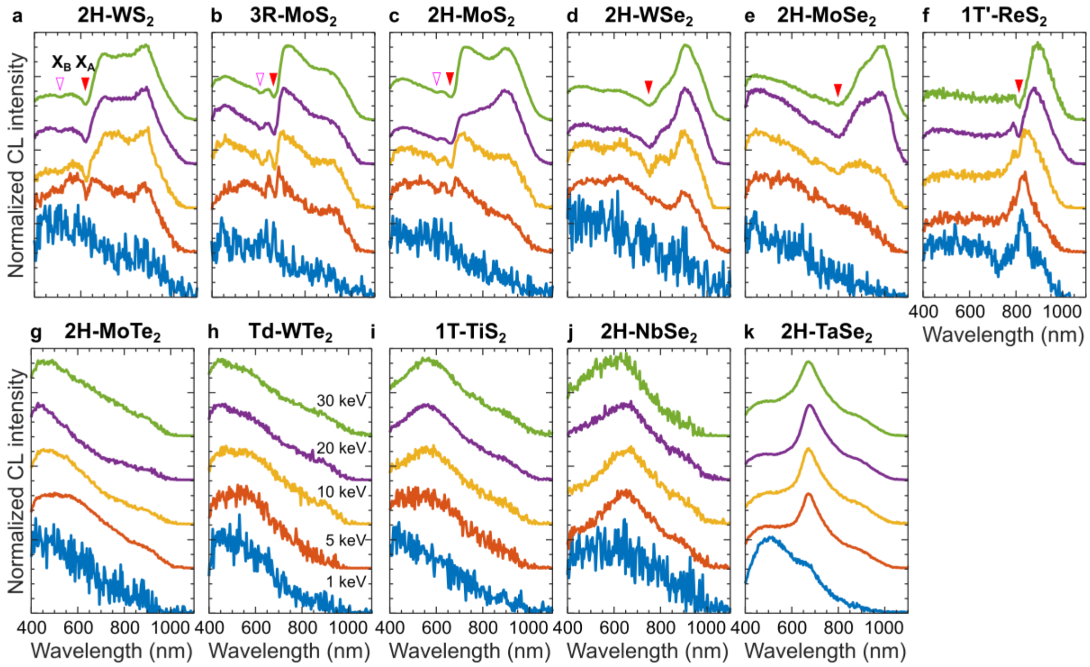

Fig. S4 CL of TMD crystals. Normalized uncorrected CL spectra measured in a range of electron beam energies for a variety of TMD crystals: **a** 2H-WS_2, **b** 3R-MoS_2, **c** 2H-MoS_2, **d** 2H-WSe_2, **e** 2H-$MoSe_2$, **f** 1T'-ReS_2, **g** 2H-$MoTe_2$, **h** Td-WTe_2, **i** 1T-TiS2, **j** 2H-$NbSe_2$, and **k** 2H-$TaSe_2$. Spectral dips corresponding to excitons X_A (red triangle) and X_B (open magenta triangle) are indicated for relevant TMD crystals.

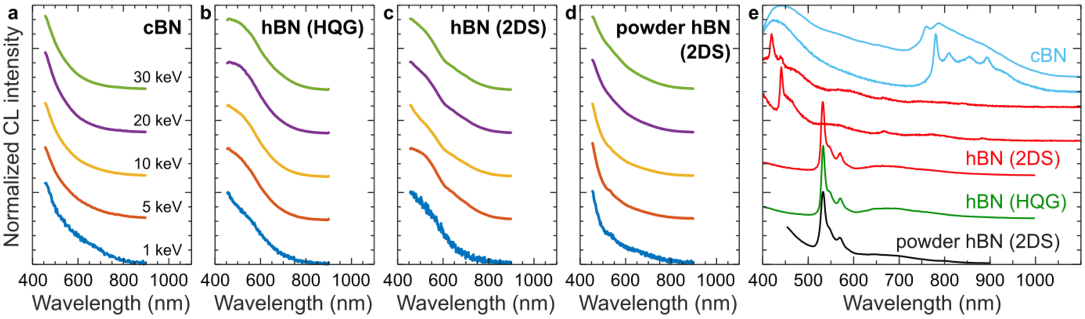

Fig. S5 CL of cBN and hBN and BN-based narrow-line emitters. Normalized uncorrected CL spectra measured in a range of electron beam energies for: **a** micro-crystals of cBN; **b** hBN crystal from HQ Graphene (HQG); **c** hBN crystal from 2D Semiconductors (2DS); **d** low quality hBN in form of micro-power powder from 2DS. **e** Normalized CL spectra of various defects and color centers measured from cBN (blue), hBN from 2DS (red), hBN powder from 2DS (red), and hBN from HQG (green).

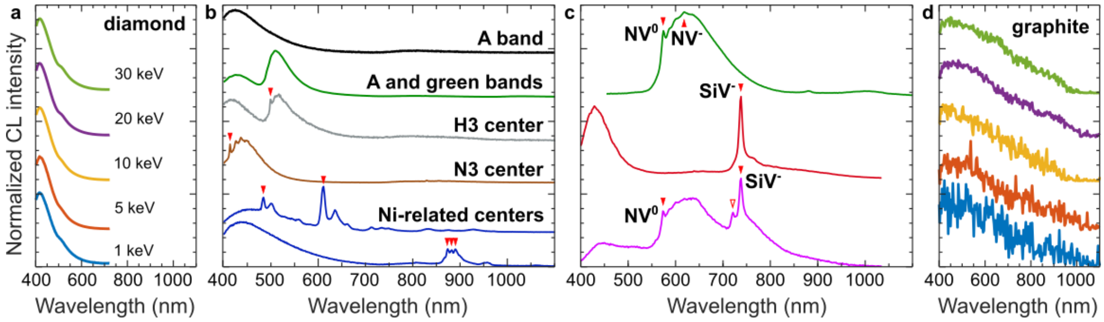

Fig. S6 CL of diamonds and graphite. Normalized uncorrected CL spectra measured in a range of electron beam energies for: **a** diamond microcrystal (ThermoFisher) showing pure A-band emission, measured at various electron beam energies. **b** Defect emission in microcrystals (40-60 µm, ThermoFisher). The spectral positions of the emission lines discussed in the main text are marked with red triangles. **c** Color centers in doped nanodiamonds (<1 µm, Adamas Technologies). **d** Normalized CL spectra of graphite, measured at various electron beam energies (see panel **a** for color coding).

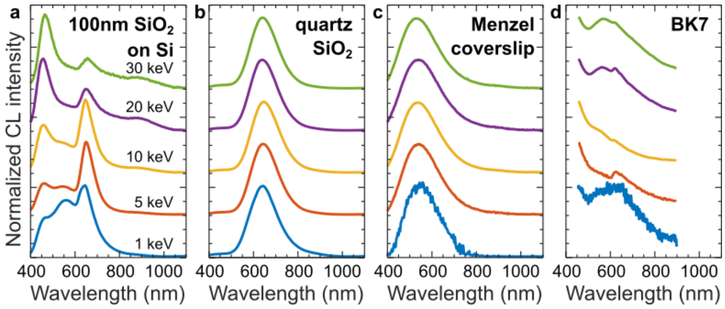

Fig. S7 CL of common glasses. Normalized uncorrected CL spectra measured in a range of electron beam energies for: **a** 100 nm-thick layer of SiO_2 on bulk Si, **b** α-quartz (SiO_2), **c** Menzel glass, and **d** BK7 glass.

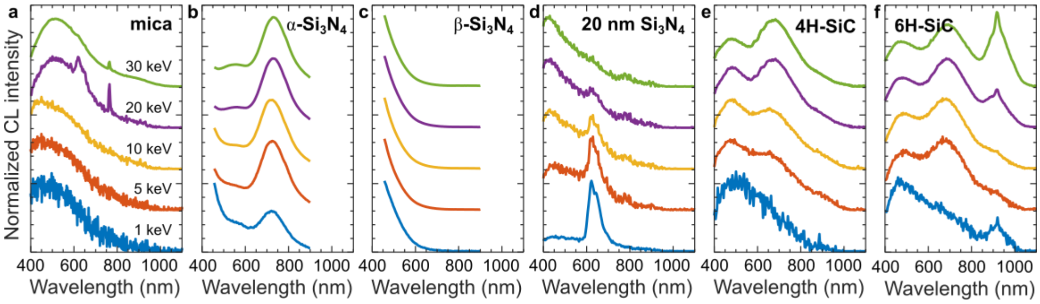

Fig. S8 CL of Si-based materials. Normalized uncorrected CL spectra measured in a range of electron beam energies for: **a** mica, **b** α-Si_3N_4 powder, **c** crystalline β-Si_3N_4, **d** free-standing 20 nm thick Si_3N_4 membrane, **e** 4H-SiC, and **f** 6H-SiC.

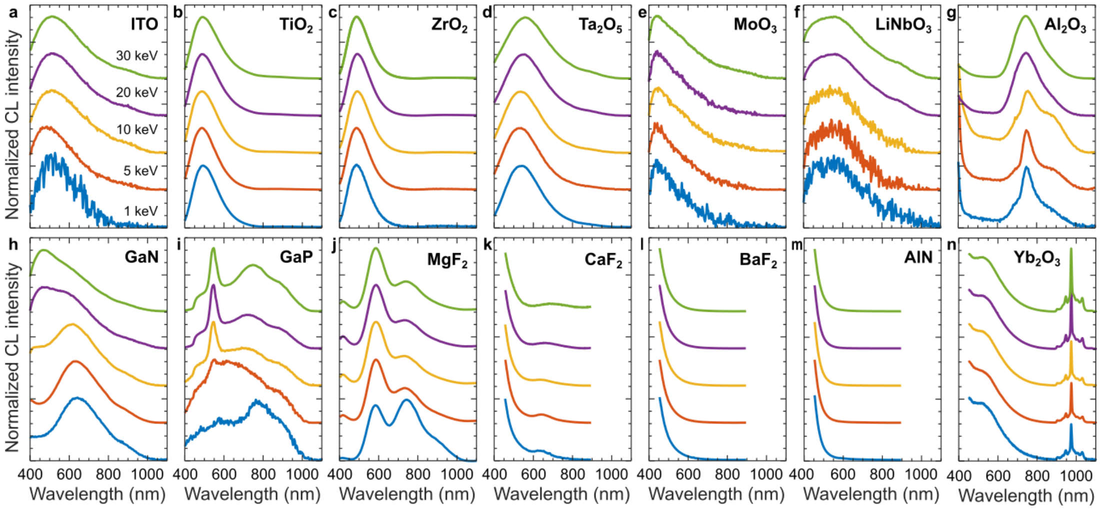

Fig. S9 CL of common photonic materials and optical coatings. Normalized uncorrected CL spectra measured in a range of electron beam energies for: **a** ITO, **b** TiO_2, **c** ZrO_2, **d** Ta_2O_5, **e** MoO_3, **f** $LiNbO_3$, **g** α-Al_2O_3, **h** Yb_2O_3, **i** GaN, **j** GaP, **k** MgF2, **l** CaF2, **m** BaF_2, and **n** AlN.

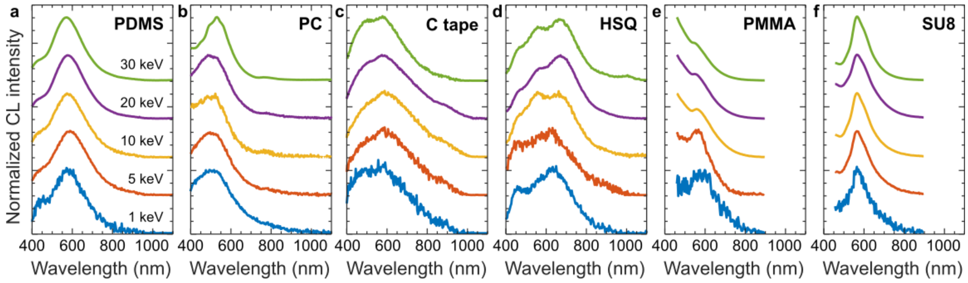

Fig. S10 CL of polymeric and resist materials. Normalized uncorrected CL spectra measured in a range of electron beam energies for: **a** PDMS, **b** PC, **c** sticky carbon tape, **d** HSQ, **e** PMMA, and **f** SU8.

Angular resolved cathodoluminescence profiles

In this section we provide the angular resolved CL profiles of all the materials, which are not presented in the main text.

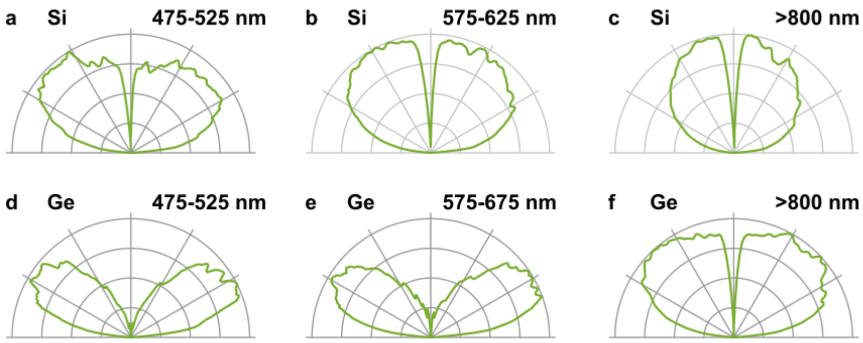

Fig. S11 Spectrally filtered angular emission profiles of Si and Ge. Transition from dipolar to isotropic emission pattern at longer wavelength for Si (**a-c**) and Ge (**d-f**) measured at 30 keV. We use bandpass and longpass filters as indicated in panels in angular spectra measurements.

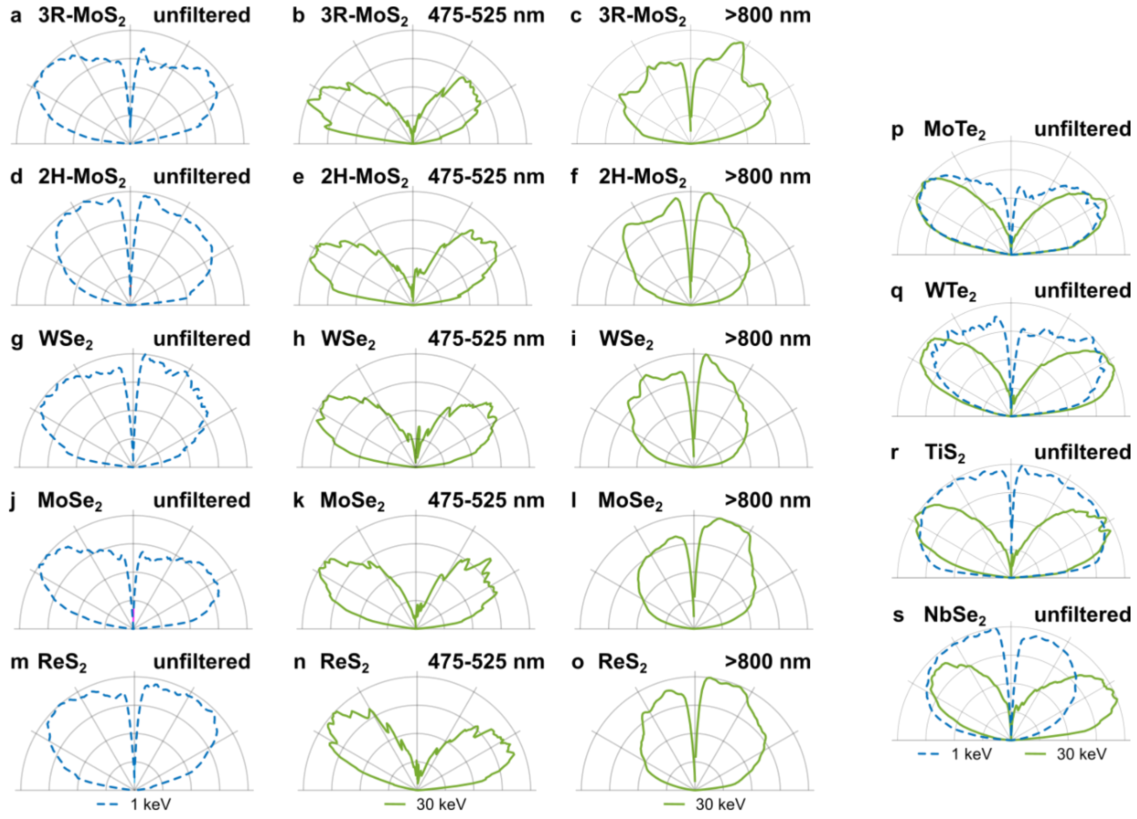

Fig. S12 Angular emission profiles of bulk TMD crystals. The blue dashed profiles correspond to 1 keV measurement, and the solid greed profiles – to 30 keV measurements. The spectral filtering windows are indicated in each panel. **a-c** 3R-MoS_2, **d-f** 2H-MoS_2, **g-l** 2H-WSe_2, **j-l** 2H-$MoSe_2$, **m-o** 1T'-ReS_2, **p** 2H-$MoTe_2$, **1** Td-WTe_2, **r** 1T-TiS2, **s** 2H-$NbSe_2$.

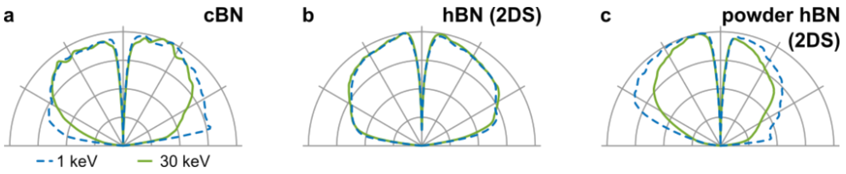

Fig. S13 Angular emission profiles of cBN and hBN crystals. a cBN, **b** hBN from 2D Semiconductors (2DS), **c** hBN powder from 2DS.

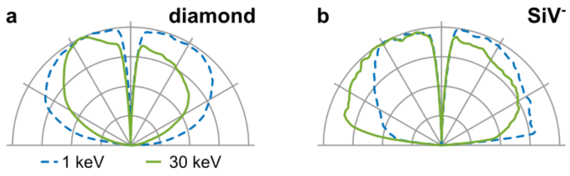

Fig. S14 Angular emission profiles of diamond and SiV$^-$ color center. a diamond, **b SiV$^-$** emission was filtered in 730-750 nm range.

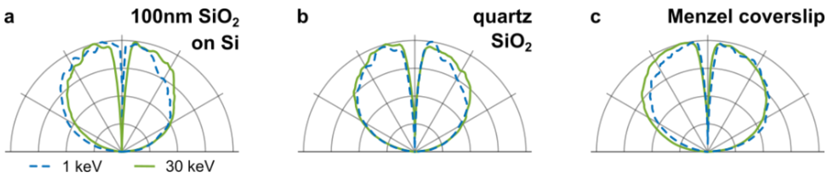

Fig. S15 Angular emission profiles of glasses. a 100 nm-thick layer of SiO_2 on bulk Si, **b** α-quartz, and **c** Menzel coverslip.

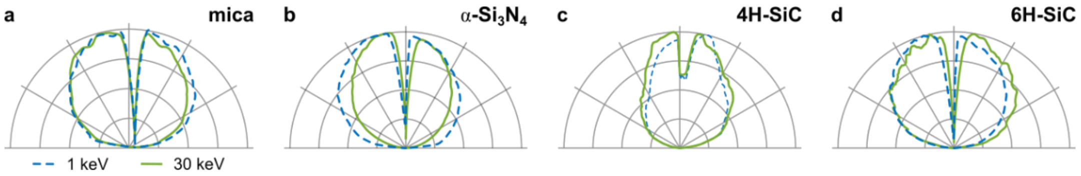

Fig. S16 Angular emission profiles of Si-based materials. **a** mica, **b** α-Si_3N_4 nano powder, **c** 4H-SiC, and **d** 6H-SiC.

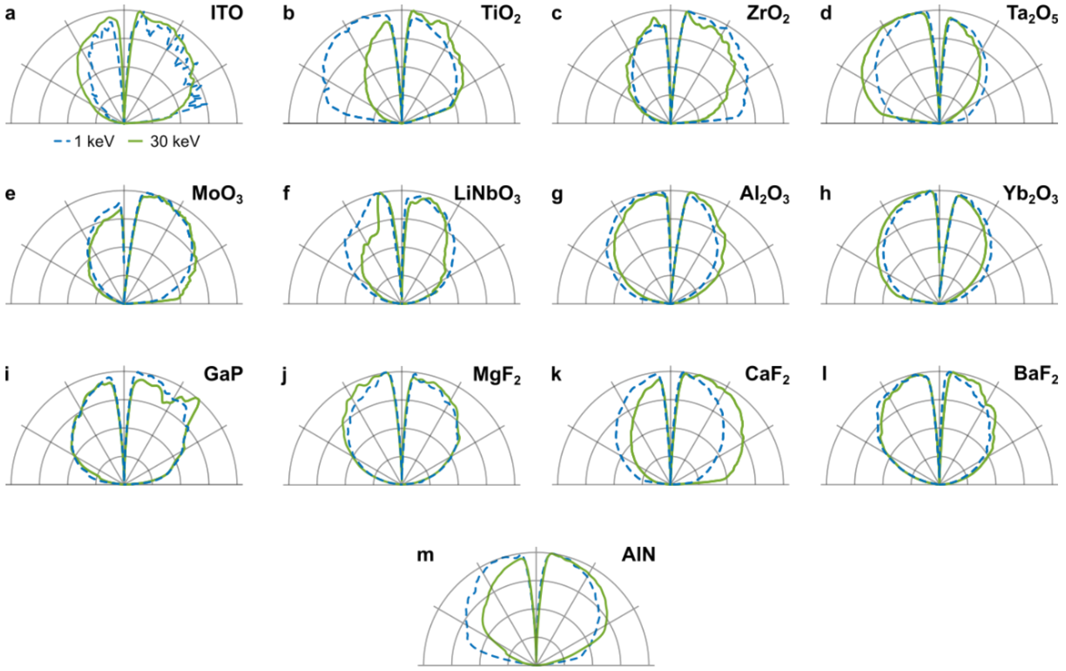

Fig. S17 Angular emission profiles of common photonic materials and optical coatings. **a** ITO, **b** TiO_2, **c** ZrO_2, **d** Ta_2O_5, **e** MoO_3, **f** $LiNbO_3$, **g** α-Al_2O_3, **h** Yb_2O_3, **i** GaP, **j** MgF_2, **k** CaF_2, **l** BaF_2, and **m** AlN.

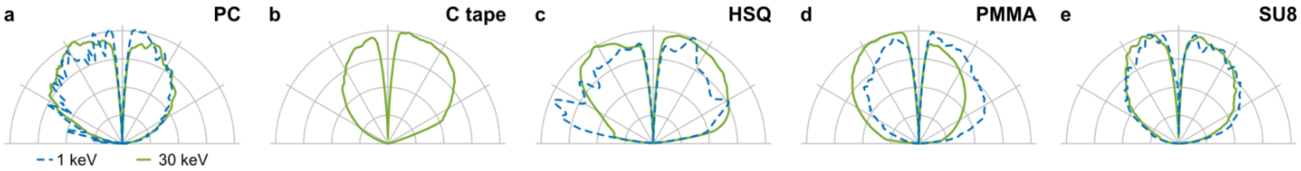

Fig. S18 Angular emission profiles of polymeric and resist materials. **a** PC, **b** carbon tape, **c** HSQ, **d** PMMA, and **e** SU8. We were unable to measure the angular emission profile of carbon tape at 1 keV, as maintaining the necessary beam alignment under these conditions proved too challenging. The HSQ sample's inhomogeneous coverage and uneven electron-beam exposure increase scattering, leading to irregular angular emission profiles.

Self-absorption in the cathodoluminescence from TMD bulk crystals

In this section, we compare the CL spectra of transition metal dichalcogenide (TMD) crystals with extinction coefficients obtained from ellipsometry measurements [2]. Figure S19 presents the CL spectra of TMD crystals alongside the corresponding extinction spectrum. The A and B exciton lines, identified from the extinction spectrum, are marked by gray lines. Table S1 summarizes the spectral dips in the CL spectrum and the exciton positions in the extinction spectrum.

Material	ReS_2		WS_2		WSe_2		$MoSe_2$		MoS_2		3R MoS_2	
	CL	[2]	CL	[2]	CL	[2]	CL	[2]	CL	[2]	CL	[2]
A exciton (**X_A**) Position in nm	814	xx: 829<br>yy: 790	620	629	755	762	799	803	662	675	665	-
B exciton (**X_B**) Position in nm	-	-	514	517	562	563	686	686	604	626	613	-

Table S1 Comparison of position of spectral dips in the CL spectrum with the spectral position of the exciton lines as determined from ellipsometry measurements in Ref. [2].

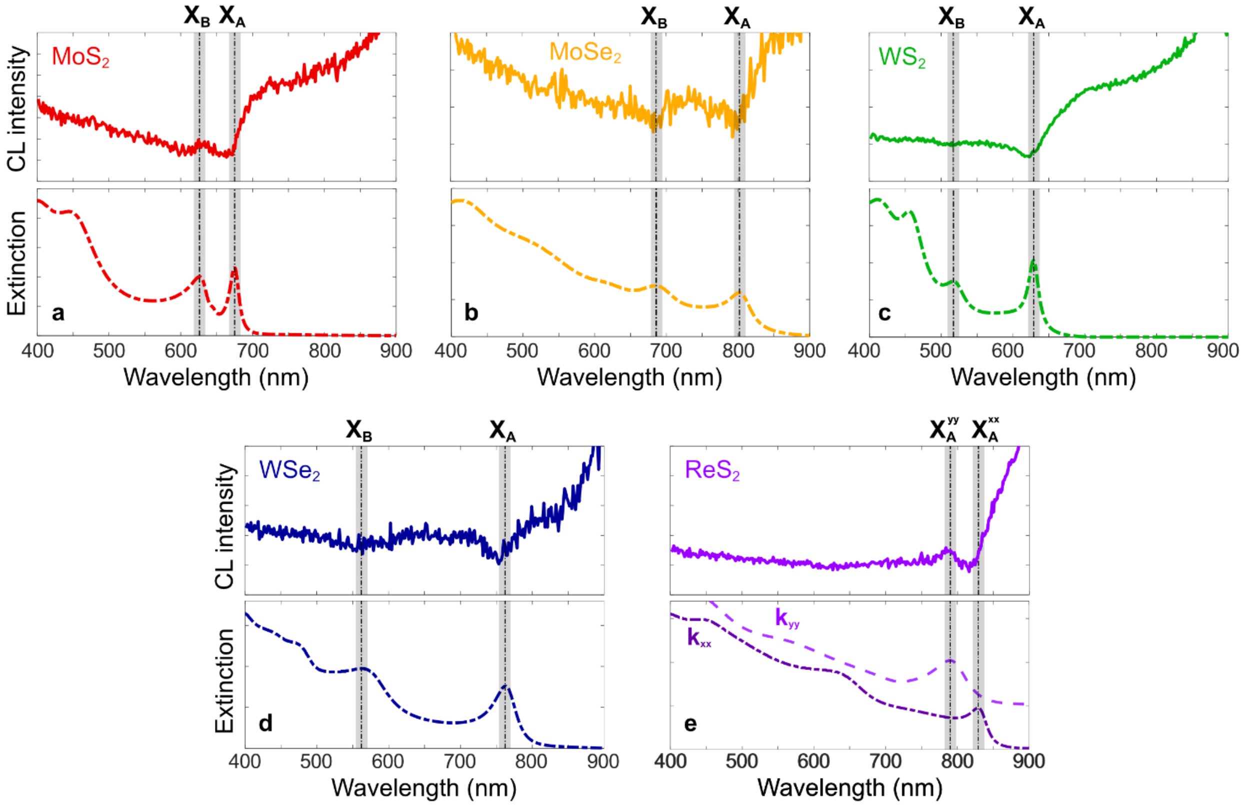

Fig. S19 CL self-absorption in TMD bulk flakes. Cathodoluminescence spectra of the measured semiconducting TMD bulk crystals at 20 keV (**a-d**) and 30 keV (**e**) excitation energy and a beam current of 1.4 nA (Top panel in **a-e**). Lower panel a-e shows the extinction coefficient measurements of thin TMD bulk crystal slabs measured by Munkhbat et al. [2]. The markers represent the position of the A (X_A) and B (X_B) exciton bands.

[2] B. Munkhbat, P. Wróbel, T. J. Antosiewicz, and T. O. Shegai, "Optical Constants of Several Multilayer Transition Metal Dichalcogenides Measured by Spectroscopic Ellipsometry in the 300–1700 nm Range: High Index, Anisotropy, and Hyperbolicity," *ACS Photonics*, vol. 9, no. 7, pp. 2398–2407, Jun. 2022, doi: 10.1021/acsphotonics.2c00433.

Raman spectroscopy

Raman spectra were acquired on a WiTec alpha 300 R confocal Raman microscope, using a 532 nm laser, a 50x objective (Nikon E PLAN 50X 0.75 EPI) and two gratings of 600 l/mm and 1800 l/mm. A suitable laser power was selected for each material.

Materials

We summarize sample preparation procedures for each investigated material along with basic optical characterization (bright-field and dark-field imaging, Raman spectrum), electron-beam microscopy (secondary electron (SE) and backscattered electron (BSE) imaging), and results of Monte Carlo simulations of electron penetration and energy deposition.

Gold (Au)

We synthesized monocrystalline gold flakes (for the fabrication procedure see the references [3,4]) on a silicon substrate. For the measurements we selected a single gold flake. For the intensity averaged measurements, we performed measurements of 5 different gold flakes.

[3] Y. Lebsir, S. Boroviks, M. Thomaschewski, S. I. Bozhevolnyi, and V. A. Zenin, “Ultimate limit for optical losses in gold, revealed by quantitative Near-Field microscopy,” *Nano Letters*, vol. 22, no. 14, pp. 5759–5764, Jul. 2022, doi: 10.1021/acs.nanolett.2c01059.

[4] E. Krauss *et al.*, “Controlled growth of High-Aspect-Ratio Single-Crystalline gold platelets,” *Crystal Growth & Design*, vol. 18, no. 3, pp. 1297–1302, Feb. 2018, doi: 10.1021/acs.cgd.7b00849.

Bright field image

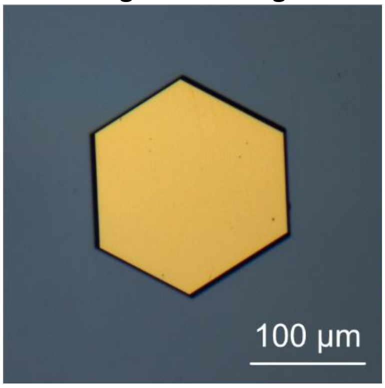

Dark field image

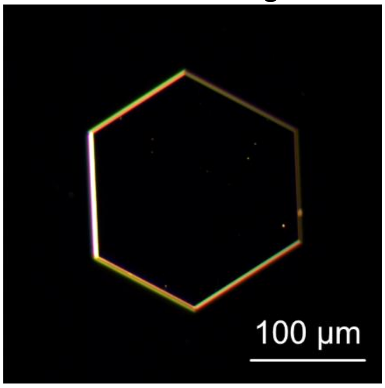

SE 30 keV

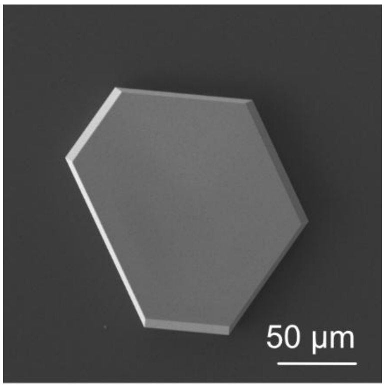

SE 1 keV

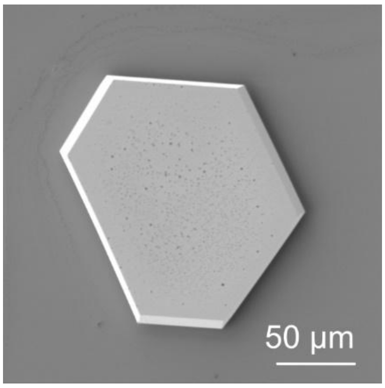

BSE 30 keV

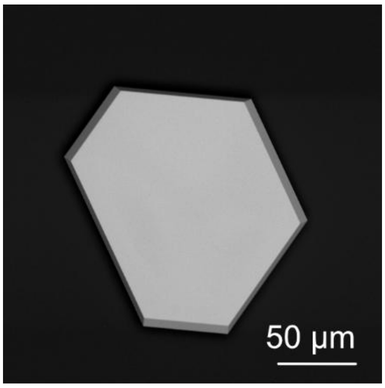

SE 1 keV (close up)

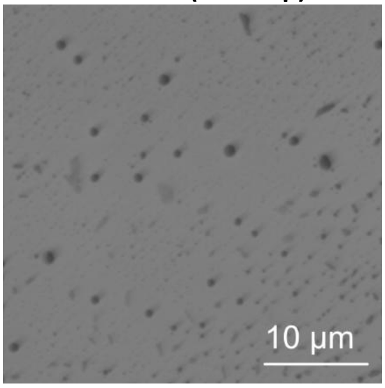

Monte Carlo simulations (CASINO)

d_e		$\langle d_{PE} \rangle$ (d_{stdv})	
1 keV	30 keV	1 keV	30 keV
5.2 nm	59.5 nm	5.7 nm (2.3 nm)	528 nm (192 nm)
$\langle d_{BSE} \rangle$ (d_{stdv})		β (BSE/SE)	
1.5 nm (1.1 nm)	160 nm (105 nm)	0.395	0.480

Material parameters for simulations

ρ (g/cm^3)	Z	Z_{eff}
19.3	79	79

Silver (Ag)

Silver was purchased in the form of silver evaporation pellets from Kurt J. Lesker. The silver has a purity of 99.99%. The selected silver pellet was fixed on a SEM specimen stub with the help of sticking carbon tape.

Bright field image

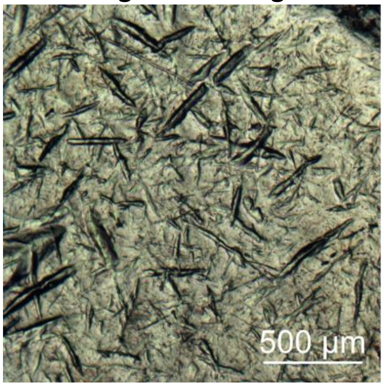

Dark field image

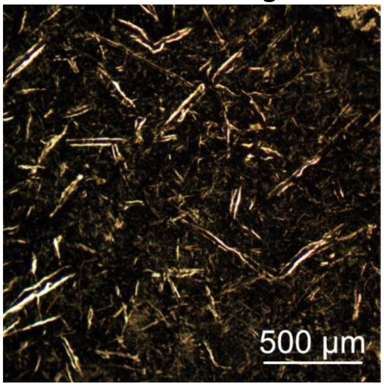

SE 30 keV

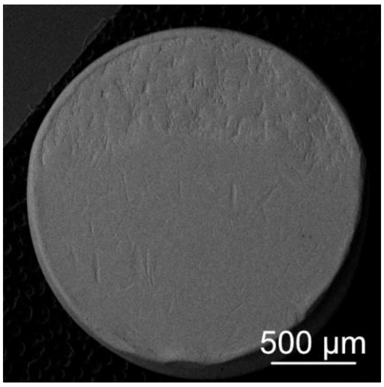

SE 1 keV

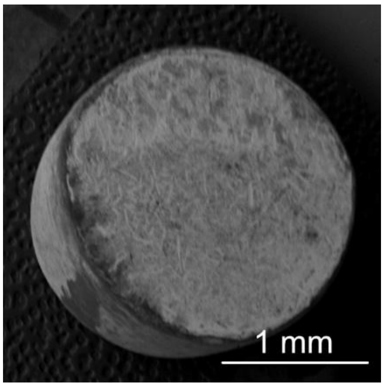

BSE 30 keV

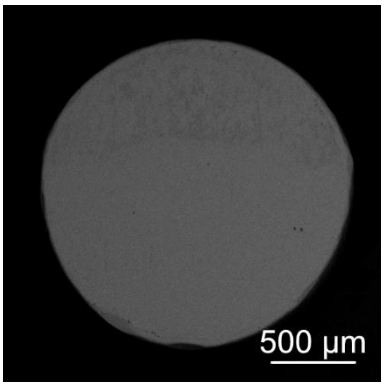

SE 1 keV (close up)

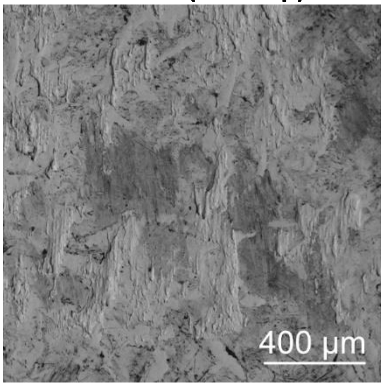

Monte Carlo simulations (CASINO)

d_e		$\langle d_{PE} \rangle$ (d_{stdv})	
1 keV	30 keV	1 keV	30 keV
6.5 nm	80.4 nm	6.6 nm (2.5 nm)	909 nm (331 nm)
$\langle d_{BSE} \rangle$ (d_{stdv})		β (BSE/SE)	
4.2 nm (2.7 nm)	1341 nm (688 nm)	0.362	0.382

Material parameters for simulations

ρ (g/cm^3)	Z	Z_{eff}
10.5	47	47

Copper (Cu)

Copper was purchased in the form of copper evaporation pellets from Kurt J. Lesker. The copper has a purity of 99.99%. The selected copper pellet was fixed on a SEM specimen stub with the help of sticking carbon tape.

Bright field image

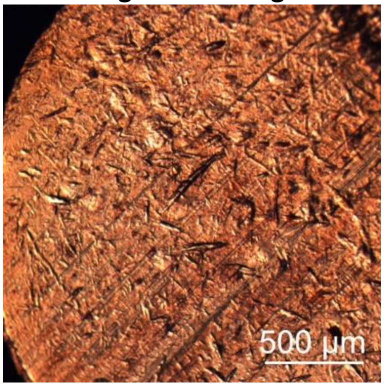

Dark field image

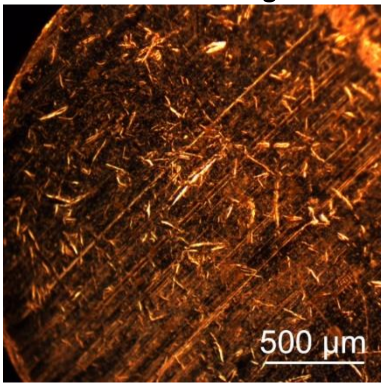

SE 30 keV

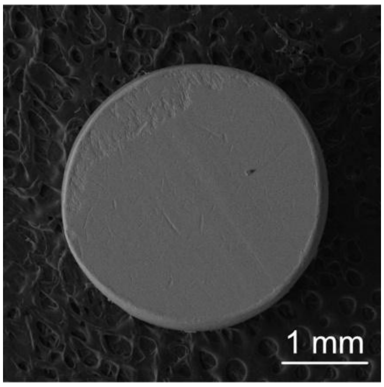

SE 1 keV

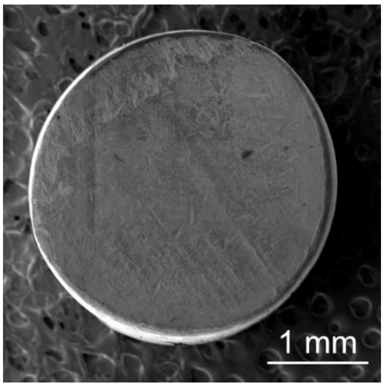

BSE 30 keV

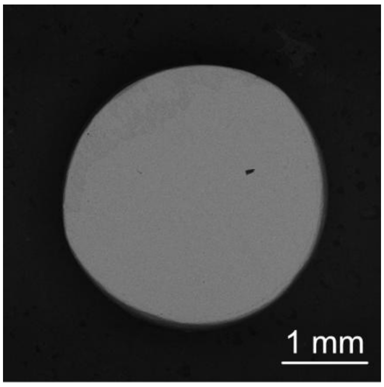

SE 1 keV (close up)

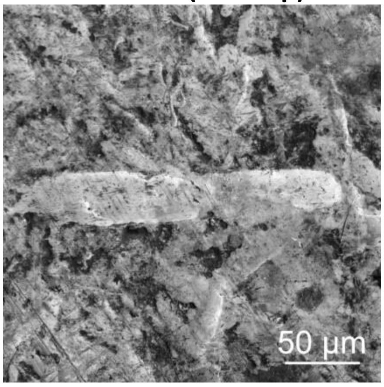

Monte Carlo simulations (CASINO)

d_e		$\langle d_{PE} \rangle$ (d_{stdv})	
1 keV	30 keV	1 keV	30 keV
6.2 nm	89.4 nm	6.1 nm (2.4 nm)	1085 nm (397 nm)
$\langle d_{BSE} \rangle$ (d_{stdv})		β (BSE/SE)	
1.7 nm (1.2 nm)	371 nm (202 nm)	0.307	0.280

Material parameters for simulations

ρ (g/cm^3)	Z	Z_{eff}
8.96	29	29

Aluminum (Al)

Aluminum was purchased in the form of aluminum evaporation pellets from Kurt J. Lesker. The aluminum has a purity of 99.99%. The selected aluminum pellet was fixed on a SEM specimen stub with the help of sticking carbon tape.

Bright field image

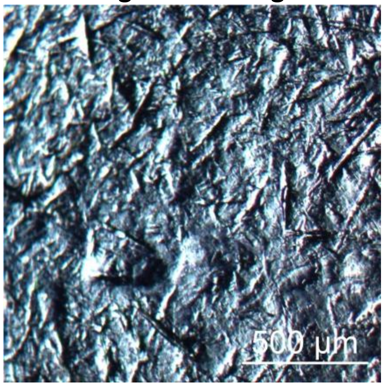

Dark field image

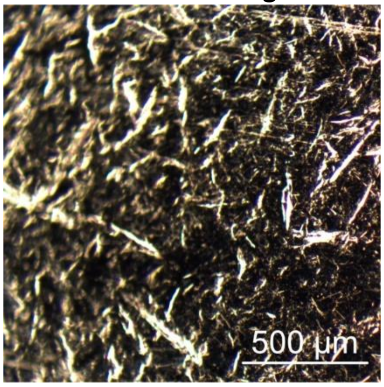

SE 30 keV

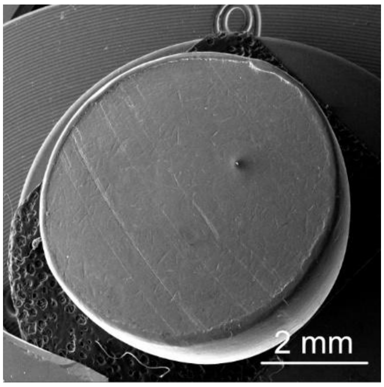

SE 1 keV

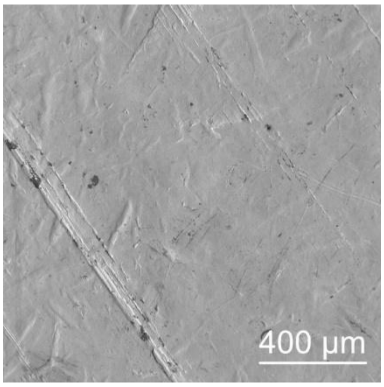

BSE 30 keV

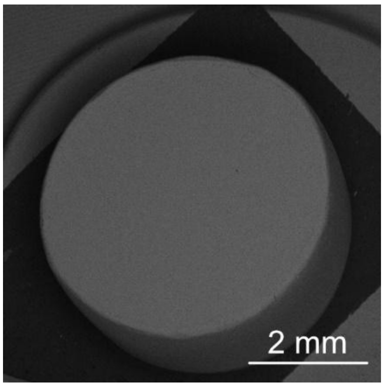

SE 1 keV (close up)

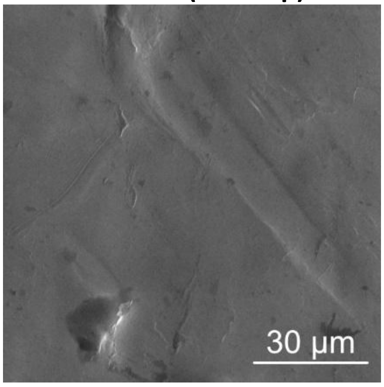

Monte Carlo simulations (CASINO)

d_e		$\langle d_{PE}\rangle$ (d_{stdv})	
1 keV	30 keV	1 keV	30 keV
15.7 nm	345 nm	15.1 nm (5.4 nm)	4000 nm (1357 nm)
$\langle d_{BSE}\rangle$ (d_{stdv})		β (BSE/SE)	
4.2 nm (2.7 nm)	1341 nm (688 nm)	0.181	0.127

Material parameters for simulations

ρ (g/cm^3)	Z	Z_{eff}
2.7	13	13

Titanium (Ti)

Titanium was purchased in the form of titanium evaporation pellets from Kurt J. Lesker. The titanium has a purity of 99.995%. The selected titanium pellet was fixed on a SEM specimen stub with the help of sticking carbon tape.

Bright field image

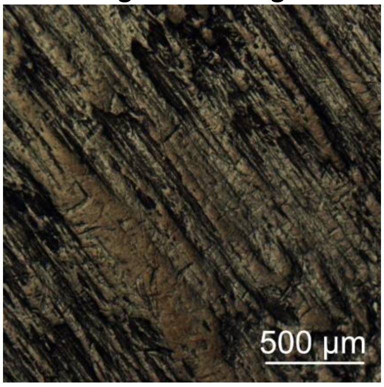

Dark field image

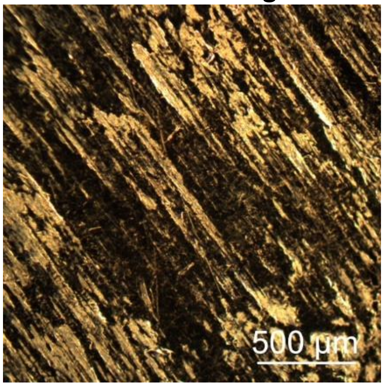

SE 30 keV

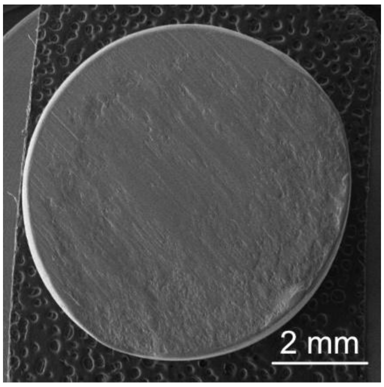

SE 1 keV

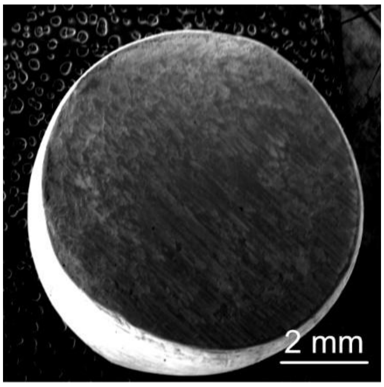

BSE 30 keV

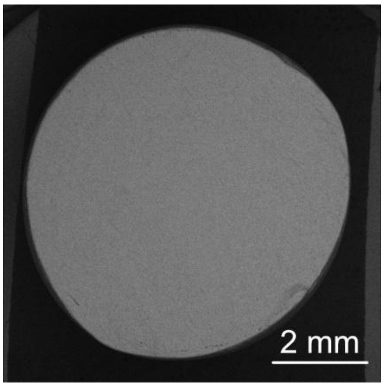

SE 1 keV (close up)

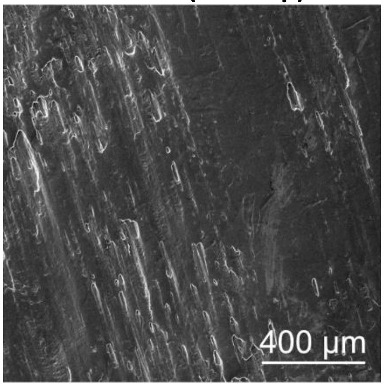

Monte Carlo simulations (CASINO)

d_e		$\langle d_{PE} \rangle$ (d_{stdv})	
1 keV	30 keV	1 keV	30 keV
10.9 nm	186 nm	10.3 nm (3.8 nm)	2216 nm (787 nm)
$\langle d_{BSE} \rangle$ (d_{stdv})		β (BSE/SE)	
2.9 nm (2.0 nm)	773 nm (407 nm)	0.257	0.226

Material parameters for simulations

ρ (g/cm^3)	Z	Z_{eff}
4.5	22	22

Platinum (Pt)

Platinum was purchased in the form of titanium evaporation pellets from Kurt J. Lesker. The platinum has a purity of 99.99%. The selected platinum pellet was fixed on a SEM specimen stub with the help of sticking carbon tape.

Bright field image

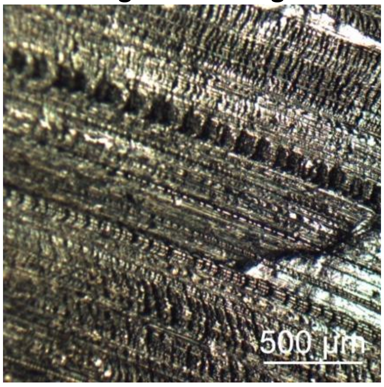

Dark field image

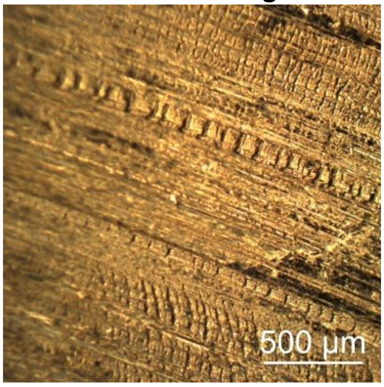

SE 30 keV

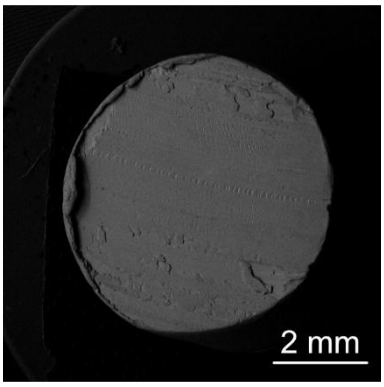

SE 1 keV

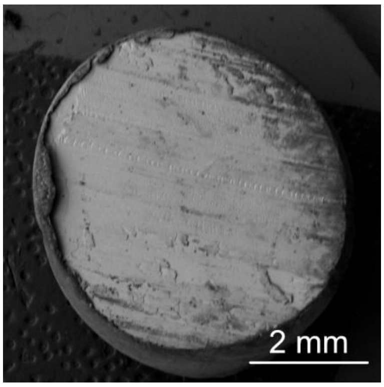

BSE 30 keV

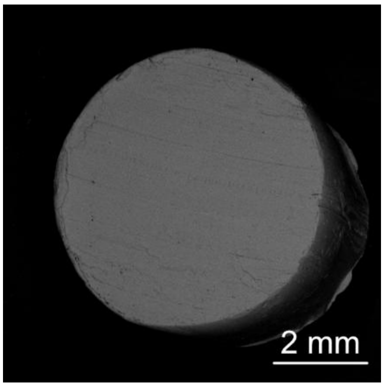

SE 1 keV (close up)

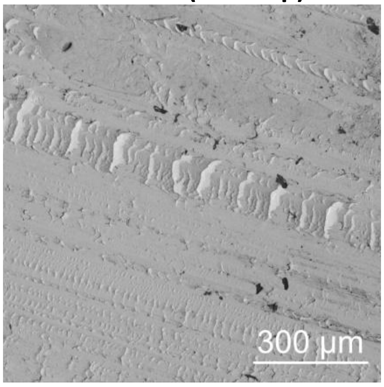

Monte Carlo simulations (CASINO)

d_e		$<d_{PE}>$ (d_{stdv})	
1 keV	30 keV	1 keV	30 keV
4.6 nm	52.9 nm	5.1 nm (2.0 nm)	472 nm (168 nm)
$<d_{BSE}>$ (d_{stdv})		β (BSE/SE)	
1.4 nm (1.0 nm)	144 nm (94 nm)	0.395	0.475

Material parameters for simulations

ρ (g/cm^3)	Z	Z_{eff}
21.45	78	78

Nickel (Ni)

Nickel was purchased in the form of nickel evaporation pellets from Kurt J. Lesker. The nickel has a purity of 99.995%. The selected nickel pellet was fixed on a SEM specimen stub with the help of sticking carbon tape.

Bright field image

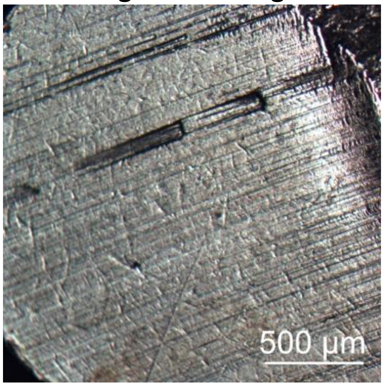

Dark field image

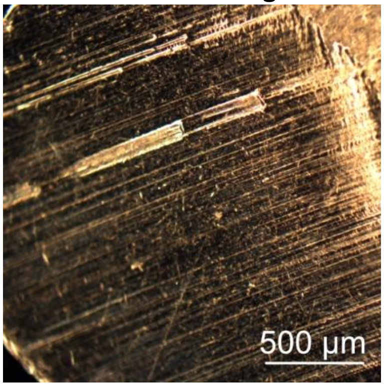

SE 30 keV

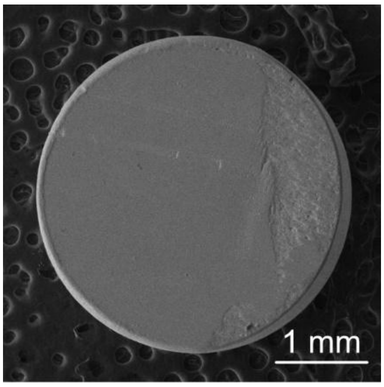

SE 1 keV

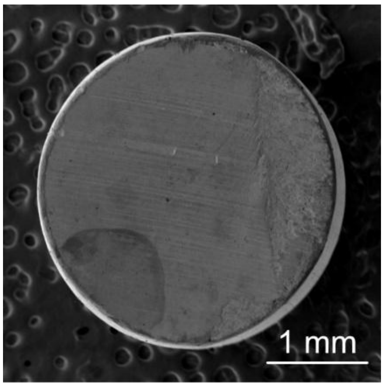

BSE 30 keV

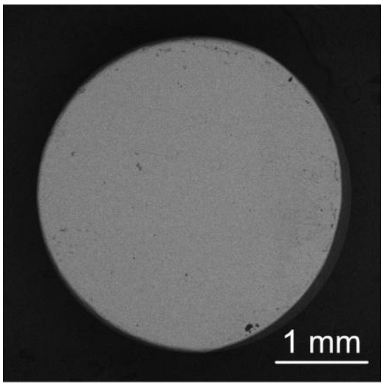

SE 1 keV (close up)

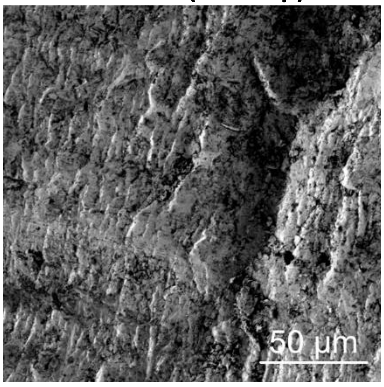

Monte Carlo simulations (CASINO)

d_e		$<d_{PE}>$ (d_{stdv})	
1 keV	30 keV	1 keV	30 keV
5.8 nm	102 nm	5.7 nm (2.2 nm)	1048 nm (381 nm)
$<d_{BSE}>$ (d_{stdv})		β (BSE/SE)	
1.6 nm (1.1 nm)	361 nm (195 nm)	0.300	0.275

Material parameters for simulations

ρ (g/cm^3)	Z	Z_{eff}
8.90	28	8.90

Chromium (Cr)

Chromium was purchased in the form of chromium evaporation pieces from Kurt J. Lesker. The chromium has a purity of 99.95%. The selected chromium piece was fixed on a SEM specimen stub with the help of sticking carbon tape.

Bright field image

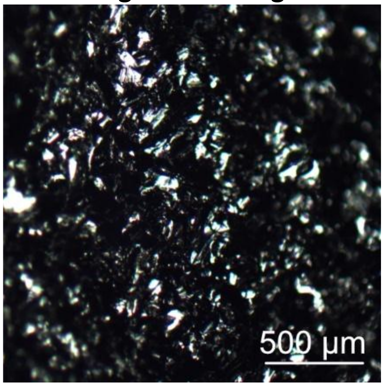

Dark field image

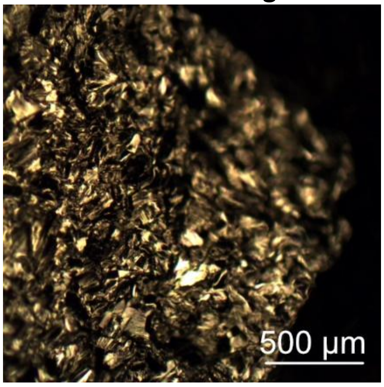

SE 30 keV

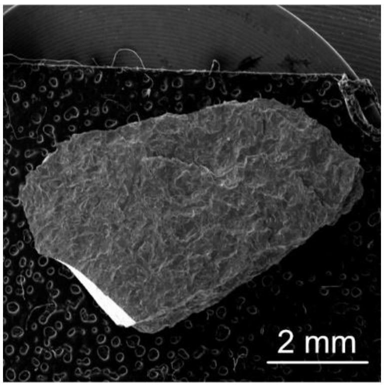

SE 1 keV

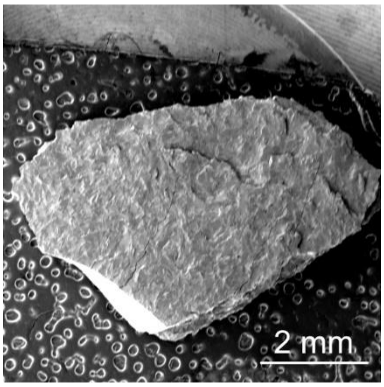

BSE 30 keV

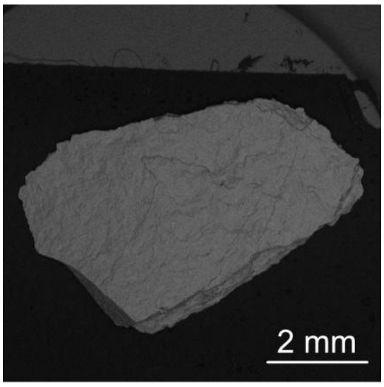

SE 1 keV (close up)

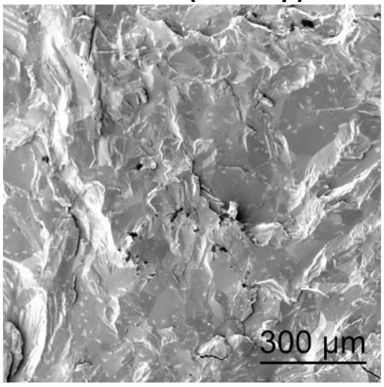

Monte Carlo simulations (CASINO)

d_e		<d_{PE}> (d_{stdv})	
1 keV	30 keV	1 keV	30 keV
7.1 nm	124 nm	6.7 nm (2.5 nm)	1373 nm (500 nm)
<d_{BSE}> (d_{stdv})		β (BSE/SE)	
1.9 nm (1.3 nm)	475 nm (252 nm)	0.274	0.244

Material parameters for simulations

ρ (g/cm^3)	Z	Z_{eff}
7.93	24	24

Silicon (Si)

We used Si wafers from MicroChemicals GmbH (p-type (boron), (100), 500 µm thick), which we cleaved into smaller pieces, and subsequently mounted them on SEM stubs using conductive carbon tape.

Bright field image

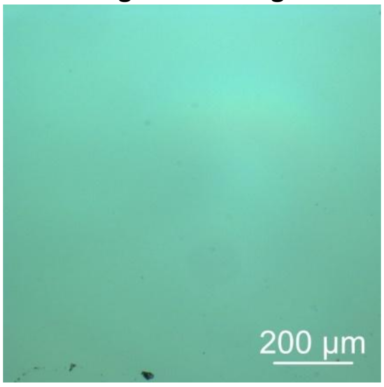

Dark field image

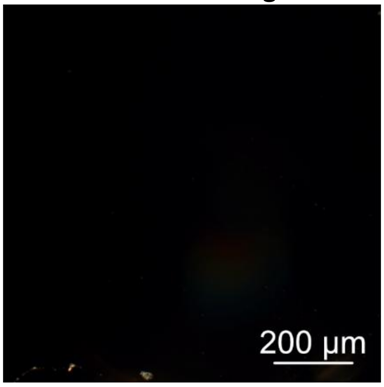

SE 30 keV

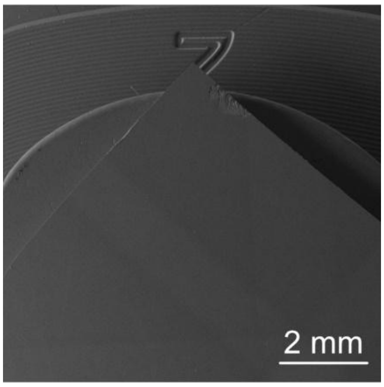

SE 1 keV

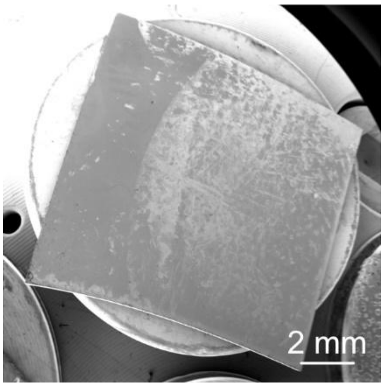

BSE 30 keV

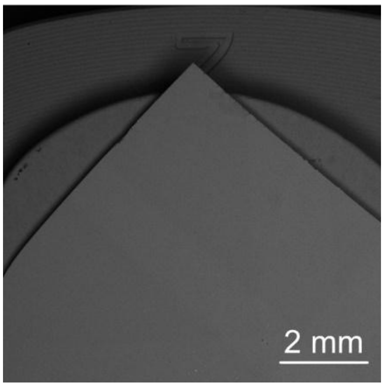

SE 1 keV (close up)

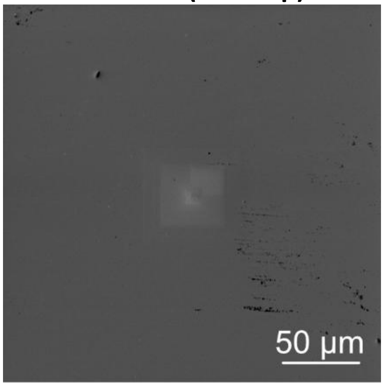

Monte Carlo simulations (CASINO)

d_e		$<d_{PE}>$ (d_{stdv})	
1 keV	30 keV	1 keV	30 keV
18.9 nm	391 nm	17.0 nm (6.1 nm)	4393 nm (1514 nm)
$<d_{BSE}>$ (d_{stdv})		β (BSE/SE)	
4.8 nm (3.1 nm)	1491 nm (762 nm)	0.191	0.141

Material parameters for simulations

ρ (g/cm^3)	Z	Z_{eff}
2.33	14	14

Raman spectrum

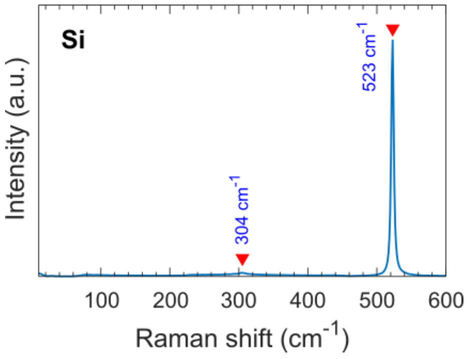

Si exhibits a prominent Raman-active vibrational mode at approximately 523 cm^{-1}, corresponding to the first-order transverse optical (TO) phonon mode near the Brillouin zone center. The peak around 304 cm^{-1} is attributed to the second-order transverse acoustic (2TA) phonon mode near the X point of the Brillouin zone [5].

[5] J. H. Parker, D. W. Feldman, and M. Ashkin, “Raman Scattering by Silicon and Germanium,” Physical Review, vol. 155, no. 3. American Physical Society (APS), pp. 712–714, Mar. 15, 1967. doi: 10.1103/physrev.155.712.

Germanium (Ge)

We used commercial Ge wafers, which we cleaved into smaller pieces, and subsequently mounted them on SEM stubs using conductive carbon tape.

Bright field image

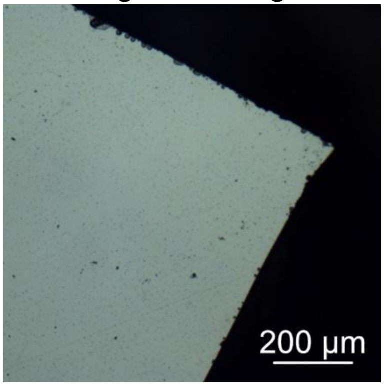

Dark field image

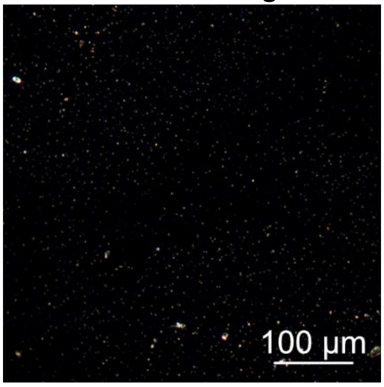

SE 30 keV

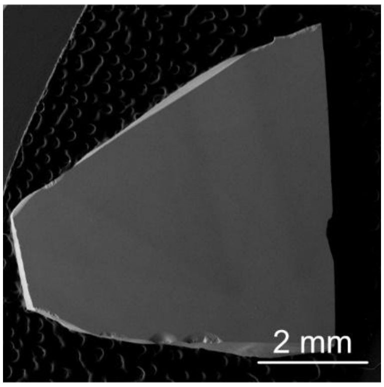

SE 1 keV

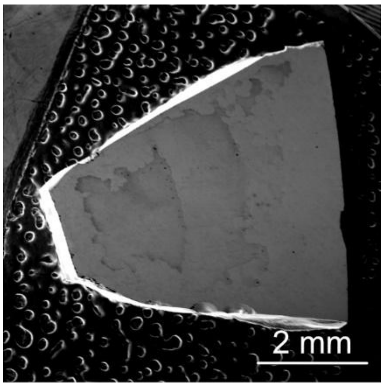

BSE 30 keV

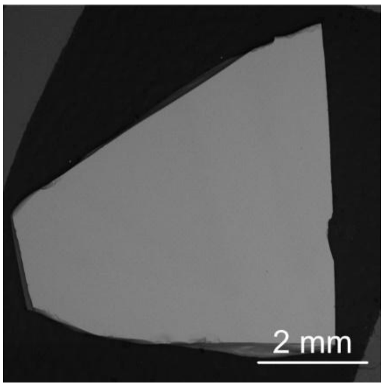

SE 1 keV (close up)

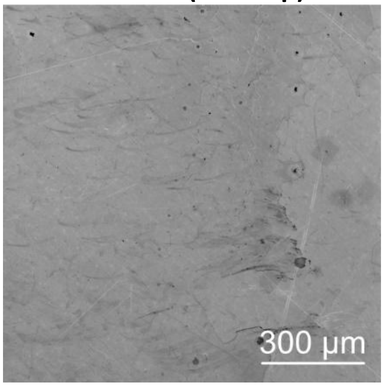

Monte Carlo simulations (CASINO)

d_e		$\langle d_{PE} \rangle$ (d_{stdv})	
1 keV	30 keV	1 keV	30 keV
11.2 nm	146 nm	11.1 nm (4.3 nm)	1878 nm (695 nm)
$\langle d_{BSE} \rangle$ (d_{stdv})		β (BSE/SE)	
3.1 nm (2.2 nm)	638 nm (355 nm)	0.318	0.307

Material parameters for simulations

ρ (g/cm^3)	Z	Z_{eff}
5.32	32	32

Raman spectrum

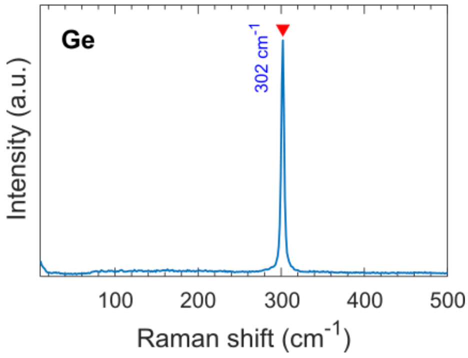

Ge exhibits a Raman-active vibrational mode around 302 cm^{-1}, corresponding to the first-order transverse optical (TO) phonon mode near the Brillouin zone center [5].

Bi_2Te_3

Bi_2Te_3 bulk crystals were purchased from 2D-Semiconductors. From the purchased crystals one was selected and fixed on a SEM specimen stub with the help of sticking carbon tape.

Bright field image

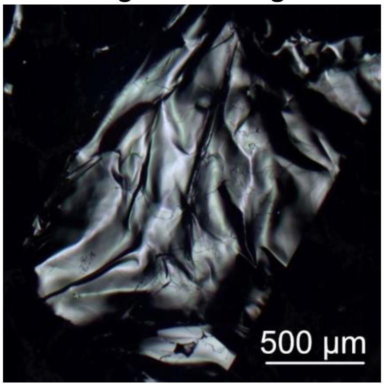

Dark field image

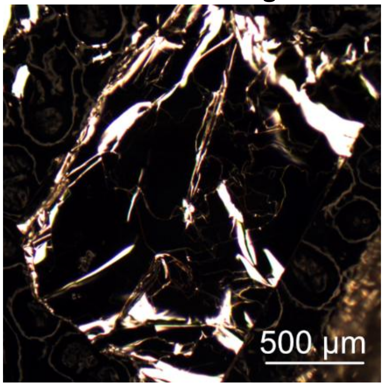

SE 30 keV

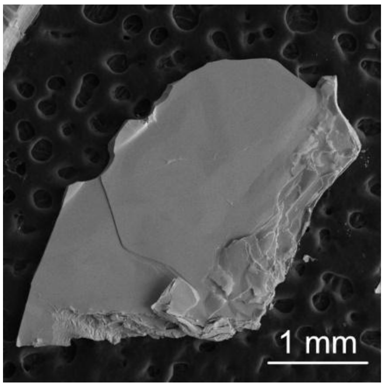

SE 1 keV

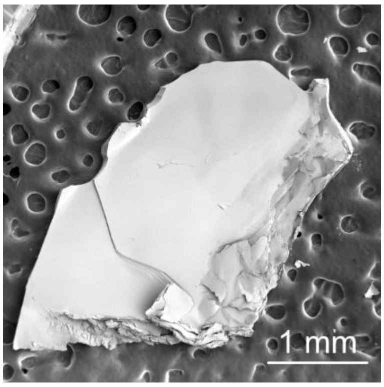

BSE 30 keV

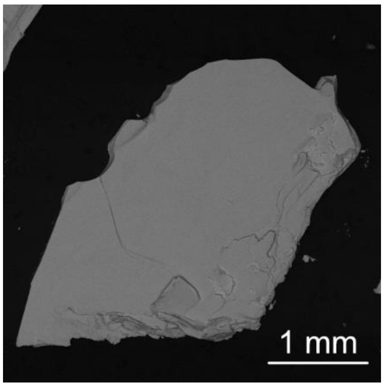

SE 1 keV (close up)

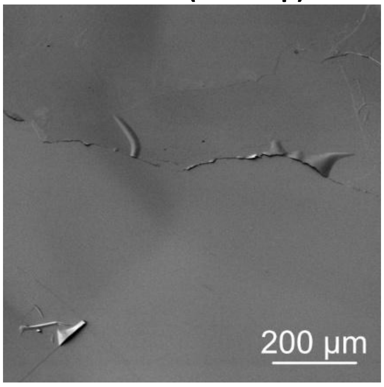

Monte Carlo simulations (CASINO)

d_e		$<d_{PE}>$ (d_{stdv})	
1 keV	30 keV	1 keV	30 keV
11.2 nm	116 nm	11.9 nm (4.9 nm)	1323 nm (489 nm)
$<d_{BSE}>$ (d_{stdv})		β (BSE/SE)	
3.2 nm (2.4 nm)	408 nm (260 nm)	0.378	0.451

Material parameters for simulations

ρ (g/cm^3)	Z	Z_{eff}
7.74	Bi: 83 Te: 52	64.21

Raman spectrum

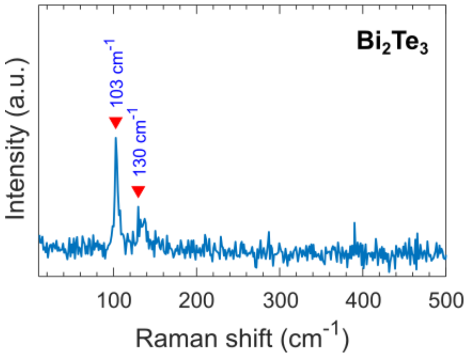

The main Raman peaks are observed at approximately 103 cm^{-1} (E_{g2}) and 130 cm^{-1} (A_{1g2}), where the E_g^2 mode corresponds to in-plane vibrational motion of Te and Bi atoms, and the A_{1g2} mode arises from out-of-plane symmetric stretching of Te atoms along the c-axis [6].

[6] K. M. F. Shahil, M. Z. Hossain, V. Goyal, and A. A. Balandin, “Micro-Raman spectroscopy of mechanically exfoliated few-quintuple layers of Bi2Te3, Bi2Se3, and Sb2Te3 materials,” Journal of Applied Physics, vol. 111, no. 5. AIP Publishing, Mar. 01, 2012. doi: 10.1063/1.3690913.

Sb_2S_3

Sb_2S_3 bulk crystals were purchased from POLYTEKNIK. From the purchased crystals one was selected and fixed on a SEM specimen stub with the help of sticking carbon tape.

Bright field image

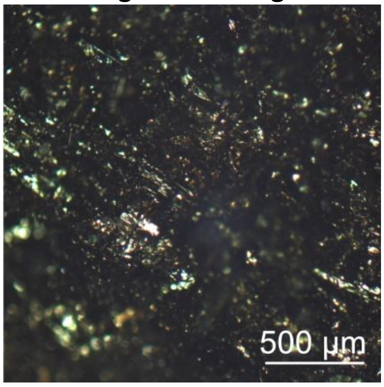

Dark field image

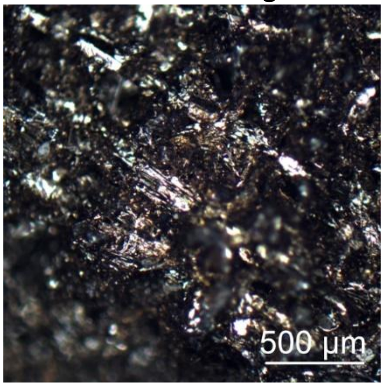

SE 30 keV

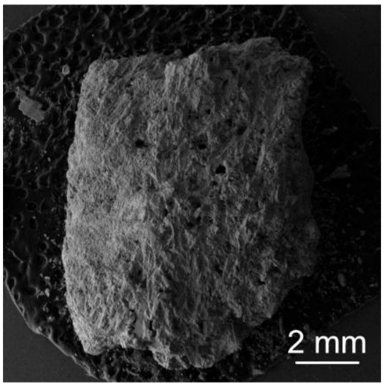

SE 1 keV

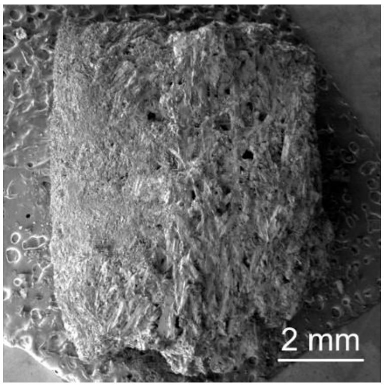

BSE 30 keV

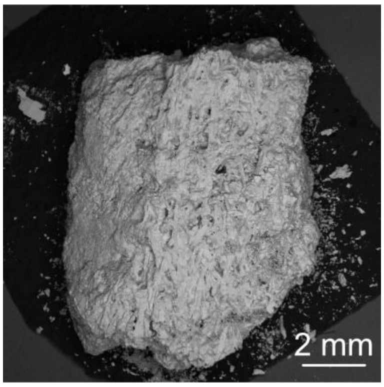

SE 1 keV (close up)

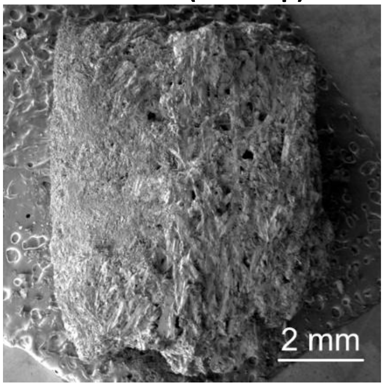

Monte Carlo simulations (CASINO)

d_e		$\langle d_{PE} \rangle$ (d_{stdv})	
1 keV	30 keV	1 keV	30 keV
13.4 nm	192 nm	13.1 nm (4.9 nm)	2118 nm (784 nm)
$\langle d_{BSE} \rangle$ (d_{stdv})		β (BSE/SE)	
3.7 nm (2.6 nm)	700 nm (406 nm)	0.305	0.337

Material parameters for simulations

ρ (g/cm^3)	Z	Z_{eff}
4.56	Sb: 51 S: 16	7.74

Raman spectrum

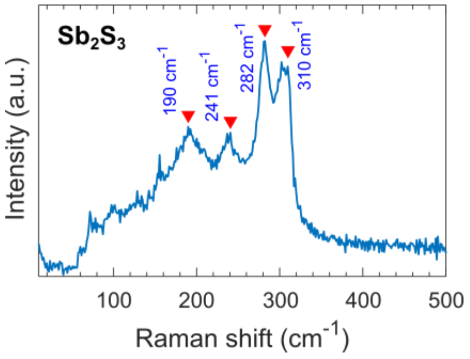

The main Raman peaks are observed at approximately 190 cm^{-1} and 241 cm^{-1}, which are generally assigned to the Sb_2S_3 phase. Additionally, peaks around 282 cm^{-1} and 310 cm^{-1} are attributed to the symmetric vibration of the Sb_2S_3 pyramidal units with C_{3v} symmetry [7].

[7] A. Chihi, “Annealing effect on Sb2S3/c-Si structure for photovoltaic applications,” Applied Physics A, vol. 130, no. 8. Springer Science and Business Media LLC, Jul. 02, 2024. doi: 10.1007/s00339-024-07692-4.

2H-MoS_2

2H-MoS_2 bulk crystals were purchased from HQ-Graphene. From the purchased crystals one was selected and fixed on a SEM specimen stub with the help of sticking carbon tape.

Bright field image

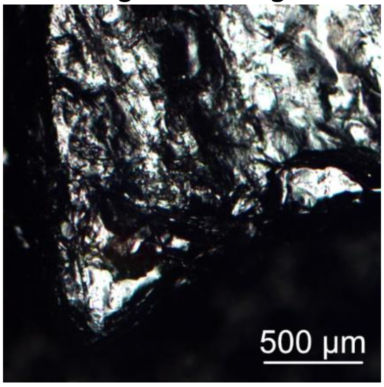

Dark field image

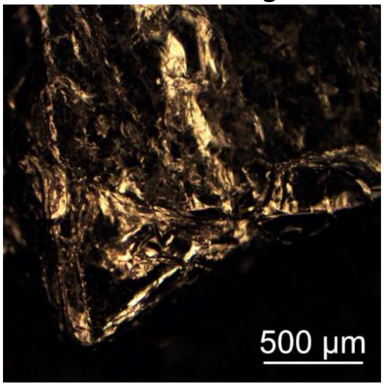

SE 30 keV

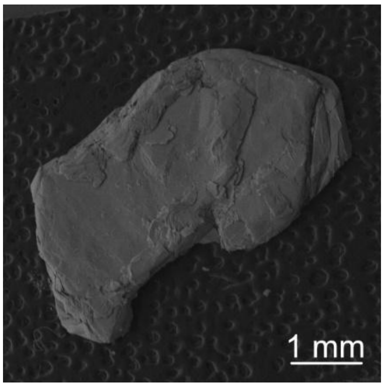

SE 1 keV

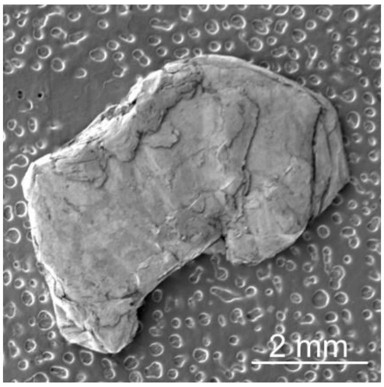

BSE 30 keV

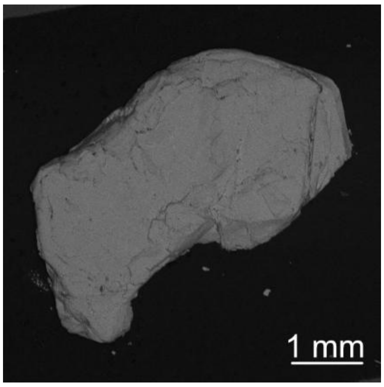

SE 1 keV (close up)

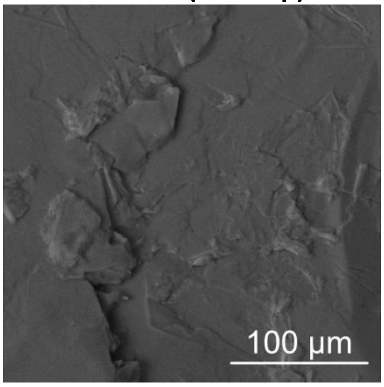

Monte Carlo simulations (CASINO)

d_e		$<d_{PE}>$ (d_{stdv})	
1 keV	30 keV	1 keV	30 keV
10.8 nm	152 nm	10.3 nm (3.8 nm)	1891 nm (695 nm)
$<d_{BSE}>$ (d_{stdv})		β (BSE/SE)	
2.9 nm (2.0 nm)	640 nm (356 nm)	0.277	0.287

Material parameters for simulations

ρ (g/cm^3)	Z	Z_{eff}
5.06	Mo: 42 S: 16	23.97

Raman spectrum

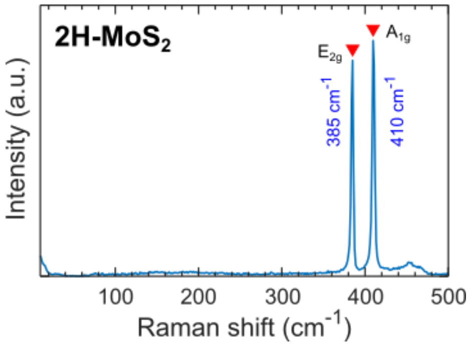

We observe Raman peaks at 385 cm^{-1} and 410 cm^{-1} in 2H-MoS_2. The peak at 384 cm^{-1} is attributed to the E_{2g} mode, representing the in-plane vibration of molybdenum and sulfur atoms. The peak at 411 cm^{-1} corresponds to the A1g mode, which arises from the out-of-plane vibration of sulfur atoms [8].

[8] X. Zhang, X.-F. Qiao, W. Shi, J.-B. Wu, D.-S. Jiang, and P.-H. Tan, "Phonon and Raman scattering of two-dimensional transition metal dichalcogenides from monolayer, multilayer to bulk material," Chemical Society Reviews, vol. 44, no. 9. Royal Society of Chemistry (RSC), pp. 2757–2785, 2015. doi: 10.1039/c4cs00282b.

3R-MoS_2

3R-MoS_2 bulk crystals were purchased from HQ-Graphene. From the purchased crystals one was selected and fixed on a SEM specimen stub with the help of sticking carbon tape.

Bright field image

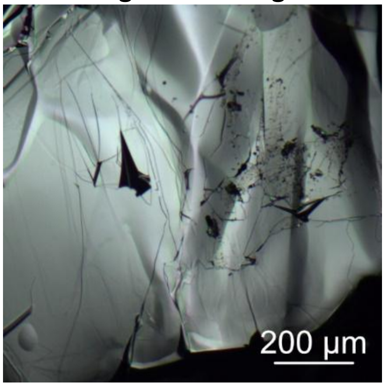

Dark field image

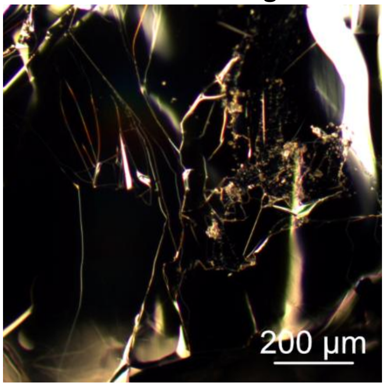

SE 30 keV

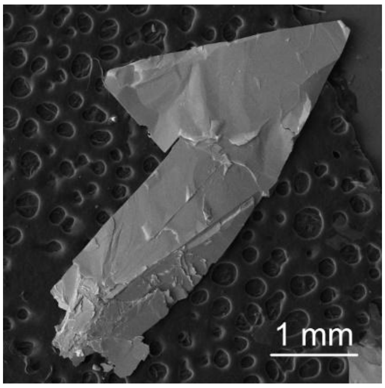

SE 1 keV

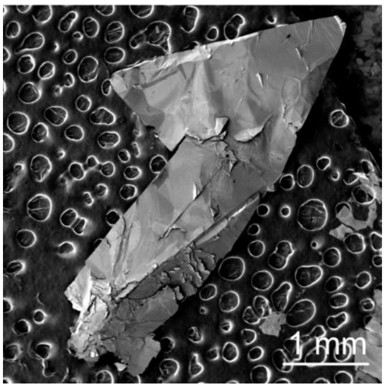

BSE 30 keV

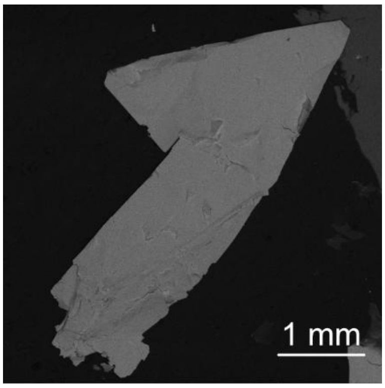

SE 1 keV (close up)

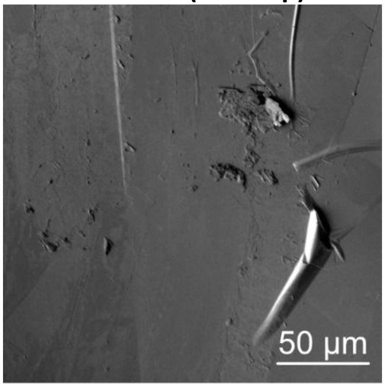

Monte Carlo simulations (CASINO)

d_e		$\langle d_{PE} \rangle$ (d_{stdv})	
1 keV	30 keV	1 keV	30 keV
10.8 nm	152 nm	10.3 nm (3.8 nm)	1891 nm (695 nm)
$\langle d_{BSE} \rangle$ (d_{stdv})		β (BSE/SE)	
2.9 nm (2.0 nm)	640 nm (356 nm)	0.277	0.287

Material parameters for simulations

ρ (g/cm^3)	Z	Z_{eff}
5.06	Mo: 42 S: 16	23.97

Raman spectrum

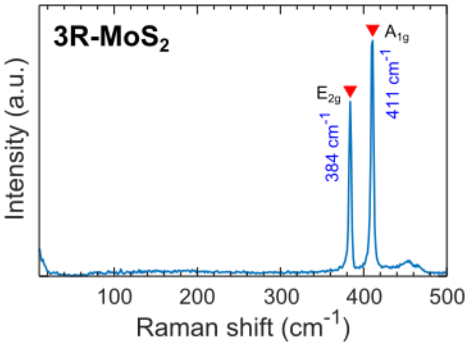

We observe Raman peaks at 384 cm^{-1} and 411 cm^{-1} in 3R-MoS_2. The peak at 384 cm^{-1} is attributed to the E2g mode, representing the in-plane vibration of molybdenum and sulfur atoms. The peak at 411 cm^{-1} corresponds to the A1g mode, which arises from the out-of-plane vibration of sulfur atoms [8].

2H-$MoSe_2$

2H-$MoSe_2$ bulk crystals were purchased from HQ-Graphene. From the purchased crystals one was selected and fixed on a SEM specimen stub with the help of sticking carbon tape.

Bright field image

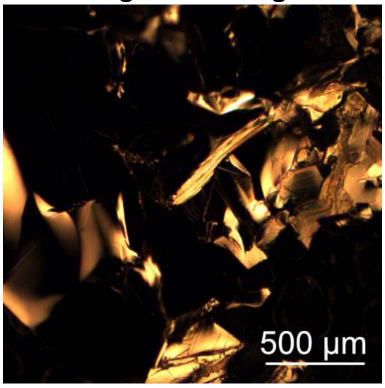

Dark field image

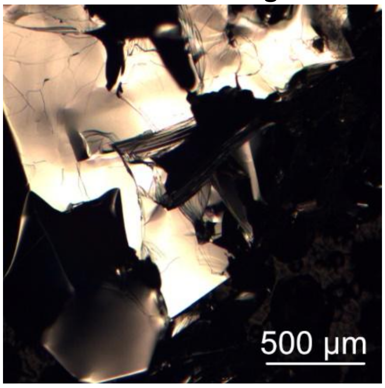

SE 30 keV

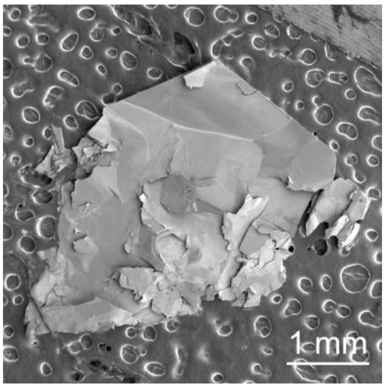

SE 1 keV

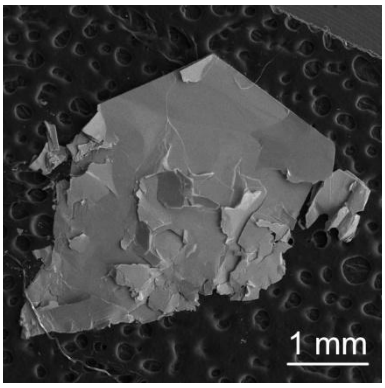

BSE 30 keV

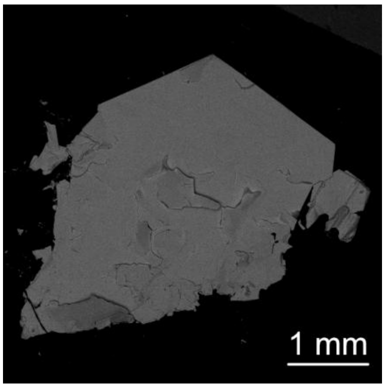

SE 1 keV (close up)

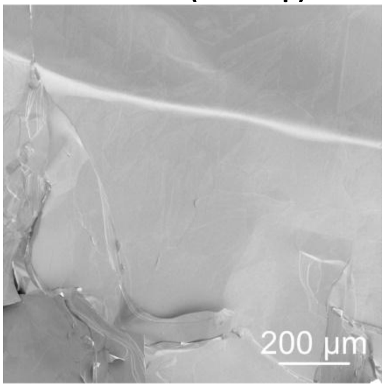

Monte Carlo simulations (CASINO)

d_e		$<d_{PE}>$ (d_{stdv})	
1 keV	30 keV	1 keV	30 keV
9.0 nm	124 nm	9.2 nm (3.5 nm)	1436 nm (532 nm)
$<d_{BSE}>$ (d_{stdv})		β (BSE/SE)	
2.5 nm (1.8 nm)	480 nm (273 nm)	0.331	0.335

Material parameters for simulations

ρ (g/cm^3)	Z	Z_{eff}
6.90	Mo: 42 Se: 34	36.71

Raman spectrum

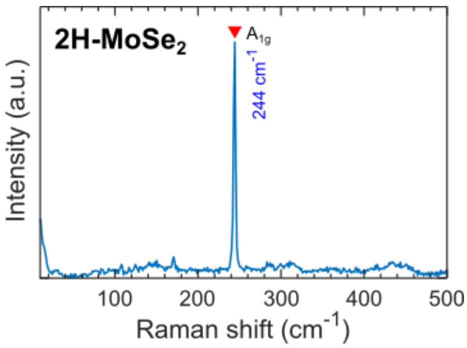

We observe a Raman peak at 244 cm^{-1}, which correspond to the characteristic vibrational mode of 2H-$MoSe_2$. This peak is attributed to the A1g mode, which arises from the out-of-plane vibration of selenium atoms [9].

[9] D. J. Late, S. N. Shirodkar, U. V. Waghmare, V. P. Dravid, and C. N. R. Rao, "Thermal Expansion, Anharmonicity and Temperature-Dependent Raman Spectra of Single- and Few-Layer MoSe2 and WSe2," ChemPhysChem, vol. 15, no. 8. Wiley, pp. 1592–1598, Apr. 2014. doi: 10.1002/cphc.201400020.

2H-WS_2

2H-WS_2 bulk crystals were purchased from HQ-Graphene. From the purchased crystals one was selected and fixed on a SEM specimen stub with the help of sticking carbon tape.

Bright field image

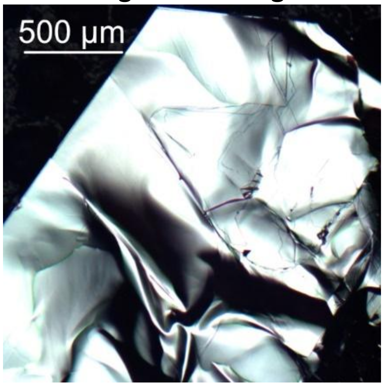

Dark field image

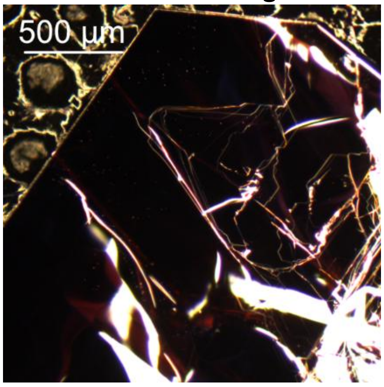

SE 30 keV

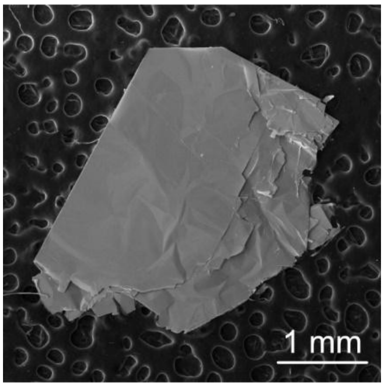

SE 1 keV

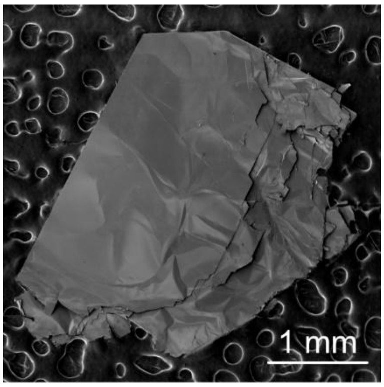

BSE 30 keV

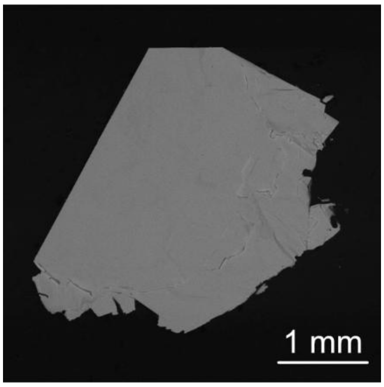

SE 1 keV (close up)

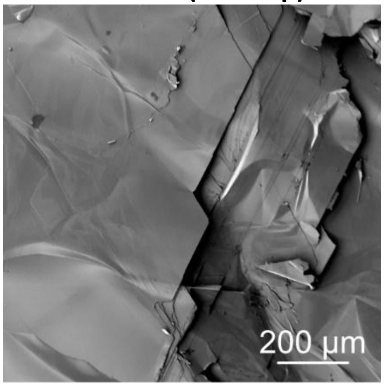

Monte Carlo simulations (CASINO)

d_e		$\langle d_{PE} \rangle$ (d_{stdv})	
1 keV	30 keV	1 keV	30 keV
9.7 nm	123 nm	9.7 nm (3.7 nm)	1280 nm (473 nm)
$\langle d_{BSE} \rangle$ (d_{stdv})		β (BSE/SE)	
2.5 nm (1.8 nm)	480 nm (273 nm)	0.314	0.398

Material parameters for simulations

ρ (g/cm^3)	Z	Z_{eff}
7.60	W: 74 S: 16	32.68

Raman spectrum

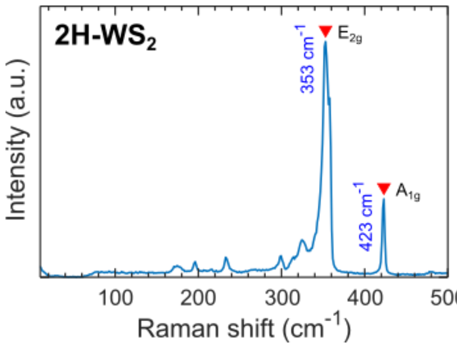

We observe Raman peaks at 353 cm^{-1} and 423 cm^{-1}, which correspond to the characteristic vibrational modes of 2H-WS_2. The peak at 353 cm^{-1} is attributed to the E_2g mode, representing the in-plane vibration of tungsten and sulfur atoms. The peak at 423 cm^{-1} corresponds to the A_1g mode, which arises from the out-of-plane vibration of sulfur atoms [8].

2H-WSe_2

2H-WSe2 bulk crystals were purchased from HQ-Graphene. From the purchased crystals one was selected and fixed on a SEM specimen stub with the help of sticking carbon tape.

Bright field image

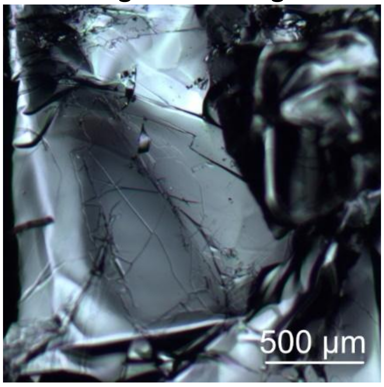

Dark field image

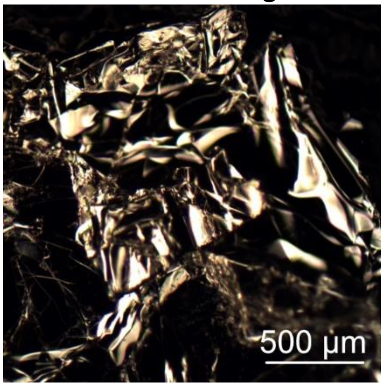

SE 30 keV

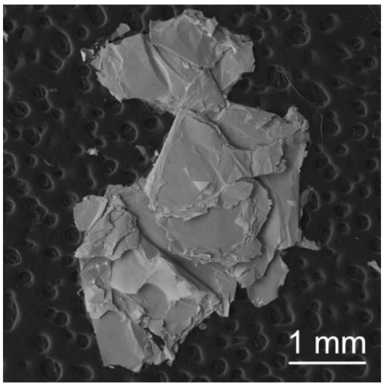

SE 1 keV

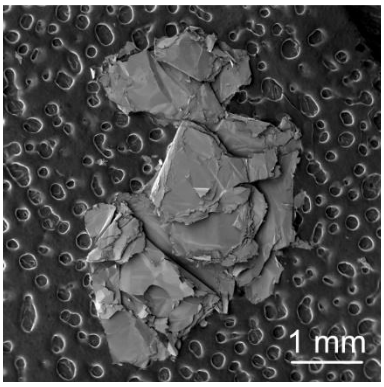

BSE 30 keV

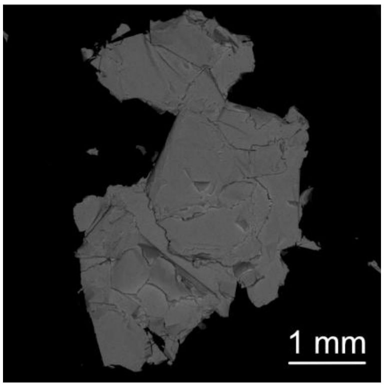

SE 1 keV (close up)

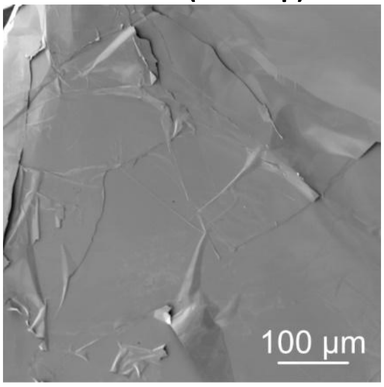

Monte Carlo simulations (CASINO)

d_e		$\langle d_{PE} \rangle$ (d_{stdv})	
1 keV	30 keV	1 keV	30 keV
8.3 nm	95.6 nm	8.5 nm (3.2 nm)	1085 nm (399 nm)
$\langle d_{BSE} \rangle$ (d_{stdv})		β (BSE/SE)	
2.3 nm (1.7 nm)	347 nm (213 nm)	0.354	0.403

Material parameters for simulations

ρ (g/cm^3)	Z	Z_{eff}
9.2	W: 74 Se: 34	46.77

Raman spectrum

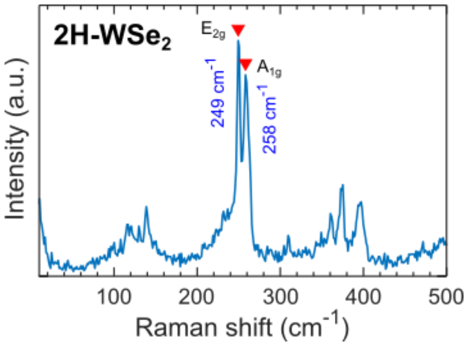

We observe Raman peaks at 249 cm^{-1} and 258 cm^{-1}, which correspond to the characteristic vibrational modes of 2H-WSe_2. The peak at 249 cm^{-1} is attributed to the E2g mode, representing the in-plane vibration of tungsten and selenium atoms. The peak at 258 cm^{-1} corresponds to the A1g mode, which arises from the out-of-plane vibration of selenium atoms [8].

1T'-ReS_2

1T'-ReS_2 bulk crystals were purchased from HQ-Graphene. From the purchased crystals one was selected and fixed on a SEM specimen stub with the help of sticking carbon tape.

Bright field image

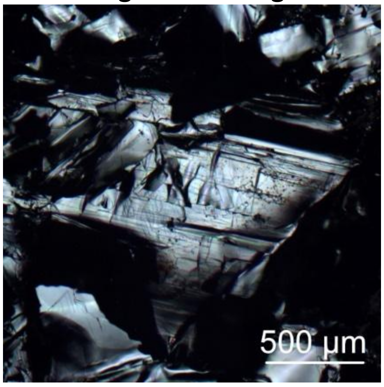

Dark field image

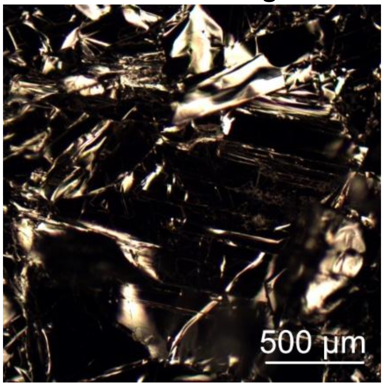

SE 30 keV

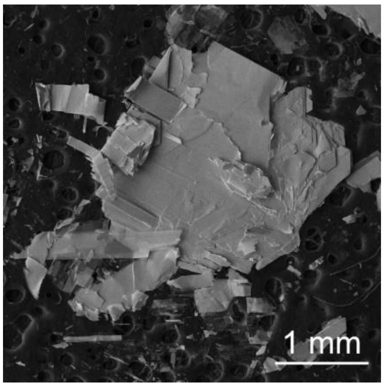

SE 1 keV

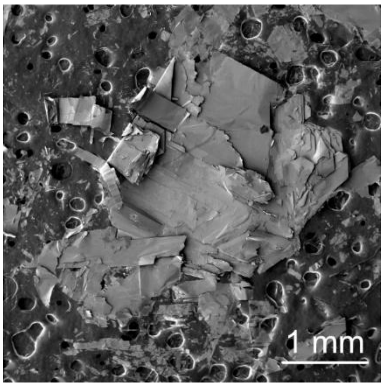

BSE 30 keV

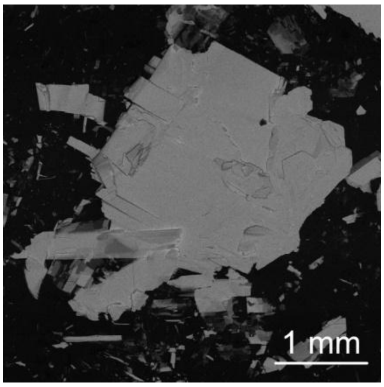

SE 1 keV (close up)

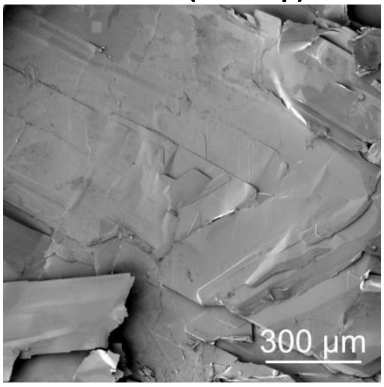

Monte Carlo simulations (CASINO)

d_e		$<d_{PE}>$ (d_{stdv})	
1 keV	30 keV	1 keV	30 keV
9.7 nm	105.3 nm	9.7 nm (3.7 nm)	1268 nm (455 nm)
$<d_{BSE}>$ (d_{stdv})		β (BSE/SE)	
2.6 nm (1.9 nm)	404 nm (252 nm)	0.314	0.401

Material parameters for simulations

ρ (g/cm³)	Z	Z_{eff}
7.6	Re: 75 S: 16	32.94

Raman spectrum

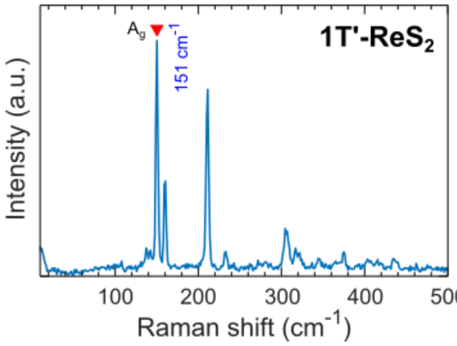

We observe a Raman peak at 151 cm^{-1}, which corresponds to an Ag-like vibrational mode of 1T'-ReS_2. This mode is associated with the in-plane vibrational motion of rhenium and sulfur atoms [10].

[10] Y. Feng et al., "Raman vibrational spectra of bulk to monolayer ReS2 with lower symmetry," Physical Review B, vol. 92, no. 5. American Physical Society (APS), Aug. 26, 2015. doi: 10.1103/physrevb.92.054110.

2H-$MoTe_2$

2H-$MoTe_2$ bulk crystals were purchased from HQ-Graphene. From the purchased crystals one was selected and fixed on a SEM specimen stub with the help of sticking carbon tape.

Bright field image

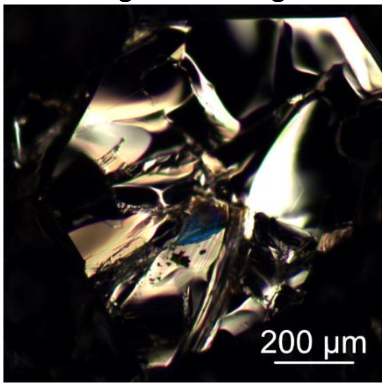

Dark field image

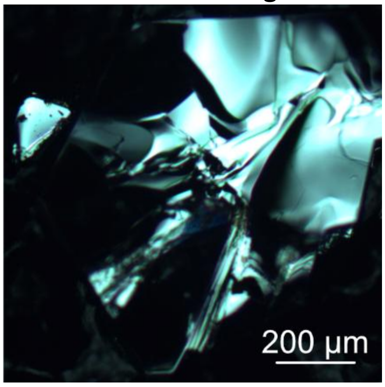

SE 30 keV

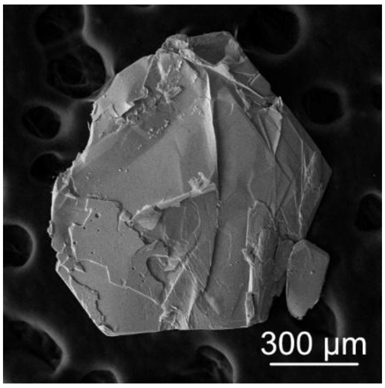

SE 1 keV

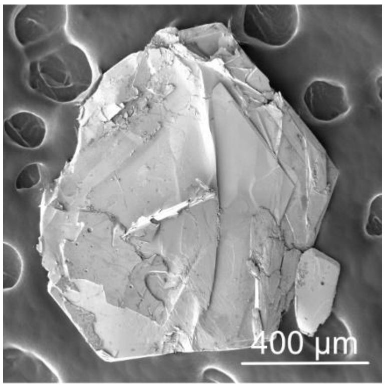

BSE 30 keV

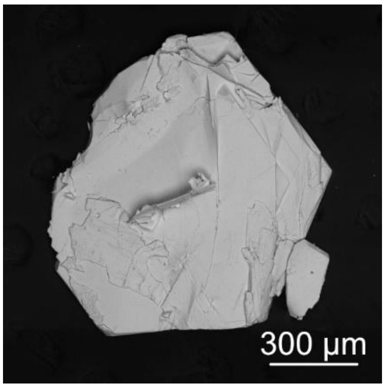

SE 1 keV (close up)

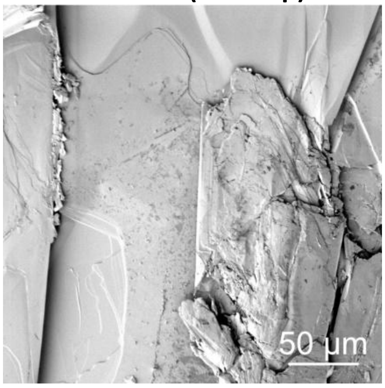

Monte Carlo simulations (CASINO)

d_e		$<d_{PE}>$ (d_{stdv})	
1 keV	30 keV	1 keV	30 keV
8.8 nm	81.5 nm	9.5 nm (3.6 nm)	1314 nm (489 nm)
$<d_{BSE}>$ (d_{stdv})		β (BSE/SE)	
2.6 nm (1.9 nm)	424 nm (253 nm)	0.362	0.396

Material parameters for simulations

ρ (g/cm³)	Z	Z_{eff}
7.7	Mo: 42 Te: 52	49.87

Raman spectrum

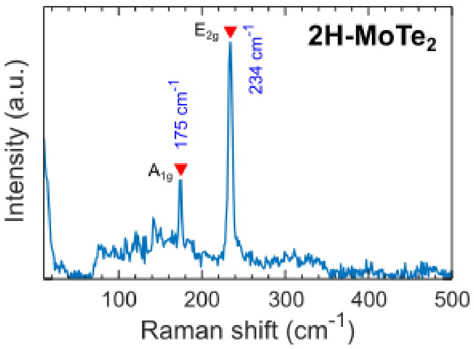

We observe Raman peaks at 175 cm^{-1} and 234 cm^{-1}, which correspond to the characteristic vibrational modes of 2H-$MoTe_2$. The peak at 175 cm^{-1} is attributed to the E2g mode, representing the in-plane vibration of molybdenum and tellurium atoms. The peak at 234 cm^{-1} corresponds to the A1g mode, which arises from the out-of-plane vibration of tellurium atoms [11].

[11] T. A. Empante et al., “Chemical Vapor Deposition Growth of Few-Layer MoTe2 in the 2H, 1T’, and 1T Phases: Tunable Properties of MoTe2 Films,” ACS Nano, vol. 11, no. 1. American Chemical Society (ACS), pp. 900–905, Jan. 03, 2017. doi: 10.1021/acsnano.6b07499.

Td-WTe_2

Td-WTe_2 bulk crystals were purchased from HQ-Graphene. From the purchased crystals one was selected and fixed on a SEM specimen stub with the help of sticking carbon tape.

Bright field image

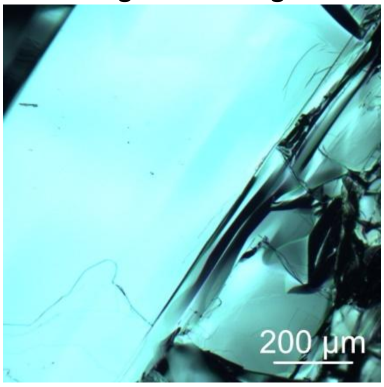

Dark field image

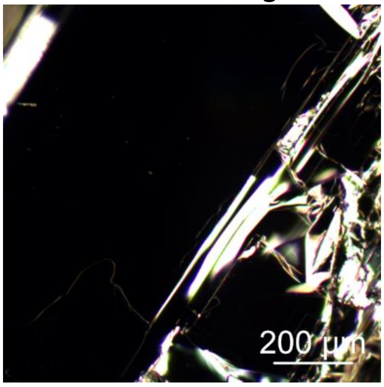

SE 30 keV

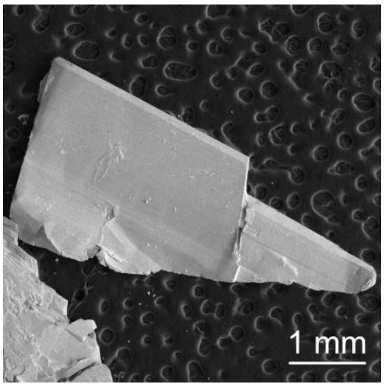

SE 1 keV

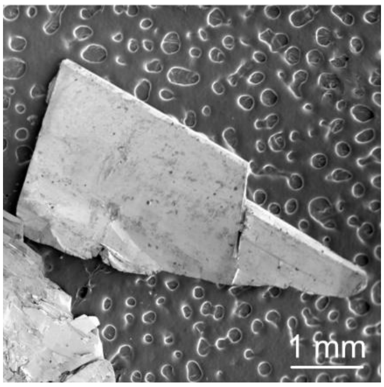

BSE 30 keV

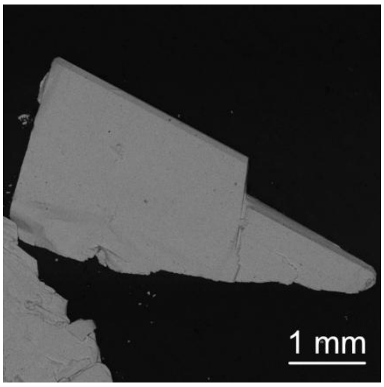

SE 1 keV (close up)

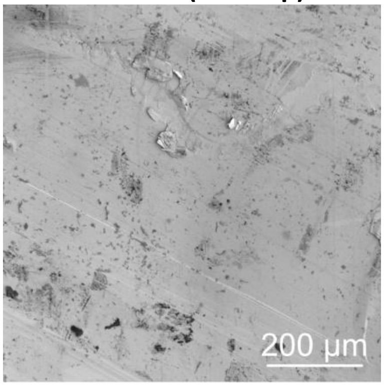

Monte Carlo simulations (CASINO)

d_e		$\langle d_{PE}\rangle$ (d_{stdv})	
1 keV	30 keV	1 keV	30 keV
8.6 nm	88.5 nm	9.1 nm (3.5 nm)	1078 nm (394 nm)
$\langle d_{BSE}\rangle$ (d_{stdv})		β (BSE/SE)	
2.5 nm (1.8 nm)	339 nm (211 nm)	0.378	0.435

Material parameters for simulations

ρ (g/cm^3)	Z	Z_{eff}
9.43	W: 74 Te: 52	59.29

Raman spectrum

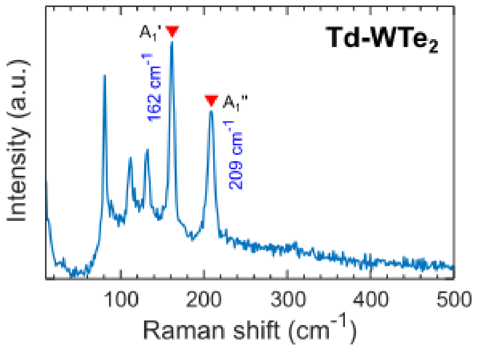

We observe Raman peaks at 162 cm^{-1} and 209 cm^{-1}, which correspond to the characteristic vibrational modes of Td-WTe_2. The peak at 162 cm^{-1} is attributed to an A_1 mode, representing the out-of-plane vibrations of tungsten and tellurium atoms. The peak at 209 cm^{-1} corresponds to another A_1 mode, which involves mixed in-plane and out-of-plane atomic displacements [12].

[12] M. K. Jana et al., "A combined experimental and theoretical study of the structural, electronic and vibrational properties of bulk and few-layer Td-WTe2," Journal of Physics: Condensed Matter, vol. 27, no. 28. IOP Publishing, p. 285401, Jun. 23, 2015. doi: 10.1088/0953-8984/27/28/285401.

1T-TiS_2

1T-TiS_2 bulk crystals were purchased from HQ-Graphene. From the purchased crystals one was selected and fixed on a SEM specimen stub with the help of sticking carbon tape.

Bright field image

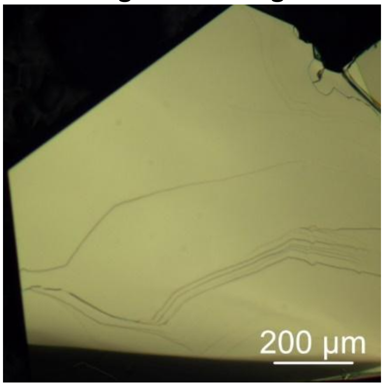

Dark field image

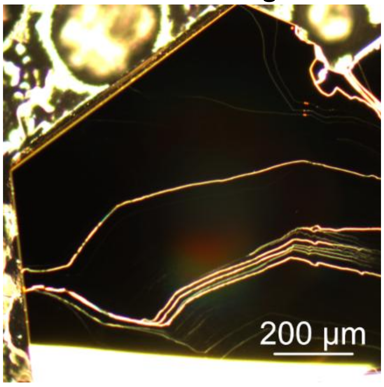

SE 30 keV

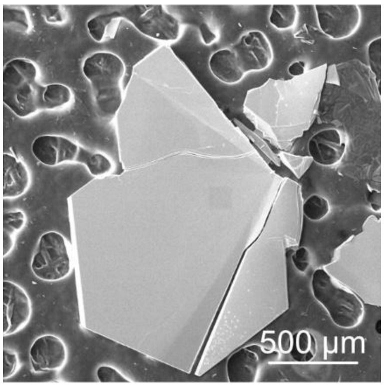

SE 1 keV

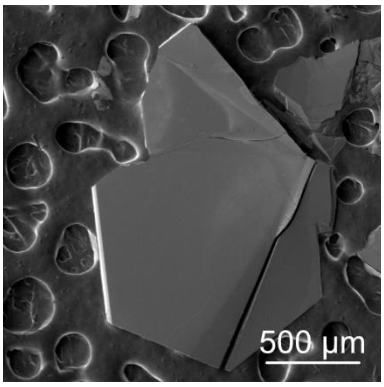

BSE 30 keV

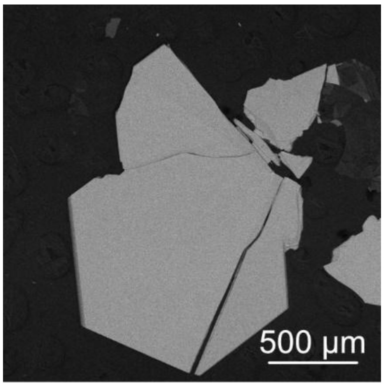

SE 1 keV (close up)

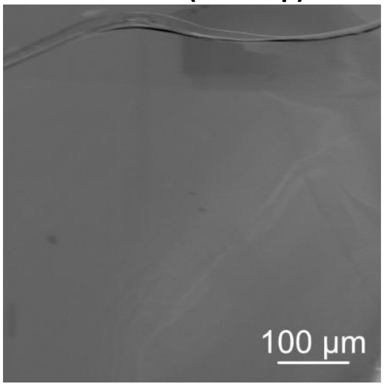

Monte Carlo simulations (CASINO)

d_e		$\langle d_{PE} \rangle$ (d_{stdv})	
1 keV	30 keV	1 keV	30 keV
13.8 nm	273 nm	12.7 nm (4.6 nm)	2948 nm (1049 nm)
$\langle d_{BSE} \rangle$ (d_{stdv})		β (BSE/SE)	
3.6 nm (2.4 nm)	1018 nm (526 nm)	0.227	0.190

Material parameters for simulations

ρ (g/cm^3)	Z	Z_{eff}
3.37	Ti: 22 S: 16	17.89

Raman spectrum

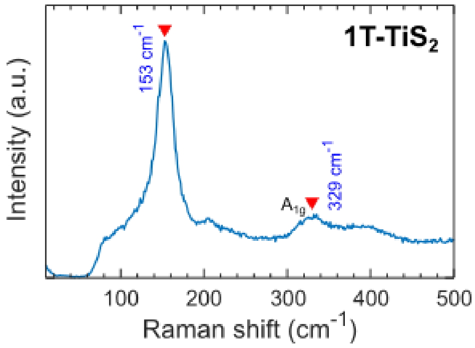

We observe Raman peaks at 153 cm^{-1} and 329 cm^{-1}, which correspond to the characteristic vibrational modes of 1T-TiS_2. The peak at 153 cm^{-1} is attributed to the Eg mode, representing the in-plane vibration of titanium and sulfur atoms. The peak at 329 cm^{-1} corresponds to the A1g mode, which arises from the out-of-plane vibration of sulfur atoms [13].

[13] R. Zhang et al., “Probing the photocurrent in two-dimensional titanium disulfide,” Nanotechnology, vol. 35, no. 1. IOP Publishing, p. 015708, Oct. 20, 2023. doi: 10.1088/1361-6528/ad0054.

2H-$NbSe_2$

2H-$NbSe_2$ bulk crystals were purchased from HQ-Graphene. From the purchased crystals one was selected and fixed on a SEM specimen stub with the help of sticking carbon tape.

Bright field image

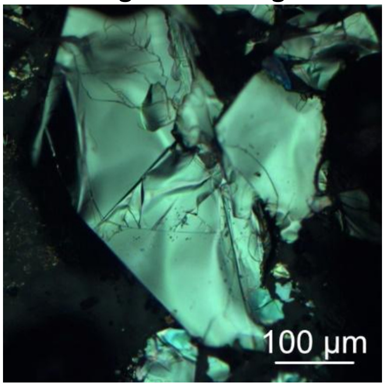

Dark field image

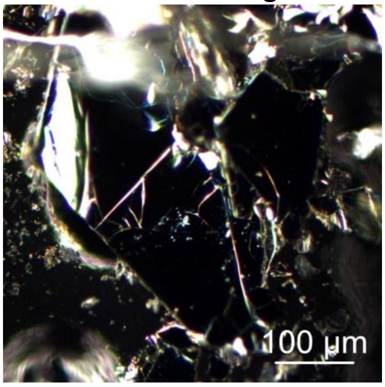

SE 30 keV

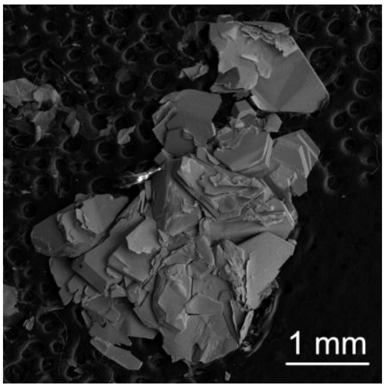

SE 1 keV

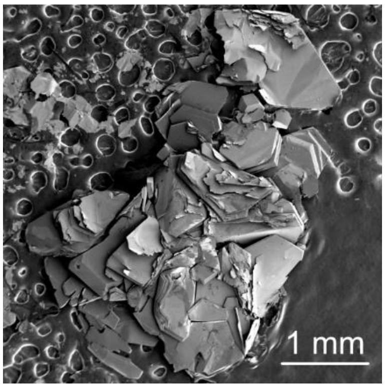

BSE 30 keV

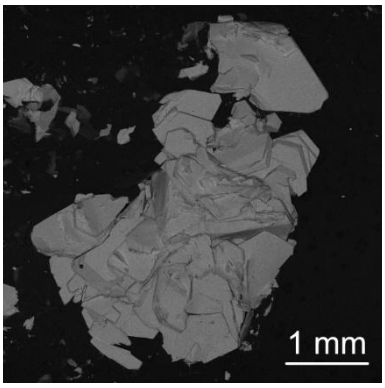

SE 1 keV (close up)

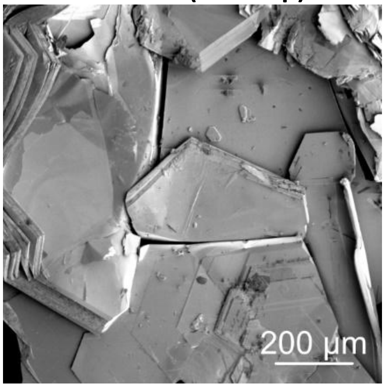

Monte Carlo simulations (CASINO)

d_e		$\langle d_{PE} \rangle$ (d_{stdv})	
1 keV	30 keV	1 keV	30 keV
9.9 nm	163 nm	9.9 nm (3.8 nm)	1568 nm (580 nm)
$\langle d_{BSE} \rangle$ (d_{stdv})		β (BSE/SE)	
2.8 nm (2.0 nm)	536 nm (299 nm)	0.330	0.331

Material parameters for simulations

ρ (g/cm³)	Z	Z_{eff}
6.3	Nb: 41 Se: 34	36.37

Raman spectrum

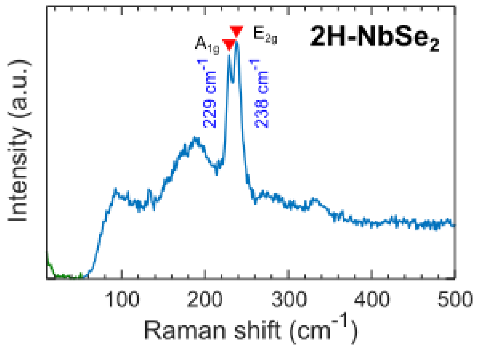

We observe Raman peaks at 229 cm^{-1} and 238 cm^{-1}, which correspond to the characteristic vibrational modes of 2H-$NbSe_2$. The peak at 229 cm^{-1} is attributed to the E_{2g} mode, representing the in-plane vibration of niobium and selenium atoms. The peak at 238 cm^{-1} corresponds to the A_{1g} mode, which arises from the out-of-plane vibration of selenium atoms [14].

[14] X. Xi et al., "Strongly enhanced charge-density-wave order in monolayer NbSe2," Nature Nanotechnology, vol. 10, no. 9. Springer Science and Business Media LLC, pp. 765–769, Jul. 20, 2015. doi: 10.1038/nnano.2015.143.

2H-$TaSe_2$

2H-$TaSe_2$ bulk crystals were purchased from HQ-Graphene. From the purchased crystals one was selected and fixed on a SEM specimen stub with the help of sticking carbon tape.

Bright field image

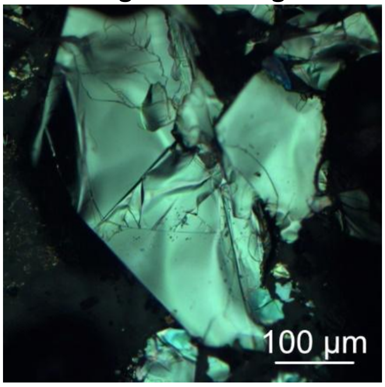

Dark field image

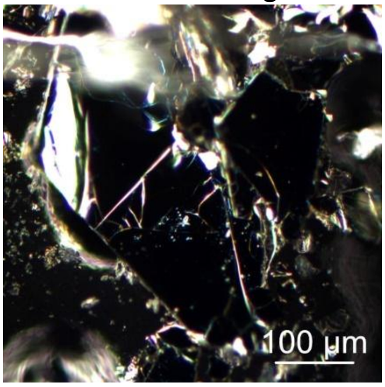

SE 30 keV

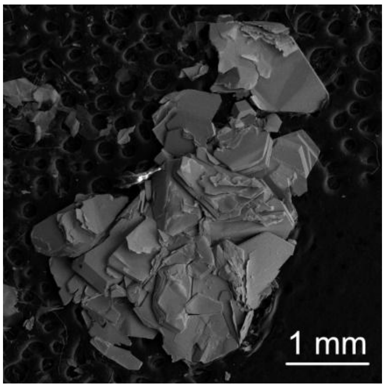

SE 1 keV

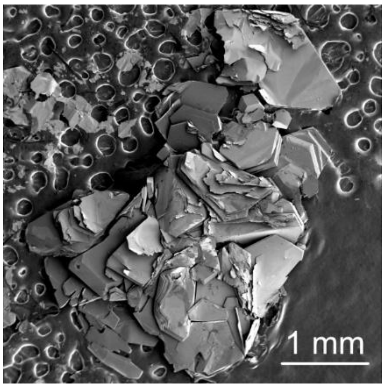

BSE 30 keV

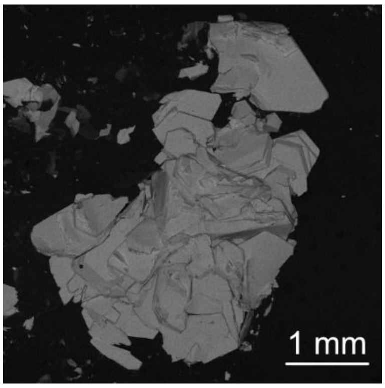

SE 1 keV (close up)

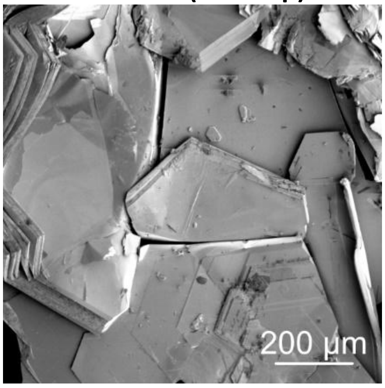

Monte Carlo simulations (CASINO)

d_e		$<d_{PE}>$ (d_{stdv})	
1 keV	30 keV	1 keV	30 keV
11.4 nm	121.2 nm	11.7 nm (4.5 nm)	1493 nm (549 nm)
$<d_{BSE}>$ (d_{stdv})		β (BSE/SE)	
3.2 nm (2.3 nm)	476 nm (292 nm)	0.352	0.404

Material parameters for simulations

ρ (g/cm^3)	Z	Z_{eff}
6.7	Ta: 73 Se: 34	46.44

Raman spectrum

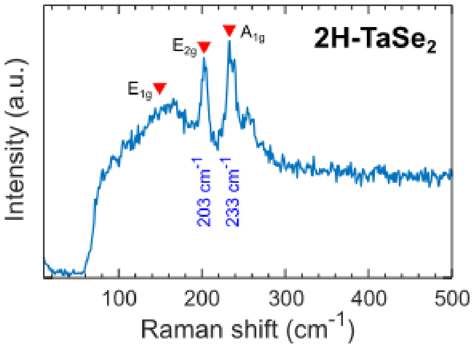

We observe Raman peaks at 203 cm^{-1} and 233 cm^{-1}, which correspond to the characteristic vibrational modes of 2H-$TaSe_2$. The peak at 203 cm^{-1} is attributed to the E_2g mode, representing the in-plane vibration of tantalum and selenium atoms. The peak at 233 cm^{-1} corresponds to the A_1g mode, which arises from the out-of-plane vibration of selenium atoms [15].

[15] M. Mahajan, S. Kallatt, M. Dandu, N. Sharma, S. Gupta, and K. Majumdar, "Light emission from the layered metal 2H-TaSe2 and its potential applications," Communications Physics, vol. 2, no. 1. Springer Science and Business Media LLC, Jul. 31, 2019. doi: 10.1038/s42005-019-0190-0.

cBN

cBN micro crystals were purchased from Thermo Fischer Scientific. The purchased crystals where dispersed on a sticking carbon tape.

Bright field image

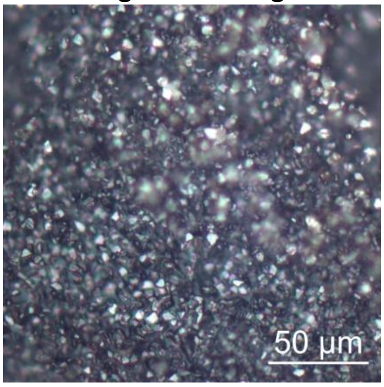

Dark field image

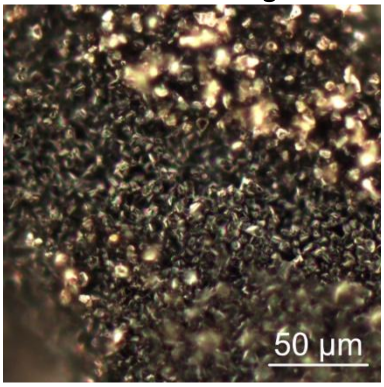

SE 30 keV

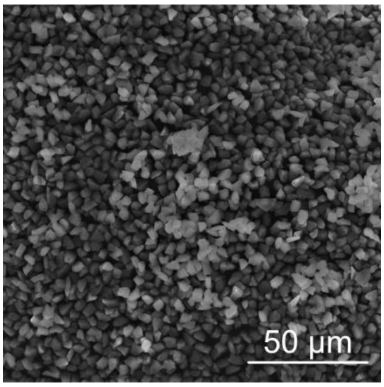

SE 1 keV

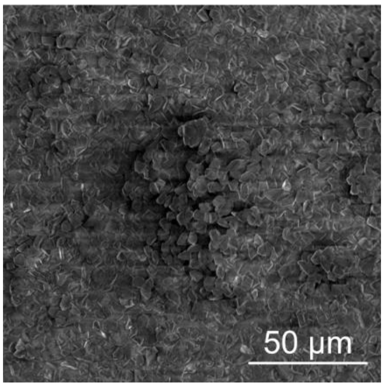

BSE 30 keV

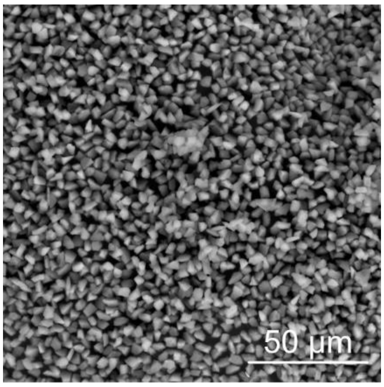

SE 30 keV (close up)

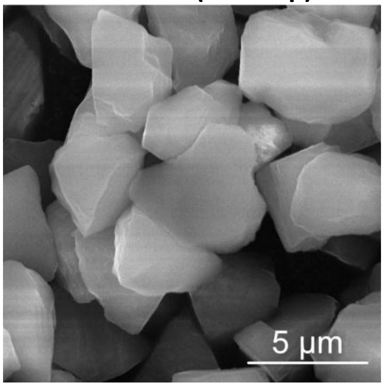

Monte Carlo simulations (CASINO)

d_e		$\langle d_{PE} \rangle$ (d_{stdv})	
1 keV	30 keV	1 keV	30 keV
11.7 nm	382 nm	10.0 nm (3.1 nm)	3558 nm (934 nm)
$\langle d_{BSE} \rangle$ (d_{stdv})		β (BSE/SE)	
2.6 nm (1.6 nm)	1027 nm (559 nm)	0.076	0.045

Material parameters for simulations

ρ (g/cm^3)	Z	Z_{eff}
3.45	B: 5 N: 7	6.04

Raman spectrum

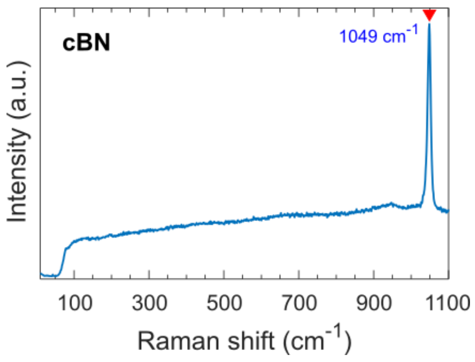

The primary first-order Raman-active mode is around 1049 cm^{-1}, which is the transverse optical (TO) phonon [16].

[16] F. Datchi and B. Canny, "Raman spectrum of cubic boron nitride at high pressure and temperature," Physical Review B, vol. 69, no. 14. American Physical Society (APS), Apr. 06, 2004. doi: 10.1103/physrevb.69.144106.

hBN (micro powder)

hBN micro crystals were purchased from 2D-Semiconductors. The purchased crystals were dispersed on a sticking carbon tape.

Bright field image

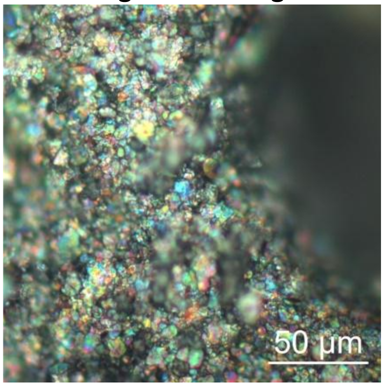

Dark field image

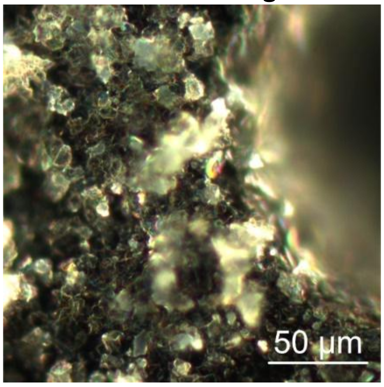

SE 10 keV

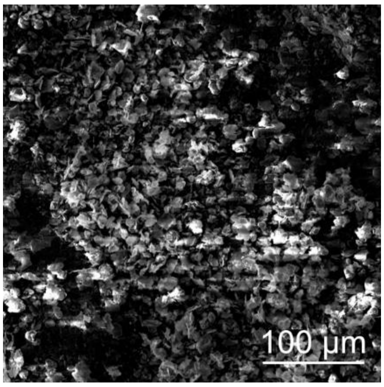

SE 1 keV

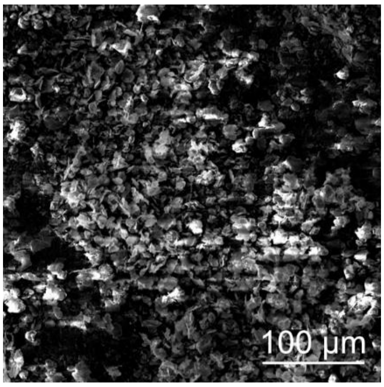

BSE 10 keV

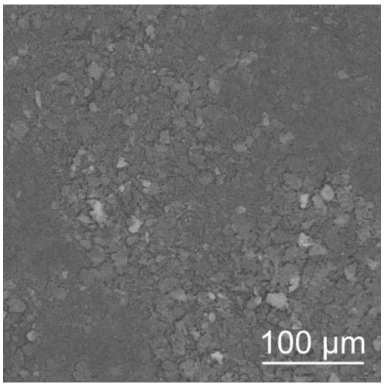

SE 10 keV (close up)

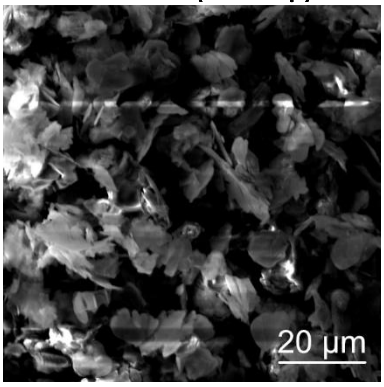

Monte Carlo simulations (CASINO)

d_e		$\langle d_{PE} \rangle$ (d_{stdv})	
1 keV	30 keV	1 keV	30 keV
19.1 nm	556 nm	16.5 nm (5.1 nm)	5843 nm (1631 nm)
$\langle d_{BSE} \rangle$ (d_{stdv})		β (BSE/SE)	
4.3 nm (2.6 nm)	1688 nm (921 nm)	0.076	0.045

Material parameters for simulations

ρ (g/cm^3)	Z	Z_{eff}
2.10	B: 5 N: 7	6.04

Raman spectrum

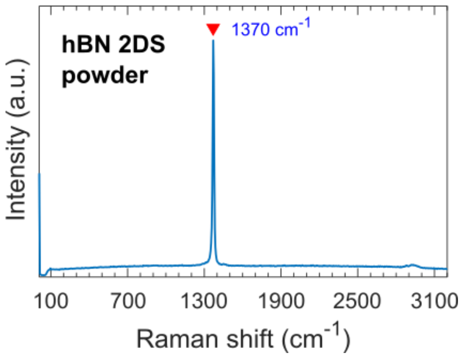

A prominent Raman-active E_{2g} mode is at 1370 cm^{-1}, corresponding to the in-plane vibrational stretching of boron and nitrogen atoms in its hexagonal lattice structure [17].

[17] L. H. Li and Y. Chen, "Atomically Thin Boron Nitride: Unique Properties and Applications," Advanced Functional Materials, vol. 26, no. 16. Wiley, pp. 2594–2608, Feb. 16, 2016. doi: 10.1002/adfm.201504606.

hBN (Crystal 2D Semiconductors)

hBN crystals were purchased from 2D Semiconductors. From the purchased crystals one was selected and fixed on a SEM specimen stub with the help of sticking carbon tape.

Bright field image

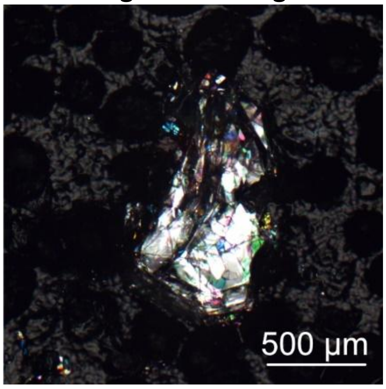

Dark field image

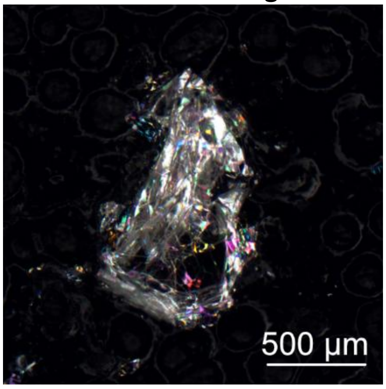

SE 10 keV

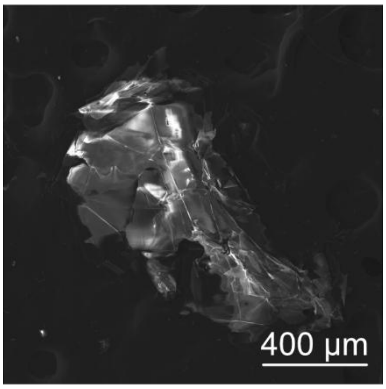

SE 1 keV

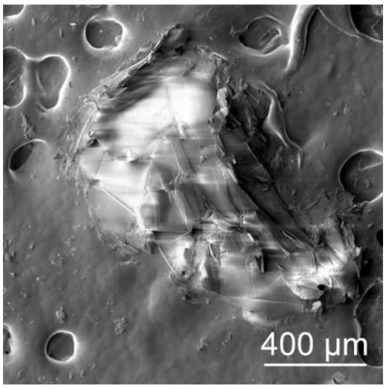

BSE 10 keV

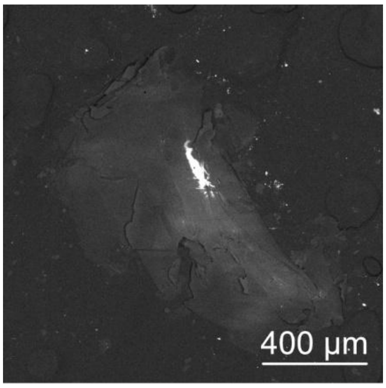

SE 1 keV (close up)

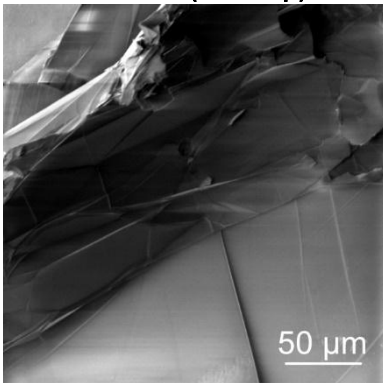

Monte Carlo simulations (CASINO)

d_e		$<d_{PE}>$ (d_{stdv})	
1 keV	30 keV	1 keV	30 keV
19.1 nm	556 nm	16.5 nm (5.1 nm)	5843 nm (1631 nm)
$<d_{BSE}>$ (d_{stdv})		β (BSE/SE)	
4.3 nm (2.6 nm)	1688 nm (921 nm)	0.076	0.045

Material parameters for simulations

ρ (g/cm³)	Z	Z_{eff}
2.10	B: 5 N: 7	6.04

Raman spectrum

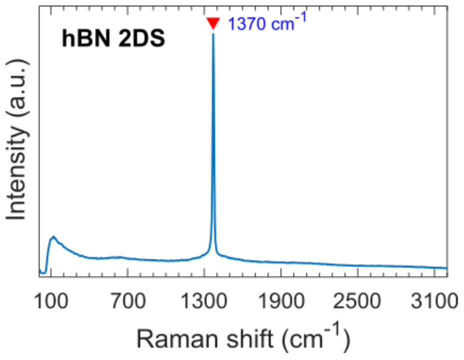

A prominent Raman-active E_{2g} mode is at 1370 cm^{-1}, corresponding to the in-plane vibrational stretching of boron and nitrogen atoms in its hexagonal lattice structure [17].

hBN (Crystal HQ Graphene)

hBN crystals were purchased from HQ-Graphene. From the purchased crystals one was selected and fixed on a SEM specimen stub with the help of sticking carbon tape.

Bright field image

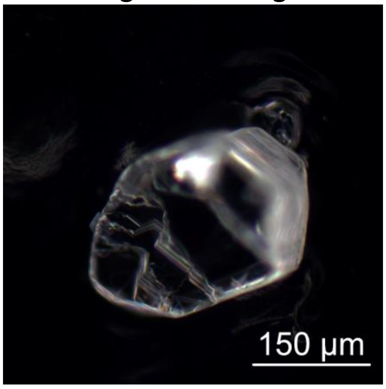

Dark field image

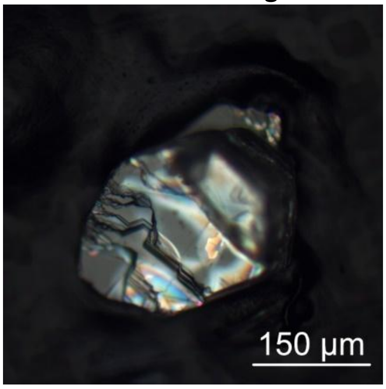

SE 10 keV

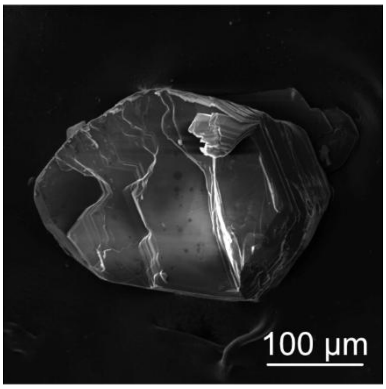

SE 1 keV

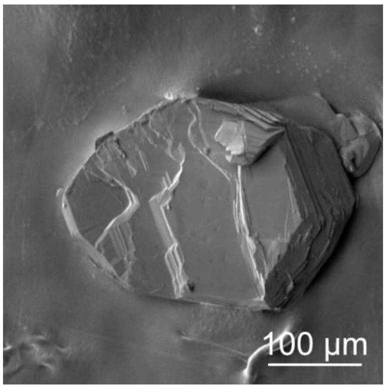

BSE 10 keV

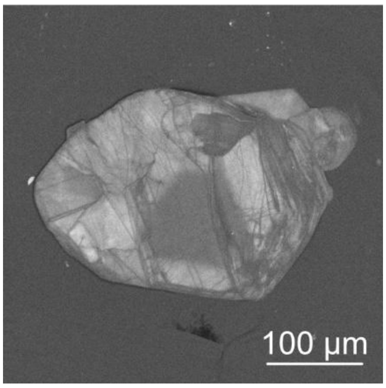

SE 1 keV (close up)

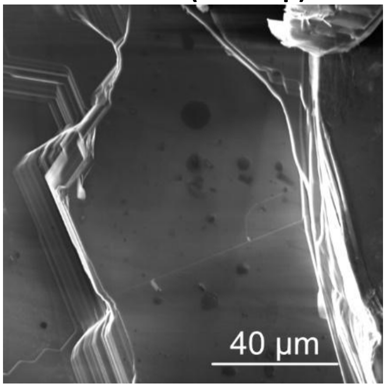

Monte Carlo simulations (CASINO)

d_e		$\langle d_{PE} \rangle$ (d_{stdv})	
1 keV	30 keV	1 keV	30 keV
19.1 nm	556 nm	16.5 nm (5.1 nm)	5843 nm (1631 nm)
$\langle d_{BSE} \rangle$ (d_{stdv})		β (BSE/SE)	
4.3 nm (2.6 nm)	1688 nm (921 nm)	0.076	0.045

Material parameters for simulations

ρ (g/cm^3)	Z	Z_{eff}
2.10	B: 5 N: 7	6.04

Raman spectrum

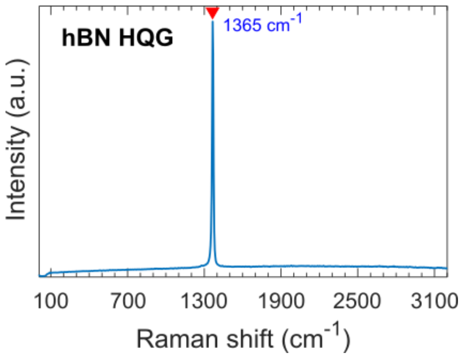

A prominent Raman-active E_{2g} mode is at 1365 cm^{-1}, corresponding to the in-plane vibrational stretching of boron and nitrogen atoms in its hexagonal lattice structure [17].

Diamond (micro crystals)

Diamond micro crystals were purchased from Thermo Fischer Scientific. The purchased crystals were dispersed on a sticking carbon tape.

Bright field image

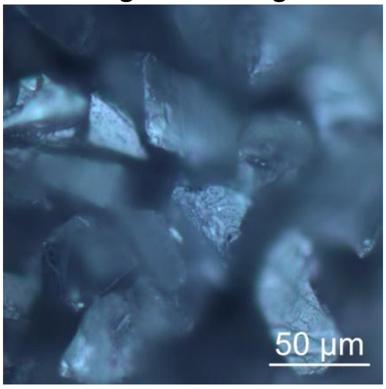

Dark field image

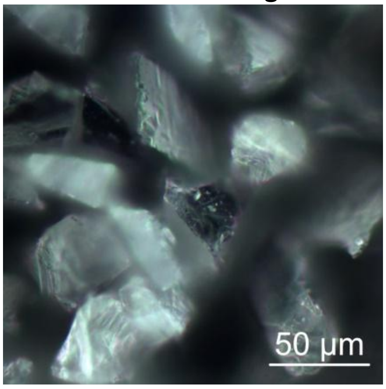

SE 30 keV

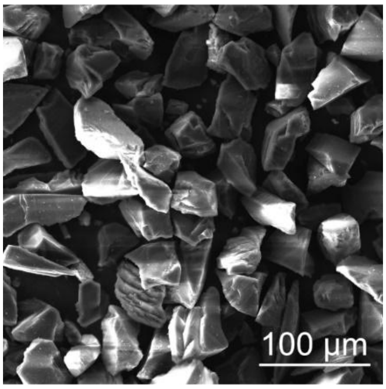

SE 1 keV

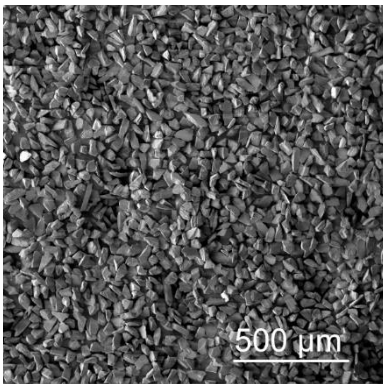

BSE 30 keV

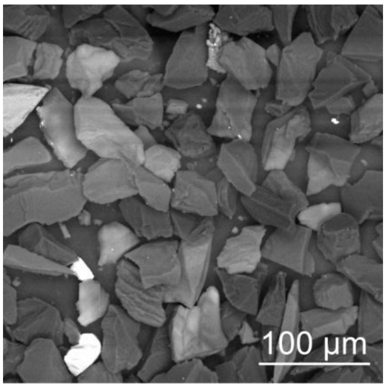

SE 1 keV (close up)

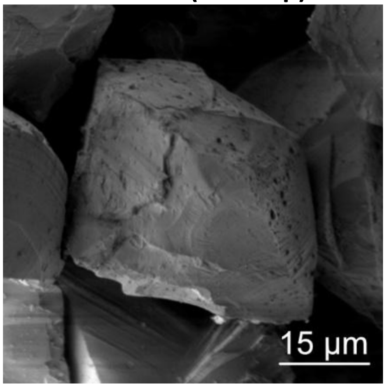

Monte Carlo simulations (CASINO)

d_e		$<d_{PE}>$ (d_{stdv})	
1 keV	30 keV	1 keV	30 keV
11.0 nm	333 nm	9.5 nm (2.9 nm)	3408 nm (939 nm)
$<d_{BSE}>$ (d_{stdv})		β (BSE/SE)	
2.5 nm (1.5 nm)	958 nm (521 nm)	0.075	0.041

Material parameters for simulations

ρ (g/cm^3)	Z	Z_{eff}
3.51	6	6

Raman spectrum

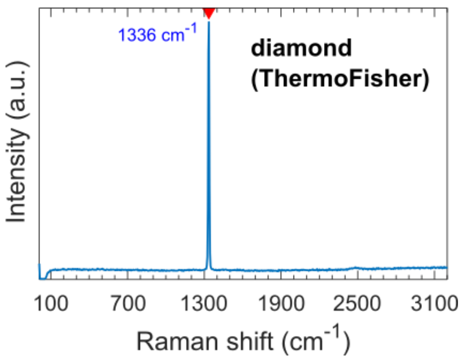

We observe a Raman peak at 1336 cm^{-1}, corresponding to the first order scattering of the zone-center optical phonon, which is characteristic of its crystalline structure [18].

[18] Z. Li, L. Deng, I. A. Kinloch, and R. J. Young, "Raman spectroscopy of carbon materials and their composites: Graphene, nanotubes and fibres," Progress in Materials Science, vol. 135. Elsevier BV, p. 101089, Jun. 2023. doi: 10.1016/j.pmatsci.2023.101089.

Diamond (nano crystals with NV centers)

NV center nano diamonds were purchased from ADAMAS Nanotechnologies. The purchased crystals were diluted in water and drop casted on a silicon substrate.

SE 1 keV

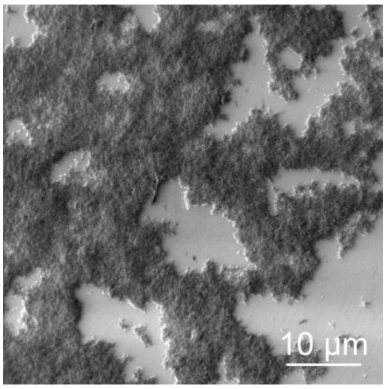

SE 10 keV (close up)

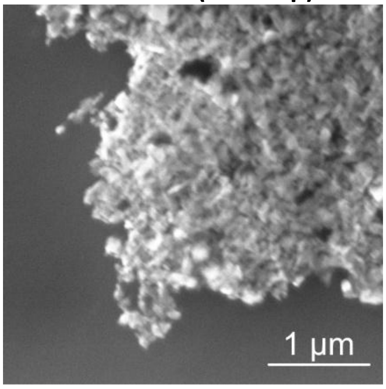

Raman spectrum

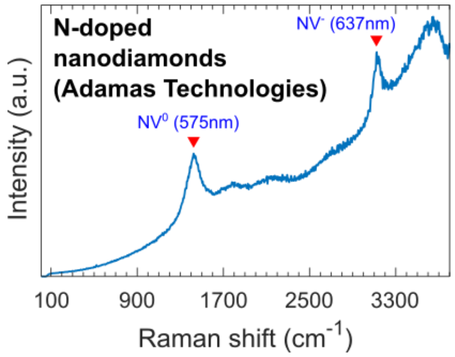

In N-doped nanodiamonds, we observe characteristic spectral features associated with nitrogen-vacancy (NV) color centers, specifically the NV^0 center with a zero-phonon line (ZPL) at 575 nm and the NV^- center with a ZPL at 637 nm. The spectrum reveals strong photoluminescence, which originates from these color centers and extends across a broad wavelength range. This intense PL emission overlaps and obscures the Raman signal.

Diamond (nano crystals with SiV centers)

SiV center nano diamonds were purchased from ADAMAS Nanotechnologies. The purchased crystals were diluted in water and drop casted on a silicon substrate.

SE 30 keV

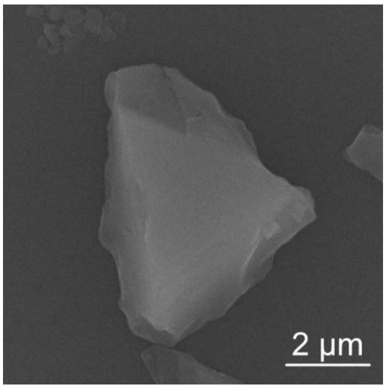

SE 1 keV (Overview)

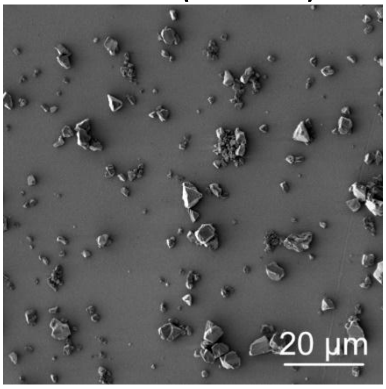

BSE 30 keV

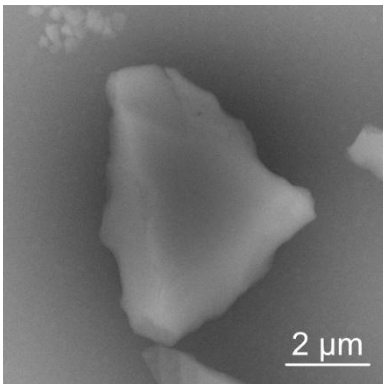

SE 1 keV

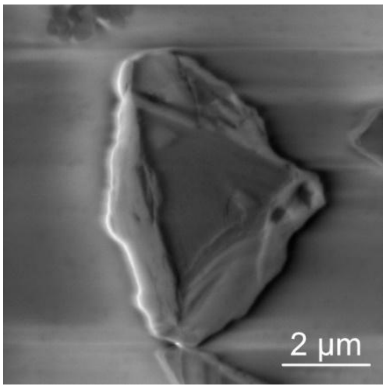

Raman spectrum

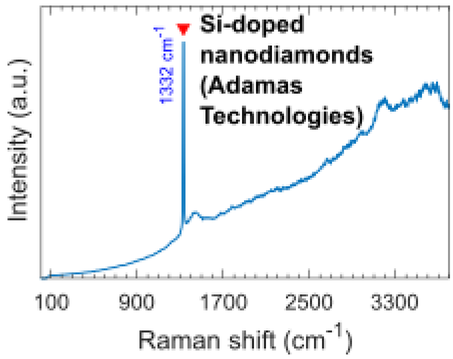

In Si-doped nanodiamonds, we observe a Raman peak at 1336 cm^{-1}, corresponding to the first-order diamond Raman line [18]. Additionally, spectral features associated with nitrogen-vacancy (NV) color centers are present, including the NV^0 zero-phonon line (ZPL) at 575 nm and the NV^- ZPL at 637 nm. The SiV ZPL at 737 nm lies outside the detectable spectral range. The spectrum exhibits strong photoluminescence (PL) from these color centers, extending across a broad wavelength range. This intense PL emission overlaps with and obscures the Raman signal.

Graphite

Natural graphite crystals were purchased from HQ-Graphene. The selected crystal was fixed on a SEM specimen stub with the help of sticking carbon tape.

Bright field image

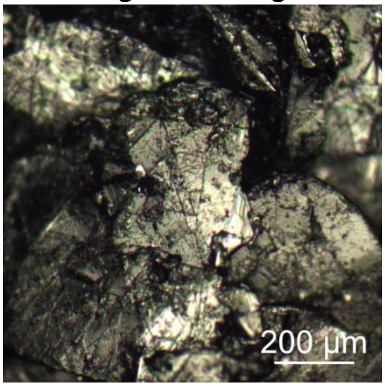

Dark field image

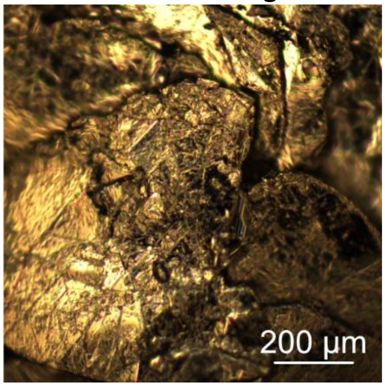

SE 30 keV

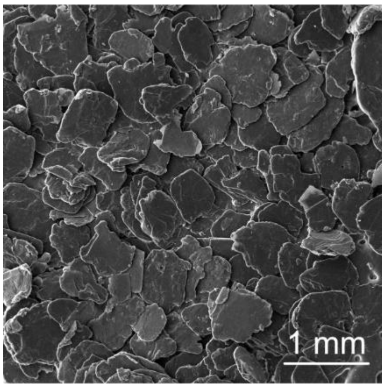

SE 1 keV

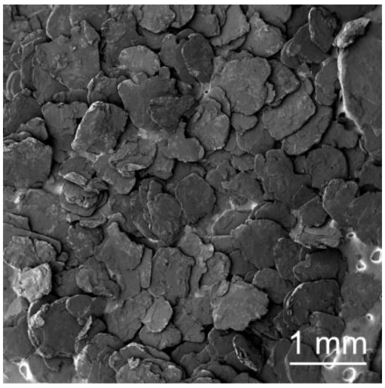

BSE 30 keV

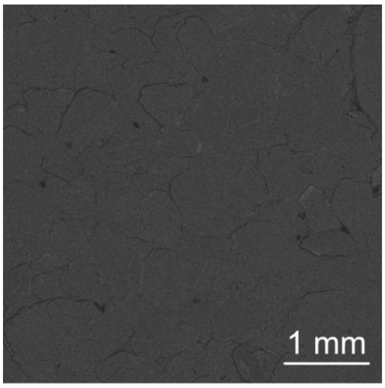

SE 1 keV (close up)

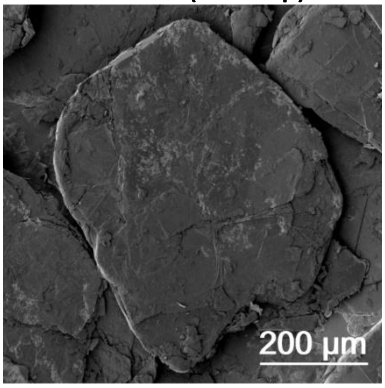

Monte Carlo simulations (CASINO)

d_e		$\langle d_{PE} \rangle$ (d_{stdv})	
1 keV	30 keV	1 keV	30 keV
17.5 nm	549 nm	15.2 nm (4.7 nm)	5437 nm (1496 nm)
$\langle d_{BSE} \rangle$ (d_{stdv})		β (BSE/SE)	
4.0 nm (2.4 nm)	1526 nm (835 nm)	0.075	0.042

Material parameters for simulations

ρ (g/cm^3)	Z	Z_{eff}
2.2	6	6

Raman spectrum

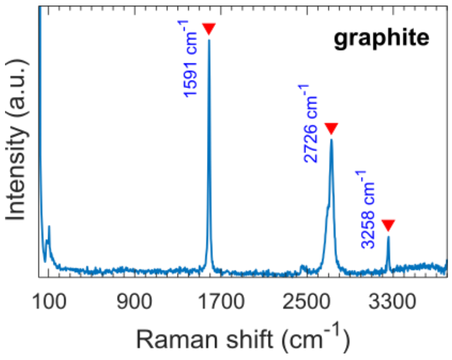

We observe Raman peaks at 1591 cm^{-1}, 2726 cm^{-1}, and 3258 cm^{-1}, which correspond to key vibrational modes in graphite. The 1591 cm^{-1} peak represents the G-band, associated with the E_{2g} phonon mode, originating from in-plane vibrations of sp^2-bonded carbon atoms. The 2726 cm^{-1} peak corresponds to the 2D-band (G' band), which is a second-order two-phonon scattering process and is highly sensitive to stacking order and number of layers. The 3258 cm^{-1} peak is a higher-order combination mode involving overtones of the D and G bands, commonly observed in defective or multilayered graphitic structures [18].

Quartz (SiO_2)

A quartz substrate was purchased from Sigma-Aldrich. The purchased substrate was fixed on a SEM specimen stub with the help of sticking carbon tape.

Bright field image

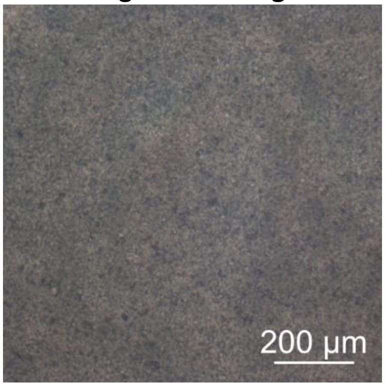

Dark field image

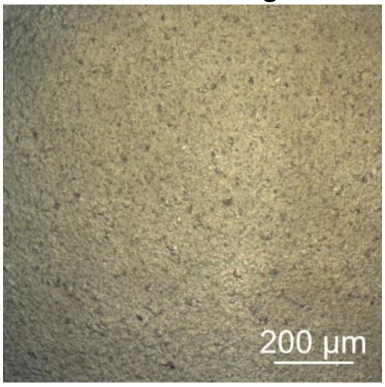

SE 10 keV

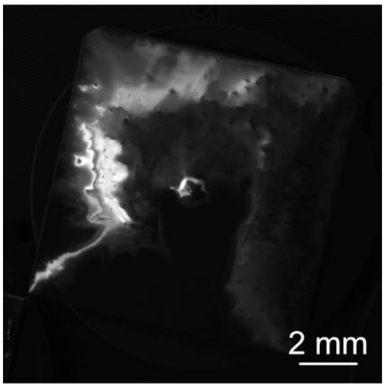

SE 1 keV

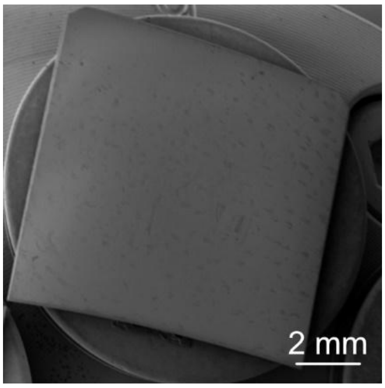

BSE 10 keV

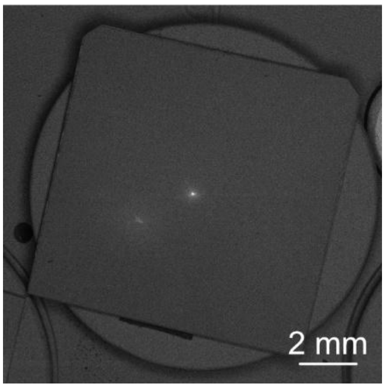

SE 1 keV (close up)

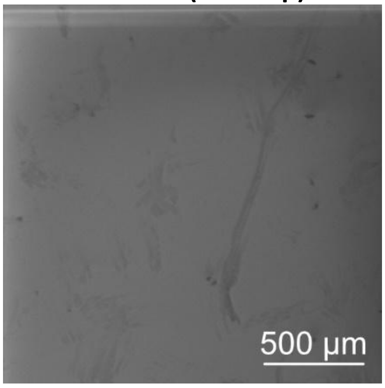

Monte Carlo simulations (CASINO)

d_e		<d_{PE}> (d_{stdv})	
1 keV	30 keV	1 keV	30 keV
18.1 nm	487 nm	15.8 nm (5.4 nm)	4593 nm (1498 nm)
<d_{BSE}> (d_{stdv})		β (BSE/SE)	
4.3 nm (2.8 nm)	1495 nm (769 nm)	0.144	0.099

Material parameters for simulations

ρ (g/cm^3)	Z	Z_{eff}
2.65	Si: 16 O: 8	10

Raman spectrum

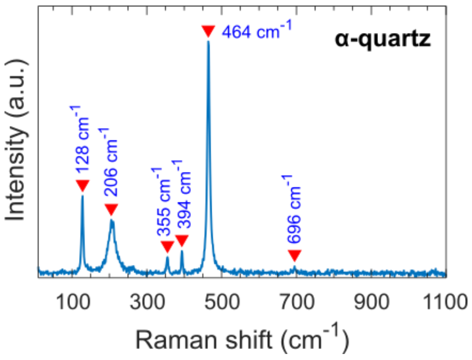

α-quartz is the most stable form of crystalline silica (SiO_2) at ambient conditions, it exhibits a Raman spectrum with several prominent peaks corresponding to its trigonal crystal structure. Notable Raman shifts include modes at approximately 128 cm^{-1}, 206 cm^{-1}, 355 cm^{-1}, 394 cm^{-1}, 464 cm^{-1}, and 696 cm^{-1}. These peaks are associated with various vibrational modes within the crystal lattice, such as Si–O–Si bending and stretching motions [19].

[19] D. Krishnamurti, "The raman spectrum of quartz and its interpretation," Proceedings of the Indian Academy of Sciences - Section A, vol. 47, no. 5. Springer Science and Business Media LLC, pp. 276–291, May 1958. doi: 10.1007/bf03052811.

100 nm SiO_2 on silicon

We obtained 100 nm SiO_2 on silicon wafers from Nanografi (thermally evaporated on crystalline Si), cleaved them into smaller pieces, and mounted one piece on an SEM stub using conductive carbon tape.

Bright field image

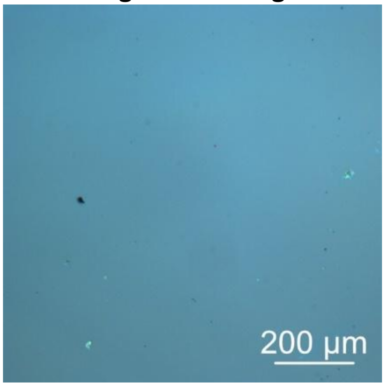

Dark field image

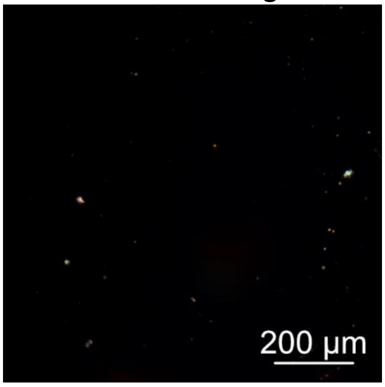

SE 10 keV

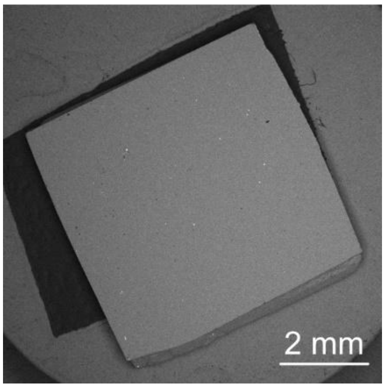

SE 1 keV

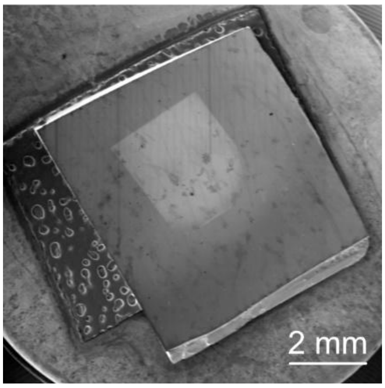

BSE 10 keV

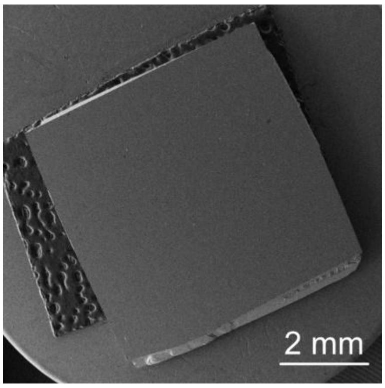

SE 1 keV (close up)

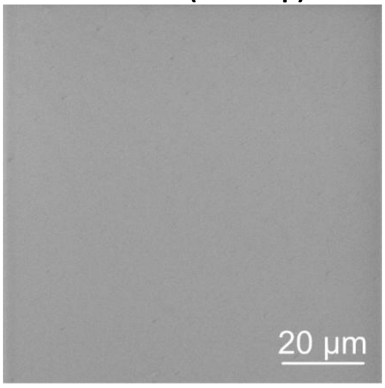

Raman spectrum

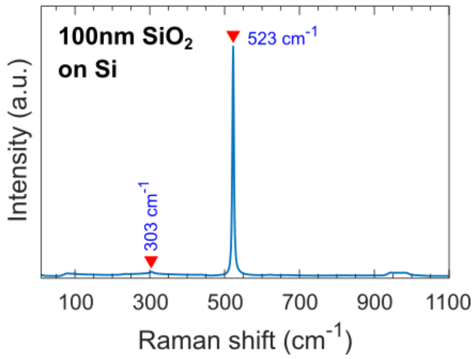

We see dominant response of Si in the Raman spectrum of a 100 nm SiO_2 layer on Si sample.
The Raman response of a 100 nm SiO_2 layer on Si is not observed due to its very low thickness, which results in weak scattering that is overshadowed by the strong signal from the Si substrate [5].

Menzel coverslip

Menzel coverslips were purchased from Thermo Fischer Scientific. The selected coverslip was fixed on a SEM specimen stub with the help of sticking carbon tape. Here we do not provide Monte Carlo simulation parameters due to the unknow chemical composition of the Menzel coverslip.

Bright field image

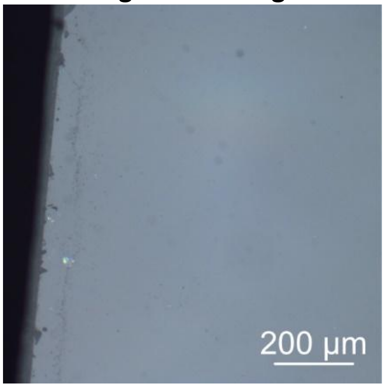

Dark field image

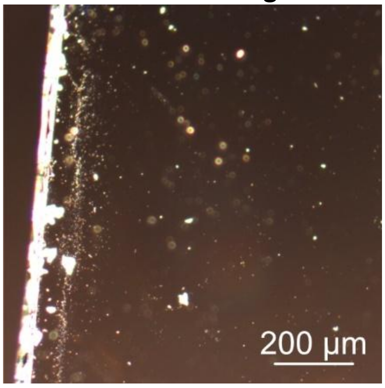

Raman spectrum

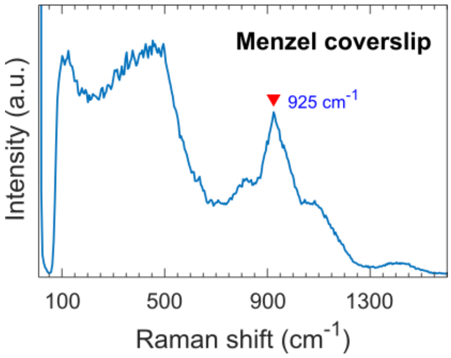

SE 10 keV

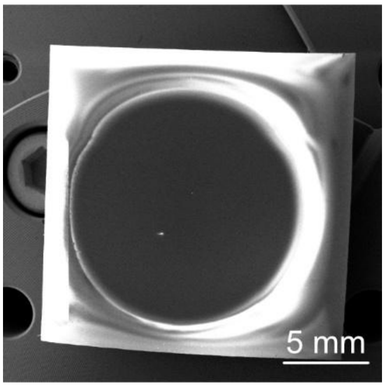

SE 1 keV

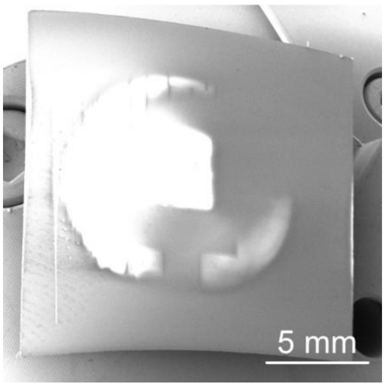

BSE 10 keV

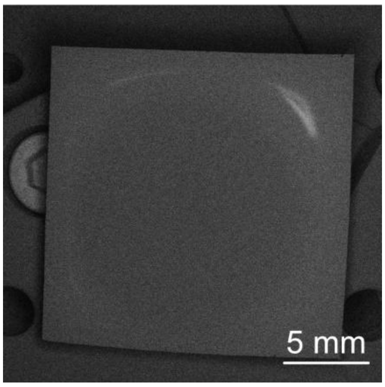

SE 1 keV (close up)

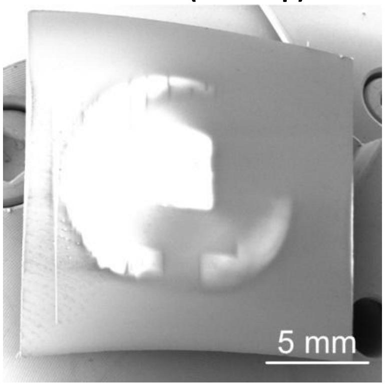

Menzel glass coverslips, commonly used in microscopy, are composed of amorphous silica. Their Raman spectra typically feature broad bands due to the disordered structure of the glass matrix. A notable broad band is often observed around 300-600 cm^{-1}, corresponding to Si–O–Si bending vibrations. Additionally, a less distinct peak near 925 cm^{-1} can be attributed to Si–O stretching modes. The broad nature of these peaks arises from the lack of long-range order in the glass structure, leading to a distribution of vibrational environments [20].

[20] R. B. Laughlin and J. D. Joannopoulos, “Phonons in amorphous silica,” Physical Review B, vol. 16, no. 6. American Physical Society (APS), pp. 2942–2952, Sep. 15, 1977. doi: 10.1103/physrevb.16.2942.

BK7

BK7 was purchased in the form of BK7 wafers. The BK7 wafer was cleaved into sized pieces. The selected piece was fixed on a SEM specimen stub with the help of sticking carbon tape. Here we do not provide Monte Carlo simulation parameters due to the unknow chemical composition of the BK7 wafer.

Bright field image

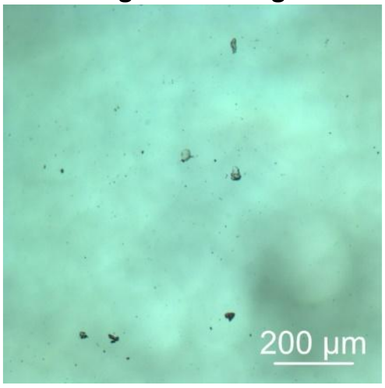

Dark field image

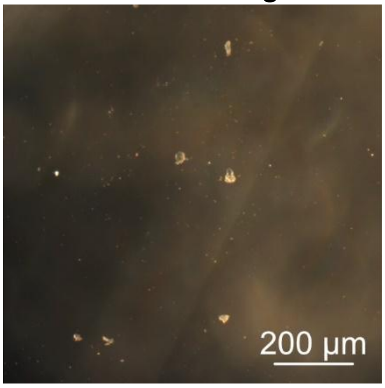

SE 10 keV

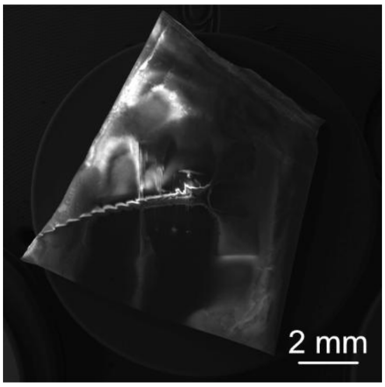

SE 1 keV

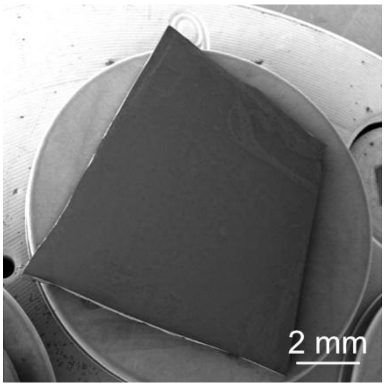

BSE 10 keV

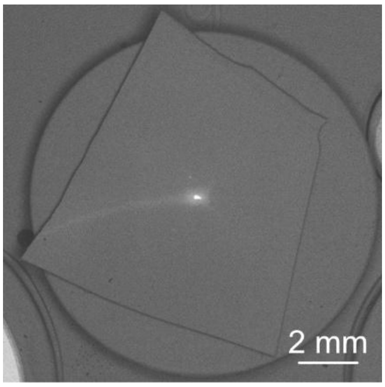

SE 1 keV (close up)

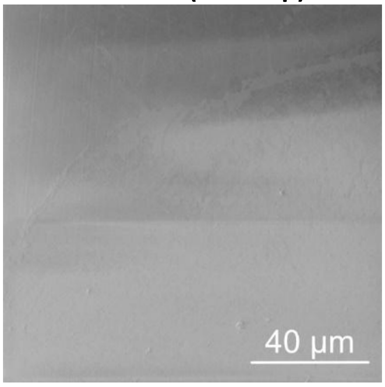

Raman spectrum

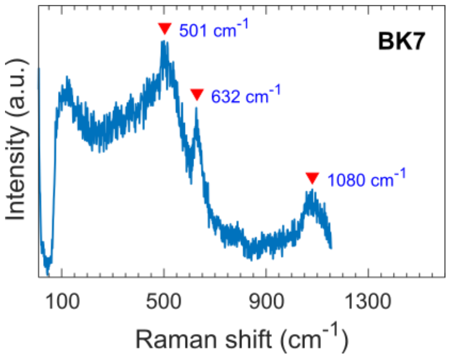

BK7 glass, a borosilicate material widely used in optical applications, exhibits characteristic Raman spectral features corresponding to its physical structure. Prominent peaks are observed at approximately 501 cm^{-1}, 632 cm^{-1}, and 1080 cm^{-1}. The 501 cm^{-1} peak is attributed to the bending vibrations of Si–O–Si bonds, while the 632 cm^{-1} band corresponds to danburite-like structural units within the glass matrix. The 1080 cm^{-1} peak is associated with the stretching vibrations of SiO_4 tetrahedra [21].

[21] J. Wu et al., “Raman spectra study on modifications of BK7 glass induced by 1030-nm and 515-nm femtosecond laser,” Results in Physics, vol. 21. Elsevier BV, p. 103814, Feb. 2021. doi: 10.1016/j.rinp.2021.103814.

Mica

Mica substrates were purchased from Thermo Fischer Scientific. The chemical stoichiometry is $KO_2Al_2O_3SiO_2$. The selected MICA substrate was fixed on a SEM specimen stub with the help of sticking carbon tape. Here we do not provide Monte Carlo simulation parameters due to the complex chemical composition of the mica.

Bright field image

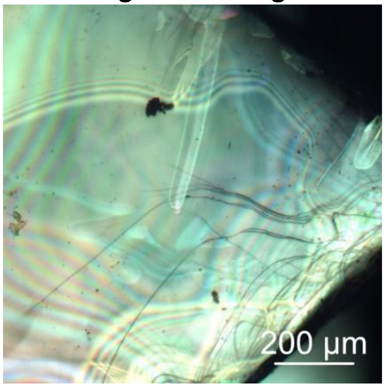

Dark field image

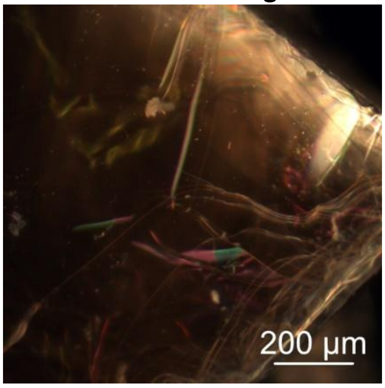

SE 10 keV

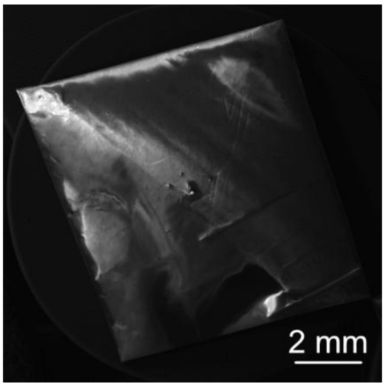

SE 1 keV

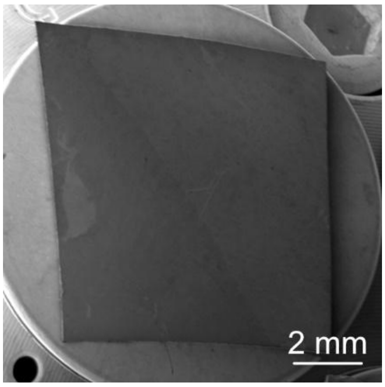

BSE 10 keV

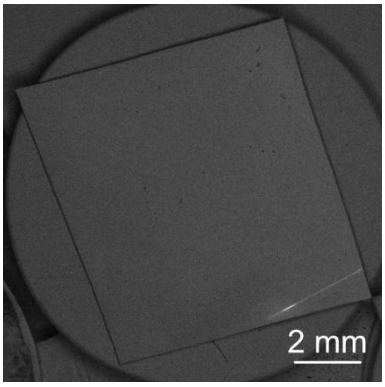

SE 1 keV (close up)

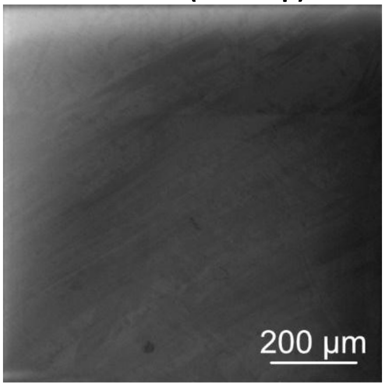

Raman spectrum

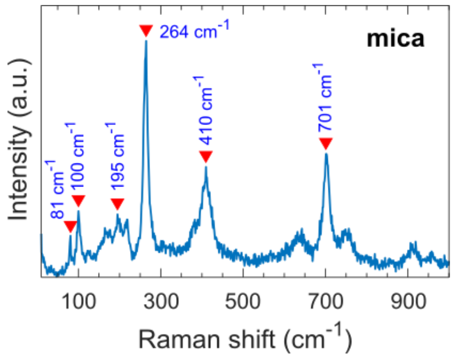

Mica minerals exhibit characteristic Raman spectra with distinct vibrational modes corresponding to their layered silicate structures. In the low-frequency region (~60–300 cm^{-1}), peaks are typically associated with lattice vibrations and interlayer interactions. Mid-frequency bands (~500–700 cm^{-1}) are attributed to Si–O–Si and Si–O–Al bending modes within the tetrahedral sheets. High-frequency features (~900–1200 cm^{-1}) correspond to Si–O stretching vibrations [22].

[22] A. Tlili, D. C. Smith, J.-M. Beny, and H. Boyer, “A Raman microprobe study of natural micas,” Mineralogical Magazine, vol. 53, no. 370. Mineralogical Society, pp. 165–179, Apr. 1989. doi: 10.1180/minmag.1989.053.370.04.

α-Si_3N_4 (nano powder)

α-Si_3N_4 nano powder was purchased from Thermo Fischer Scientific. The purchased powder was dispersed on sticking carbon tape.

Bright field image

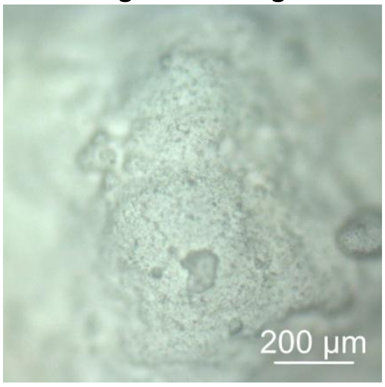

Dark field image

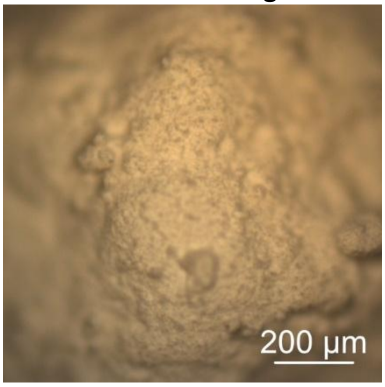

SE 10 keV

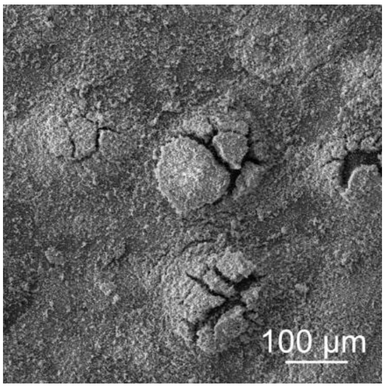

SE 1 keV

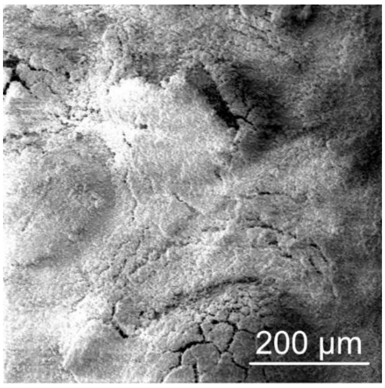

BSE 10 keV

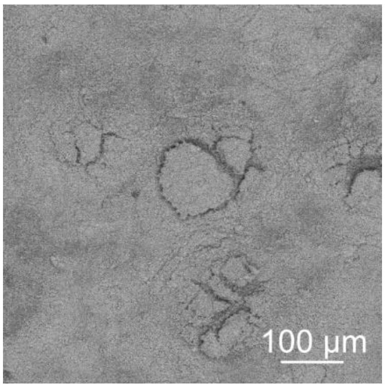

SE 10 keV (close up)

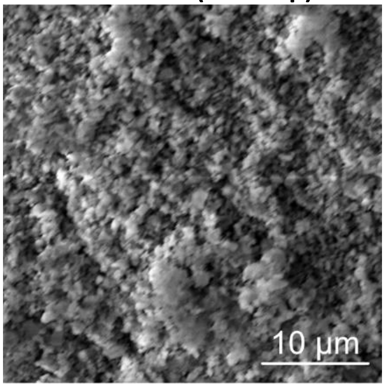

Monte Carlo simulations (CASINO)

d_e		$<d_{PE}>$ (d_{stdv})	
1 keV	30 keV	1 keV	30 keV
14.4 nm	347 nm	11.7 nm (4.0 nm)	3352 nm (1105 nm)
$<d_{BSE}>$ (d_{stdv})		β (BSE/SE)	
3.2 nm (2.1 nm)	1099 nm (565 nm)	0.147	0.104

Material parameters for simulations

ρ (g/cm^3)	Z	Z_{eff}
3.17	Si: 16 N: 7	10

Raman spectrum

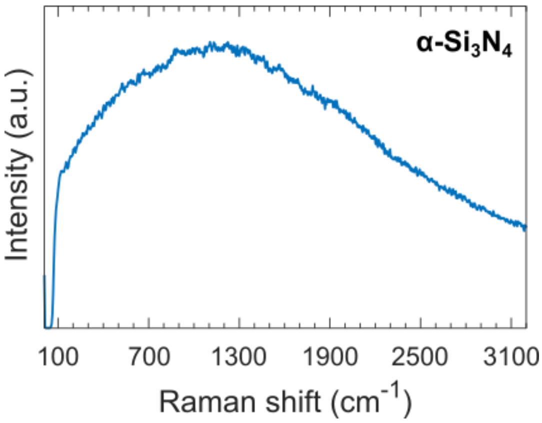

The Raman spectrum of α-Si_3N_4 nano powder was not measurable as the signal was covered by strong photoluminescence.

Si_3N_4 (20 nm membrane)

Si_3N_4 membranes (20 nm thickness) were purchased from SIMpore. The selected piece was fixed on a TEM specimen stub (Deltamicroscopies) to minimize background emission.

Bright field image

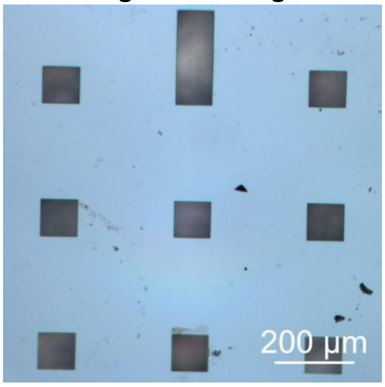

Dark field image

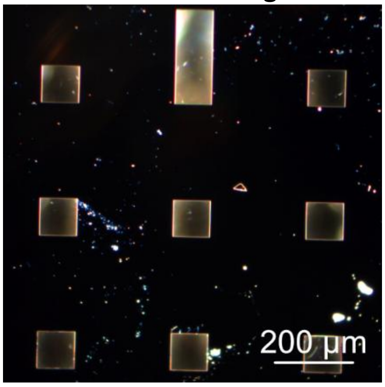

SE 30 keV

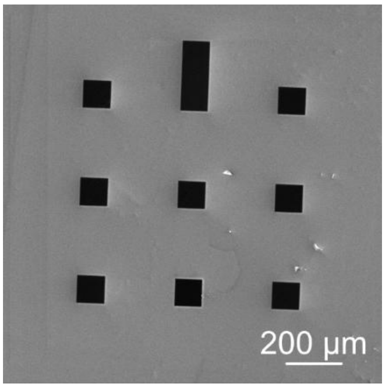

SE 1 keV

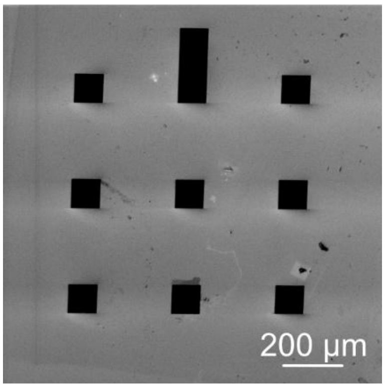

BSE 30 keV

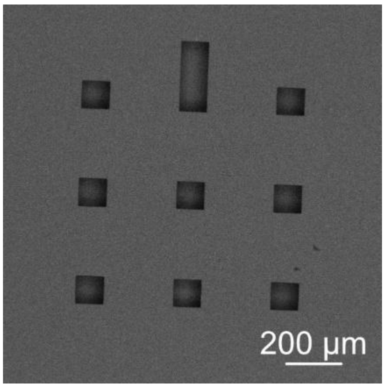

SE 1 keV (close up)

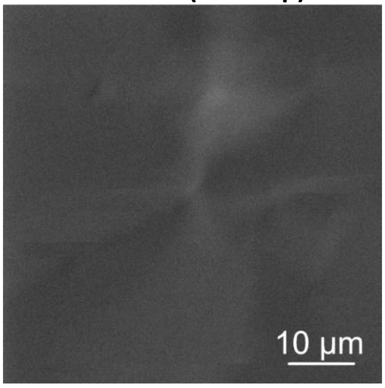

Raman spectrum

Due to the membrane's insufficient thickness, reliable Raman spectroscopy measurements could not be obtained.

β-Si_3N_4

β-Si_3N_4 bulk substrates were purchased from MSE-Supplies. The selected substrate was fixed on a SEM specimen stub with the help of sticking carbon tape.

Bright field image

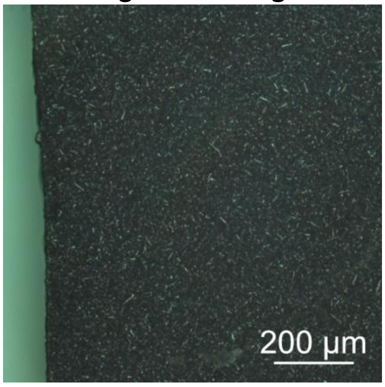

Dark field image

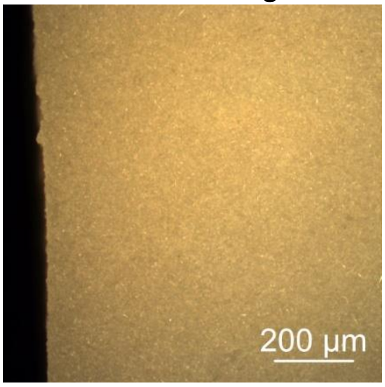

SE 10 keV

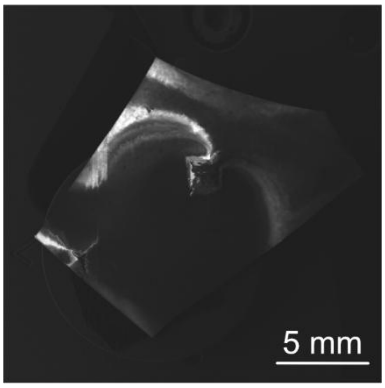

SE 1 keV

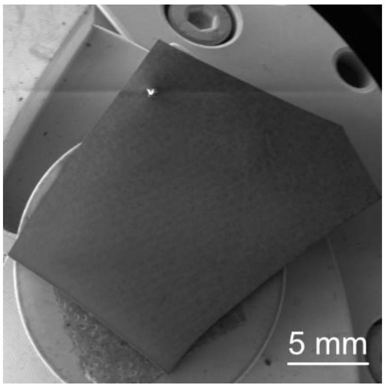

BSE 10 keV

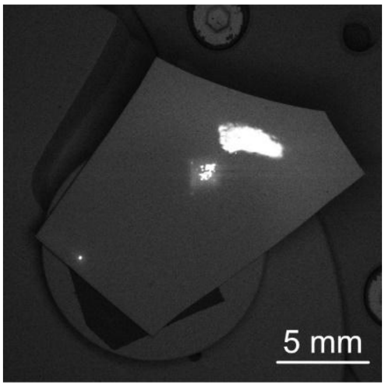

SE 10 keV (close up)

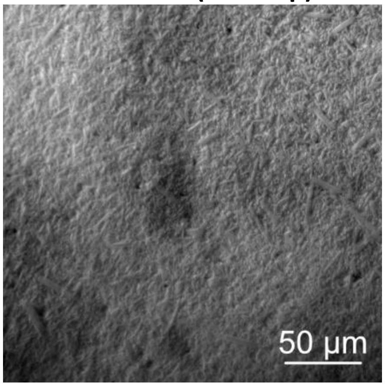

Monte Carlo simulations (CASINO)

d_e		$\langle d_{PE} \rangle$ (d_{stdv})	
1 keV	30 keV	1 keV	30 keV
14.4 nm	347 nm	11.7 nm (4.0 nm)	3352 nm (1105 nm)
$\langle d_{BSE} \rangle$ (d_{stdv})		β (BSE/SE)	
3.2 nm (2.1 nm)	1099 nm (565 nm)	0.147	0.104

Material parameters for simulations

ρ (g/cm^3)	Z	Z_{eff}
3.17	Si: 16 N: 7	10

Raman spectrum

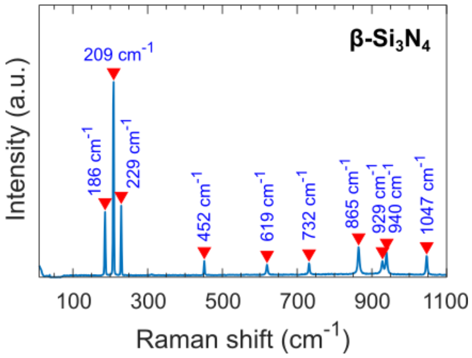

Bulk β-Si_3N_4 exhibits several vibrational modes in Raman spectrum, matching distinct phonon frequencies. The peak at 186 cm^{-1} (E_{2g}) corresponds to a lattice vibration involving bending of Si–N bonds, while 209 cm^{-1} (A_{1g}) and 220 cm^{-1} ($E_{1}g$) are assigned to low-frequency bending modes. The prominent peak at 452 cm^{-1} (E_{2g}) is associated with Si–N–Si bending vibrations, and 619 cm^{-1} (A_{1g}) corresponds to Si–N stretching. Higher-frequency peaks include 732 cm^{-1} (E_{1g}) and 865 cm^{-1} (E_{2g}), representing asymmetric Si–N stretching modes. The sharp peaks at 929 cm^{-1} (A_{1g}) and 940 cm^{-1} (E_{2g}) are attributed to symmetric stretching of the Si–N bonds within the tetrahedral network, while 1047 cm^{-1} (E_{1g}) represents high-energy asymmetric stretching modes [23].

[23] J. T. Kehren, M. Fischer, and O. Krause, "The Raman Spectra of α- and β-Si3N4 and Si2N2O Determined Experimentally and by Density Functional Theory," Journal of Raman Spectroscopy. Wiley, Nov. 14, 2024. doi: 10.1002/jrs.6749.

4H-SiC

4H-SiC substrates were purchased from MSE-Supplies. The selected substrate was fixed on a SEM specimen stub with the help of sticking carbon tape.

Bright field image

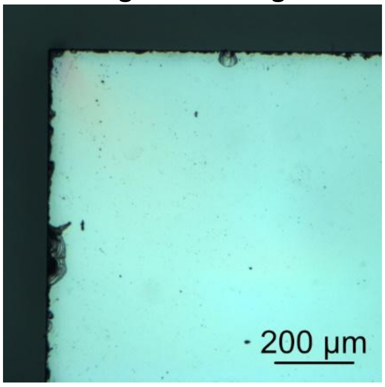

Dark field image

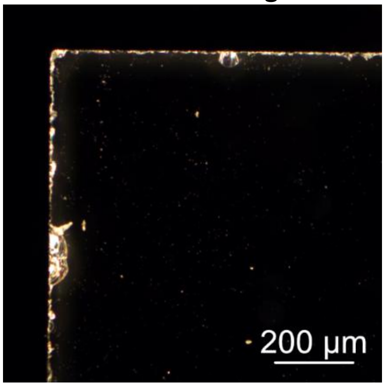

SE 30 keV

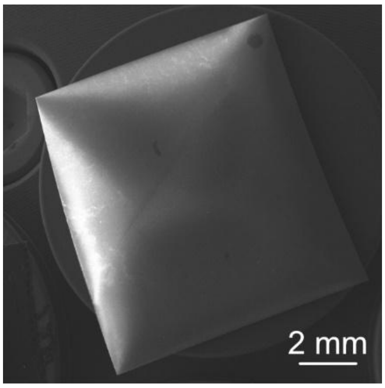

SE 1 keV

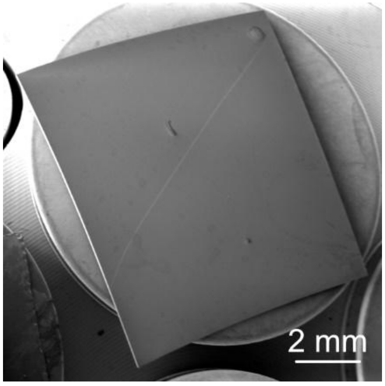

BSE 30 keV

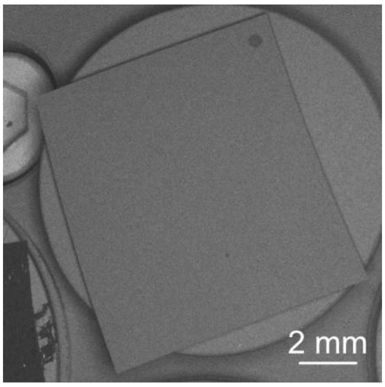

SE 1 keV (close up)

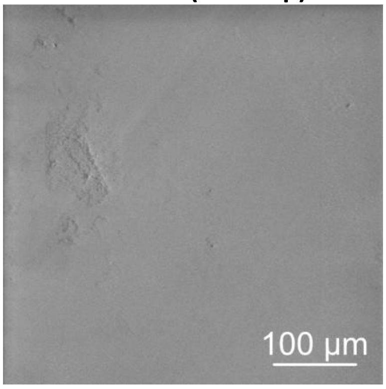

Monte Carlo simulations (CASINO)

d_e		<d_{PE}> (d_{stdv})	
1 keV	30 keV	1 keV	30 keV
13.6 nm	280.9 nm	11.8 nm (4.1 nm)	3339 nm (1107 nm)
<d_{BSE}> (d_{stdv})		β (BSE/SE)	
3.3 nm (2.1 nm)	1098 nm (565 nm)	0.152	0.108

Material parameters for simulations

ρ (g/cm^3)	Z	Z_{eff}
3.16	Si: 16 C: 6	10

Raman spectrum

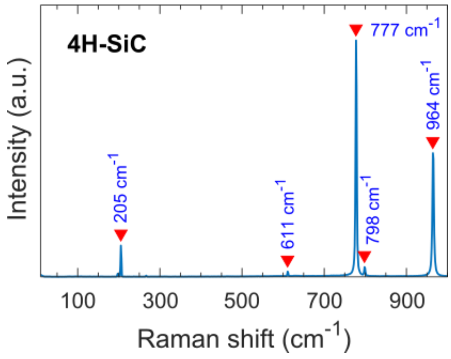

4H-SiC exhibits a distinctive Raman spectrum reflecting its hexagonal crystal structure with both acoustic and optical phonon peaks. The acoustic modes include the folded transverse acoustic mode at 205 cm^{-1} and the folded longitudinal acoustic mode at 611 cm^{-1}. Additionally, three prominent optical phonon peaks are observed: 777 cm^{-1} (folded transverse optical), 798 cm^{-1} (folded transverse optical), and 964 cm^{-1} (folded longitudinal optical) [24].

[24] M. Wei, Y. Li, S. Zhao, J. Jiao, G. Yan, and X. Liu, "Cristobalite formation on high-temperature oxidation of 4H-SiC surface based on silicon atom sublimation," Materials Today Communications, vol. 40. Elsevier BV, p. 110083, Aug. 2024. doi: 10.1016/j.mtcomm.2024.110083.

6H-SiC

6H-SiC substrates were purchased from MSE-Supplies. The selected substrate was fixed on a SEM specimen stub with the help of sticking carbon tape.

Bright field image

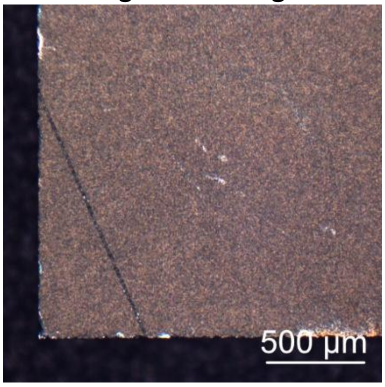

Dark field image

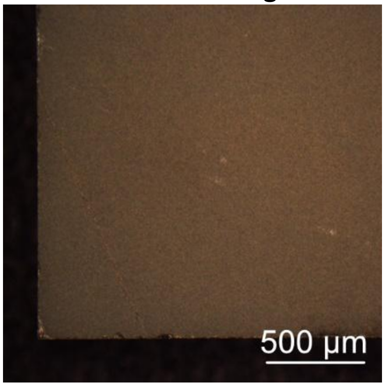

SE 30 keV

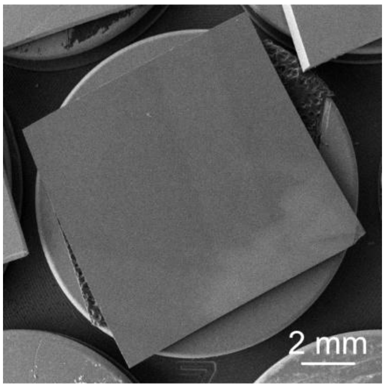

SE 1 keV

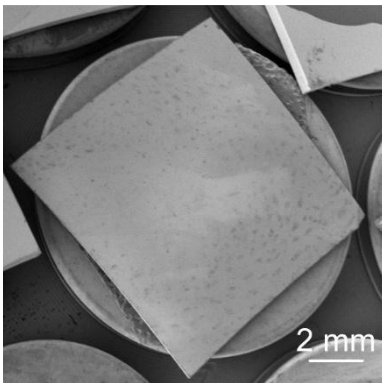

BSE 30 keV

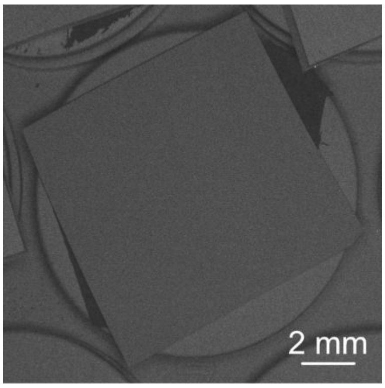

SE 1 keV (close up)

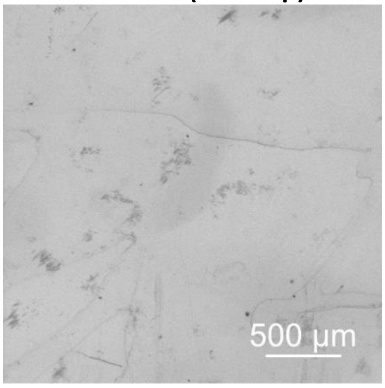

Raman spectrum

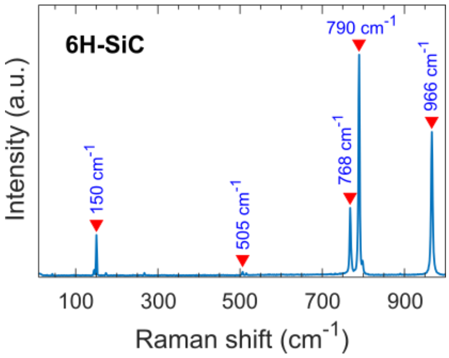

6H-SiC exhibits a distinctive Raman spectrum reflecting its hexagonal crystal structure with both acoustic and optical phonon peaks. The acoustic modes include the folded transverse acoustic mode at 150 cm^{-1} and the folded longitudinal acoustic mode at 505 cm^{-1}. Additionally, three prominent optical phonon peaks are observed: 768 cm^{-1} (folded transverse optical), 790 cm^{-1} (folded transverse optical), and 966 cm^{-1} (folded longitudinal optical) [25].

[25] D. W. Feldman, J. H. Parker, W. J. Choyke, and L. Patrick, “Raman Scattering in SiC,” Physical Review, vol. 170, no. 3. American Physical Society (APS), pp. 698–704, Jun. 15, 1968. doi: 10.1103/physrev.170.698

TiO_2

TiO_2 nano powder was purchased from Thermo Fischer Scientific. The purchased powder was dispersed on sticking carbon tape.

Bright field image

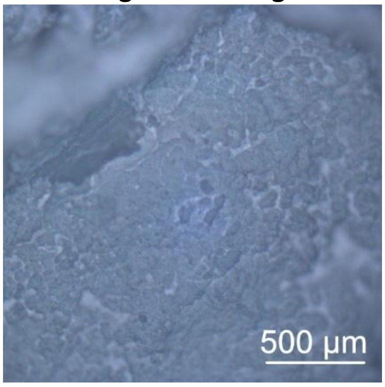

Dark field image

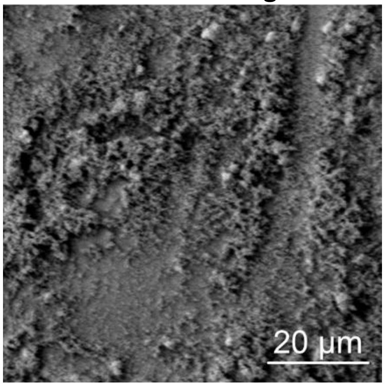

SE 10 keV

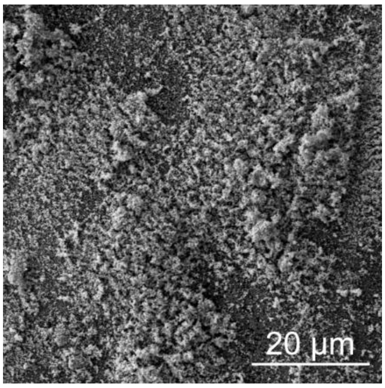

SE 1 keV

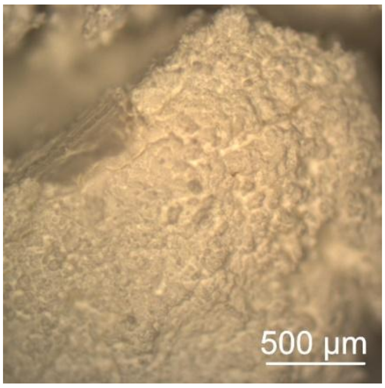

BSE 10 keV

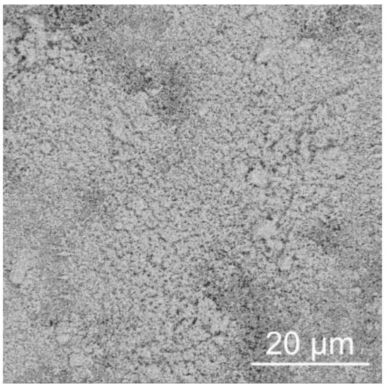

SE 1 keV (close up)

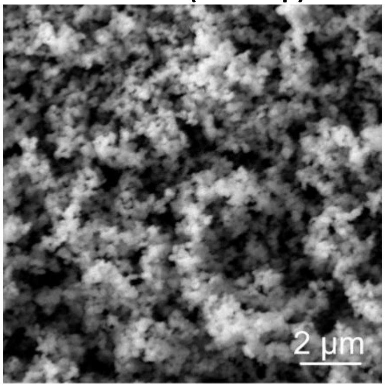

Monte Carlo simulations (CASINO)

d_e		$<d_{PE}>$ (d_{stdv})	
1 keV	30 keV	1 keV	30 keV
11.7 nm	270.6 nm	10.4 nm (3.7 nm)	2629 nm (919 nm)
$<d_{BSE}>$ (d_{stdv})		β (BSE/SE)	
2.9 nm (1.9 nm)	896 nm (462 nm)	0.190	0.159

Material parameters for simulations

ρ (g/cm^3)	Z	Z_{eff}
3.90	Ti: 22 O: 8	12.41

Raman spectrum

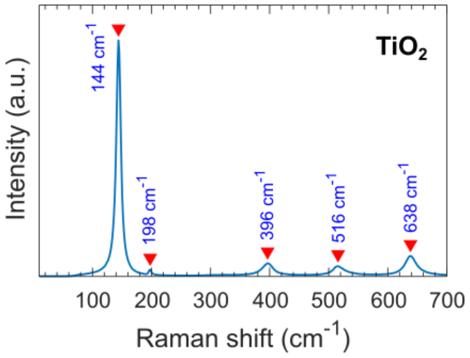

We observe Raman modes in TiO2 at 144 cm^{-1} (Eg), 198 cm^{-1} (Eg), 396 cm^{-1} (B1g), 516 cm^{-1} (A1g), and 638 cm^{-1} (Eg), which are characteristic of the anatase phase of TiO_2. These modes correspond to the symmetric stretching, bending, and lattice vibrations typical of the anatase crystal structure, distinguishing it from other polymorphs such as rutile or brookite [26].

[26] Y.-H. Zhang, C. K. Chan, J. F. Porter, and W. Guo, “Micro-Raman Spectroscopic Characterization of Nanosized TiO2 Powders Prepared by Vapor Hydrolysis,” Journal of Materials Research, vol. 13, no. 9. Springer Science and Business Media LLC, pp. 2602–2609, Sep. 1998. doi: 10.1557/jmr.1998.0363.

ITO

ITO (In_2O_3/SnO_2 (90/10)) was purchased in the form of ITO evaporation pieces from Kurt J. Lesker. The ITO has a purity of 99.99%. The selected ITO piece was fixed on a SEM specimen stub with the help of sticking carbon tape. Because ITO has an inhomogeneous composition, we do not provide Monte Carlo simulations for this material.

Bright field image

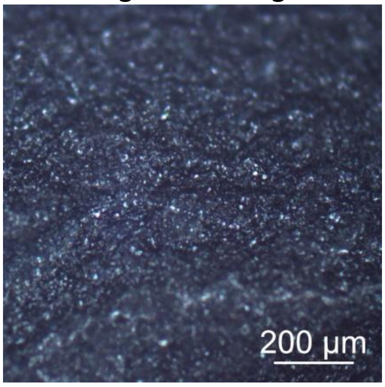

Dark field image

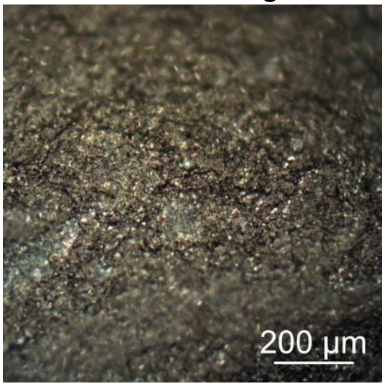

SE 30 keV

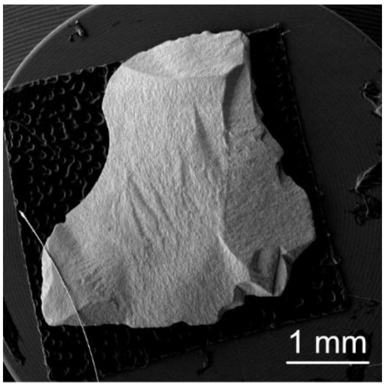

SE 1 keV

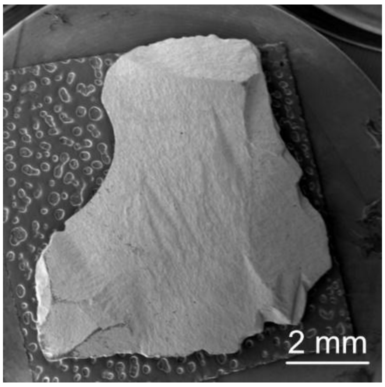

BSE 30 keV

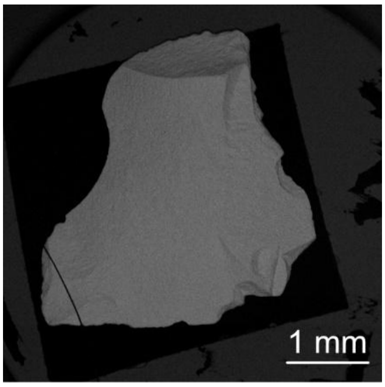

SE 30 keV (close up)

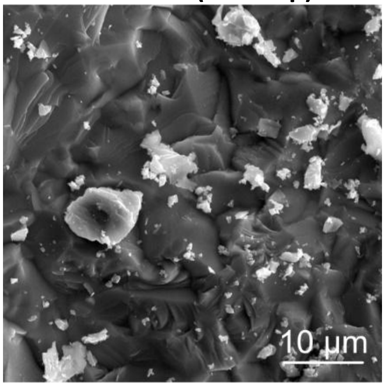

Raman spectrum

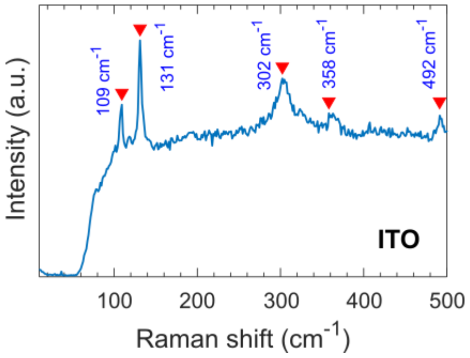

ITO exhibits characteristic Raman-active vibrational modes primarily associated with its indium oxide (In_2O_3) component. The approximate positions of these features are highlighted in the accompanying figure [27].

[27] H. Ullah, Z. H. Yamani, A. Qurashi, J. Iqbal, and K. Safeen, “Study of the optical and gas sensing properties of In2O3 nanoparticles synthesized by rapid sonochemical method,” Journal of Materials Science: Materials in Electronics, vol. 31, no. 20. Springer Science and Business Media LLC, pp. 17474–17481, Aug. 31, 2020. doi: 10.1007/s10854-020-04303-9.

ZrO_2

ZrO_2 was purchased in the form of ZrO_2 evaporation pieces from Kurt J. Lesker. The ZrO_2 has a purity of 99.9%. The selected ZrO_2 piece was fixed on a SEM specimen stub with the help of sticking carbon tape.

Bright field image

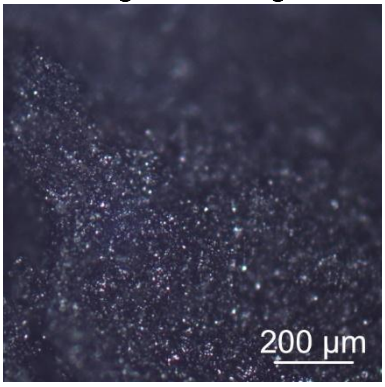

Dark field image

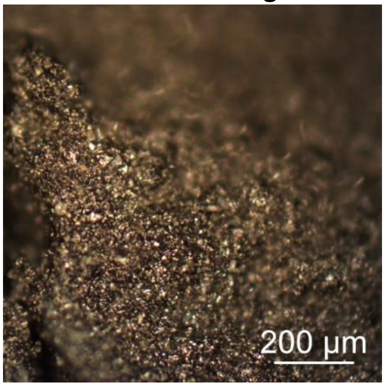

SE 30 keV

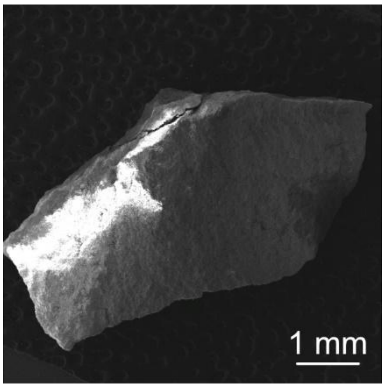

SE 1 keV

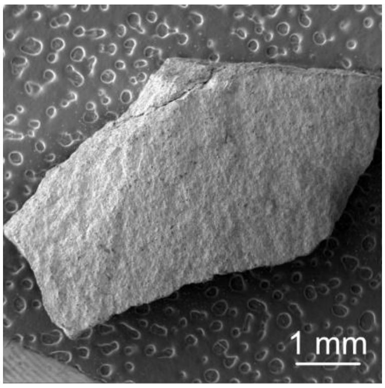

BSE 30 keV

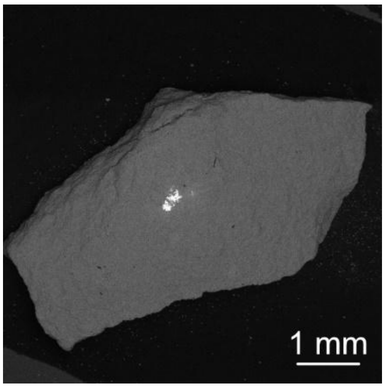

SE 1 keV (close up)

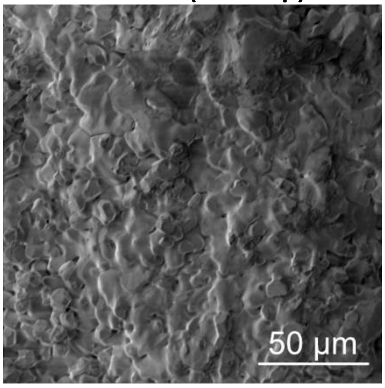

Monte Carlo simulations (CASINO)

d_e		$\langle d_{PE} \rangle$ (d_{stdv})	
1 keV	30 keV	1 keV	30 keV
9.8 nm	149 nm	9.1 nm (3.4 nm)	1691 nm (621 nm)
$\langle d_{BSE} \rangle$ (d_{stdv})		β (BSE/SE)	
2.6 nm (1.7 nm)	571 nm (318 nm)	0.259	0.281

Material parameters for simulations

ρ (g/cm^3)	Z	Z_{eff}
5.68	Zr: 40 O: 8	17.76

Raman spectrum

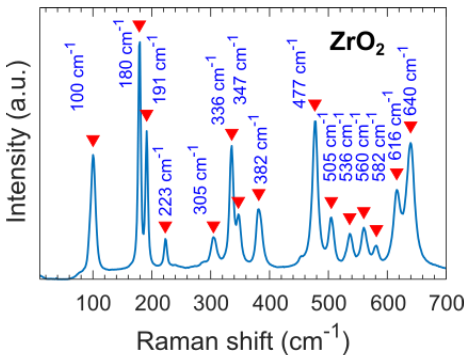

Modes up to 700 cm^{-1} originate from fundamental lattice vibrations, primarily involving Zr–O stretching and bending motions. The approximate positions of these features are highlighted in the accompanying figure [28].

[28] G. G. Siu, M. J. Stokes, and Y. Liu, "Variation of fundamental and higher-order Raman spectra of ZrO2 nanograins with annealing temperature," Physical Review B, vol. 59, no. 4. American Physical Society (APS), pp. 3173–3179, Jan. 15, 1999. doi: 10.1103/physrevb.59.3173.

Ta_2O_5

Ta_2O_5 micro powder was purchased from Thermo Fischer Scientific. The purchased powder was dispersed on sticking carbon tape.

Bright field image

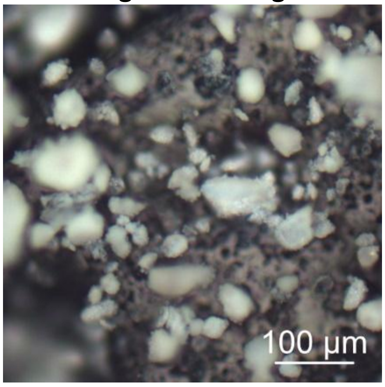

Dark field image

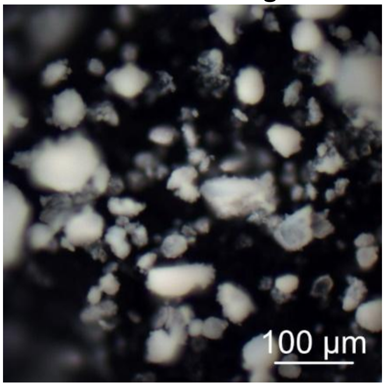

SE 10 keV

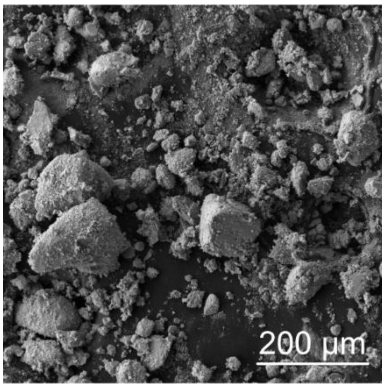

SE 1 keV

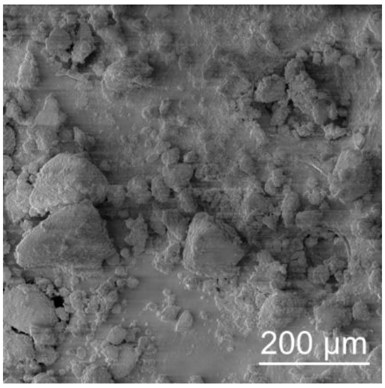

BSE 10 keV

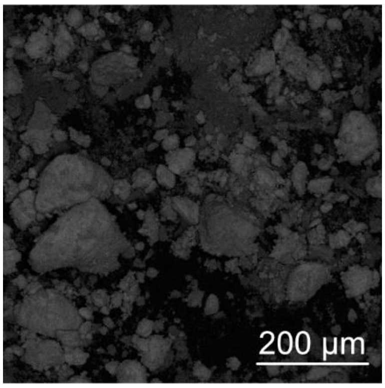

SE 10 keV (close up)

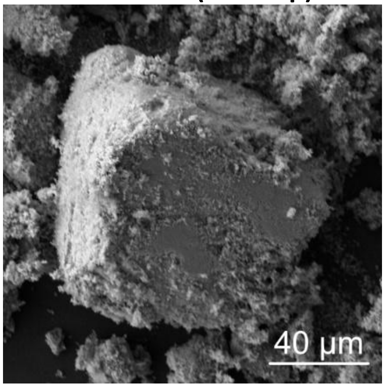

Monte Carlo simulations (CASINO)

d_e		$<d_{PE}>$ (d_{stdv})	
1 keV	30 keV	1 keV	30 keV
9.4 nm	104 nm	8.8 nm (3.1 nm)	1182 nm (432 nm)
$<d_{BSE}>$ (d_{stdv})		β (BSE/SE)	
2.4 nm (1.7 nm)	373 nm (234 nm)	0.296	0.404

Material parameters for simulations

ρ (g/cm^3)	Z	Z_{eff}
8.24	Ta: 73 O: 8	23.86

Raman spectrum

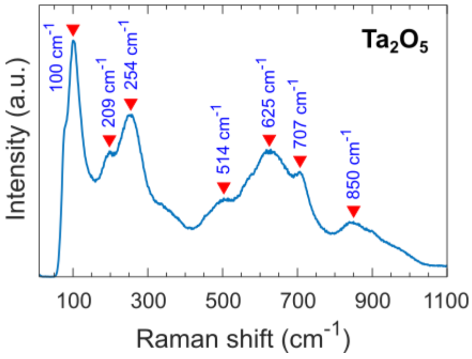

In the low-frequency range (~100–250 cm^{-1}), modes correspond to lattice vibrations and bending of Ta–O–Ta linkages. The mid-frequency region (~250–550 cm^{-1}) is dominated by bending and symmetric stretching vibrations of Ta–O bonds. In the high-frequency range (~600–900 cm^{-1}), the Raman modes are primarily attributed to asymmetric stretching of Ta–O bonds and stretching involving terminal oxygen atoms. The approximate positions of these features are highlighted in the accompanying figure [29].

[29] C. Joseph, P. Bourson, and M. D. Fontana, “Amorphous to crystalline transformation in Ta2O5 studied by Raman spectroscopy,” Journal of Raman Spectroscopy, vol. 43, no. 8. Wiley, pp. 1146–1150, Jun. 07, 2012. doi: 10.1002/jrs.3142.

MoO_3

MoO_3 was purchased in the form of MoO_3 evaporation pieces from Kurt J. Lesker. The MoO_3 has a purity of 99.95%. The selected MoO_3 piece was fixed on a SEM specimen stub with the help of sticking carbon tape.

Bright field image

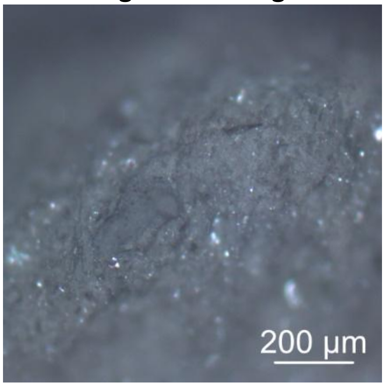

Dark field image

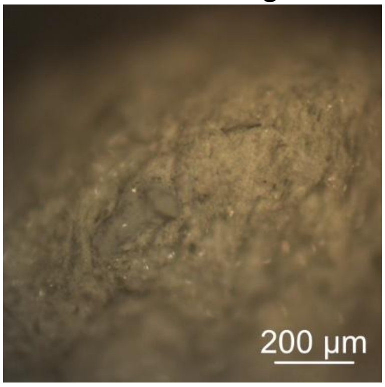

SE 30 keV

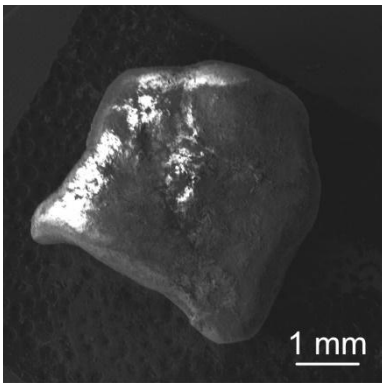

SE 1 keV

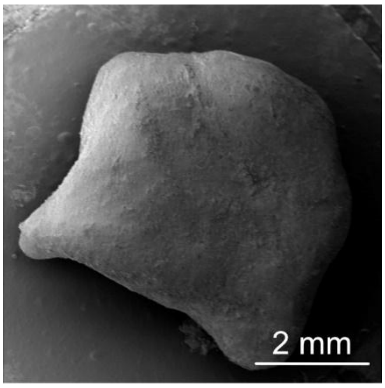

BSE 30 keV

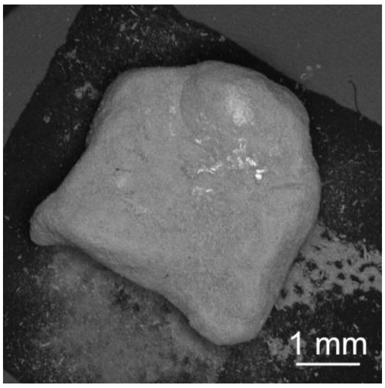

SE 30 keV (close up)

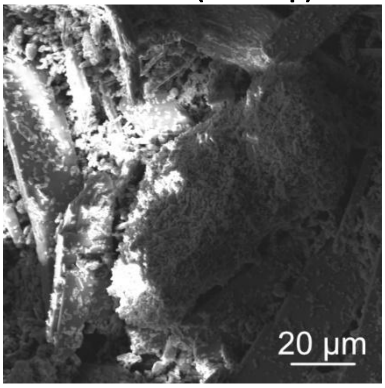

Monte Carlo simulations (CASINO)

d_e		$\langle d_{PE} \rangle$ (d_{stdv})	
1 keV	30 keV	1 keV	30 keV
11.6 nm	209 nm	10.7 nm (3.9 nm)	2049 nm (751 nm)
$\langle d_{BSE} \rangle$ (d_{stdv})		β (BSE/SE)	
3.0 nm (2.0 nm)	692 nm (384 nm)	0.241	0.267

Material parameters for simulations

ρ (g/cm^3)	Z	Z_{eff}
4.70	Mo: 42 O: 8	15.74

Raman spectrum

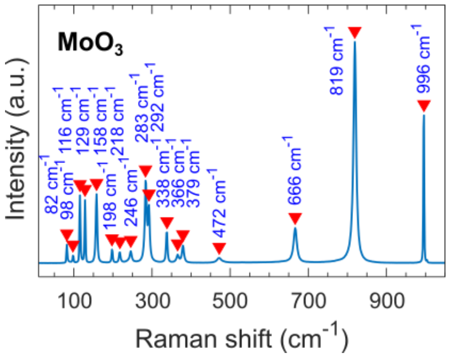

MoO3 exhibits multiple peaks associated with bending, stretching, and twisting of Mo–O bonds. We observe Raman peaks at 996 cm^{-1}, 819 cm^{-1}, and 666 cm^{-1}, which correspond to the orthorhombic (o-MoO_3) phase of molybdenum trioxide. The 996 cm^{-1} peak is attributed to the Mo=O terminal stretching mode, the 819 cm^{-1} peak corresponds to stretching vibrations of Mo-O-Mo units, and the 666 cm^{-1} peak is associated with bridging oxygen vibrations in the MoO_3 lattice. The Raman modes of MoO_3 up to 400 cm^{-1} correspond to lattice vibrations, Mo–O bending modes, and low-energy stretching interactions. The approximate positions of these Raman lines are indicated in the accompanying figure [30].

[30] L. Seguin, M. Figlarz, R. Cavagnat, and J.-C. Lassègues, “Infrared and Raman spectra of MoO3 molybdenum trioxides and MoO3 · xH2O molybdenum trioxide hydrates,” Spectrochimica Acta Part A: Molecular and Biomolecular Spectroscopy, vol. 51, no. 8. Elsevier BV, pp. 1323–1344, Jul. 1995. doi: 10.1016/0584-8539(94)00247-9.

$LiNbO_3$

$LiNbO_3$ (y-cut) was purchased in the form of crystalline $LiNbO_3$ wafers. The $LiNbO_3$ wafer was cleaved into sized pieces. The selected germanium piece was fixed on a SEM specimen stub with the help of sticking carbon tape. The displayed optical images show the damage generated during the CL measurement procedure.

Bright field image

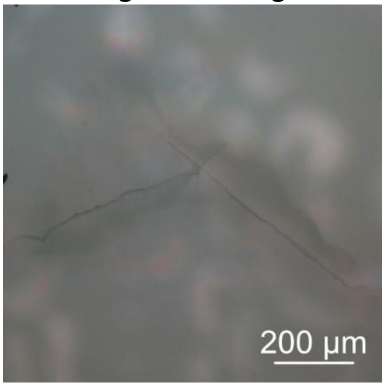

Dark field image

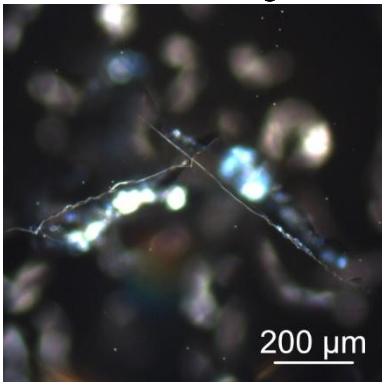

SE 10 keV

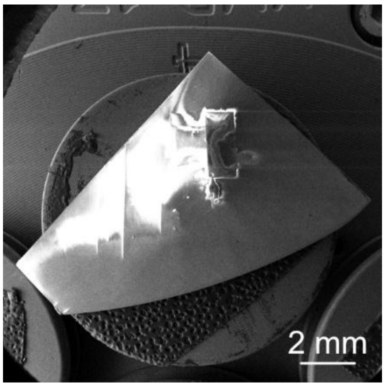

SE 1 keV

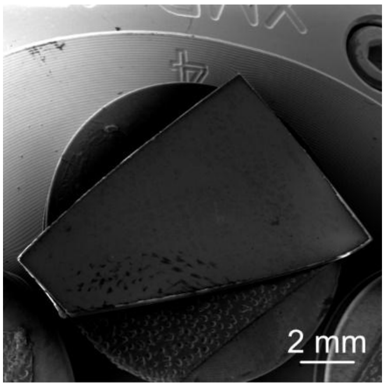

BSE 10 keV

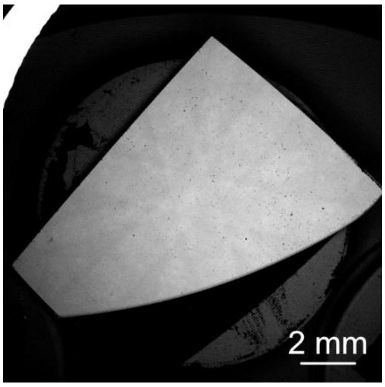

SE 1 keV (close up)

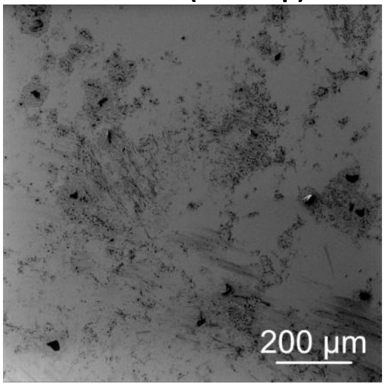

Monte Carlo simulations (CASINO)

d_e		<d_{PE}> (d_{stdv})	
1 keV	30 keV	1 keV	30 keV
12.5 nm	232 nm	11.3 nm (4.1 nm)	2245 nm (820 nm)
<d_{BSE}> (d_{stdv})		β (BSE/SE)	
3.1 nm (2.1 nm)	756 nm (418 nm)	0.225	0.250

Material parameters for simulations

ρ (g/cm^3)	Z	Z_{eff}
4.30	Li: 3 Nb: 41 O: 8	13.24

Raman spectrum

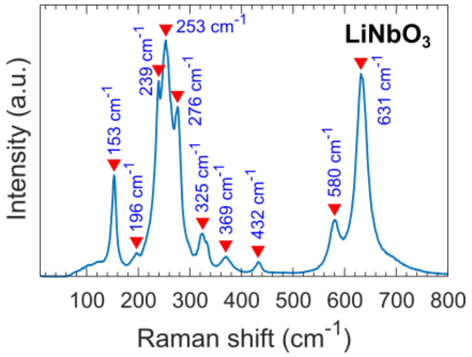

$LiNbO_3$ in the Y-cut orientation exhibits Raman peaks associated with stretching and bending of Nb–O bonds and lattice vibrations of the $LiNbO_3$ structure. Prominent peaks typically appear in the low-frequency region (~150–300 cm^{-1}) for lattice vibrations and in the high-frequency region (~600–700 cm^{-1}) for Nb–O stretching modes. The approximate positions of observed vibrational modes are indicated in the accompanying figure [31].

[31] Y. Zhang, L. Guilbert, and P. Bourson, "Characterization of Ti : LiNbO3 waveguides by micro-raman and luminescence spectroscopy," Applied Physics B, vol. 78, no. 3–4. Springer Science and Business Media LLC, pp. 355–361, Feb. 2004. doi: 10.1007/s00340-004-1402-0.

Al_2O_3

Sapphire (Al_2O_3) crystals were purchased from Kurt J. Lesker. The sapphire has a purity of 99.99%. The selected sapphire crystal was fixed on a SEM specimen stub with the help of sticking carbon tape.

Bright field image

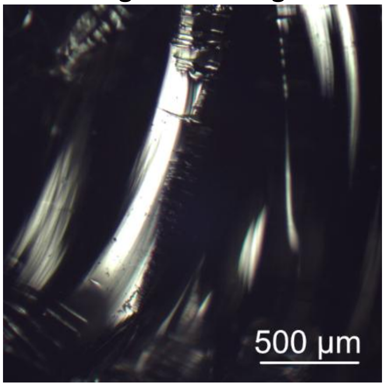

Dark field image

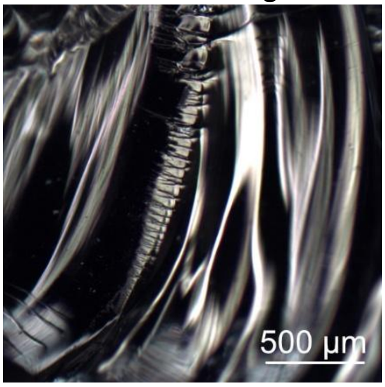

SE 10 keV

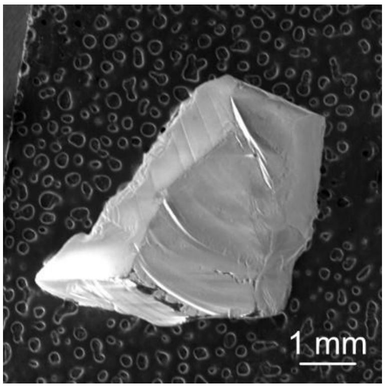

SE 1 keV

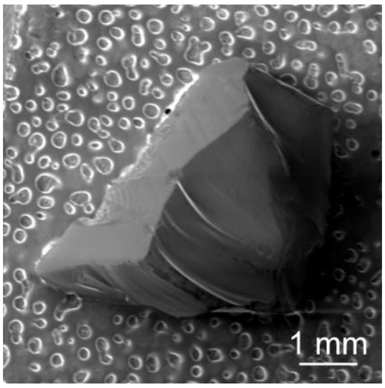

BSE 10 keV

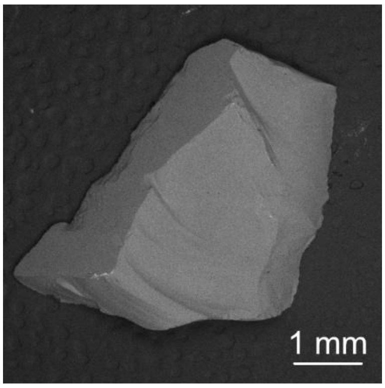

SE 10 keV (close up)

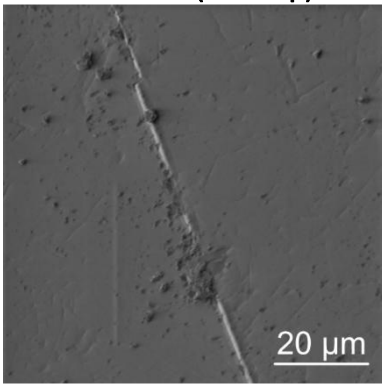

Monte Carlo simulations (CASINO)

d_e		$\langle d_{PE} \rangle$ (d_{stdv})	
1 keV	30 keV	1 keV	30 keV
10.8 nm	263 nm	9.4 nm (3.2 nm)	2753 nm (895 nm)
$\langle d_{BSE} \rangle$ (d_{stdv})		β (BSE/SE)	
2.6 nm (1.7 nm)	891 nm (460 nm)	0.144	0.096

Material parameters for simulations

ρ (g/cm^3)	Z	Z_{eff}
3.90	Al: 13 O: 8	13.24

Raman spectrum

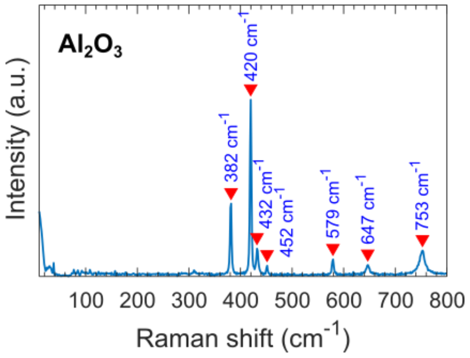

Aluminum oxide in its saphire (α-Al_2O_3) form exhibits a distinct Raman spectrum characterized by vibrational modes associated with the Al–O bonds and lattice dynamics. Observed modes correspond to internal vibrations of the AlO_6 octahedral units in the corundum structure, where the Eg modes represent doubly degenerate in-plane vibrations, while the A_1g modes correspond to symmetric stretching vibrations along the c-axis. These modes are visually highlighted in the accompanying figure [32].

[32] S. P. S. Porto and R. S. Krishnan, "Raman Effect of Corundum," The Journal of Chemical Physics, vol. 47, no. 3. AIP Publishing, pp. 1009–1012, Aug. 01, 1967. doi: 10.1063/1.1711980.

Yb_2O_3

Yb_2O_3 pieces were purchased from Kurt J. Lesker. The Yb_2O_3 has a purity of 99.9%. The selected Yb_2O_3 piece was fixed on a SEM specimen stub with the help of sticking carbon tape.

Bright field image

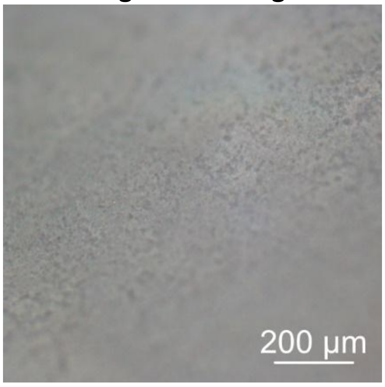

Dark field image

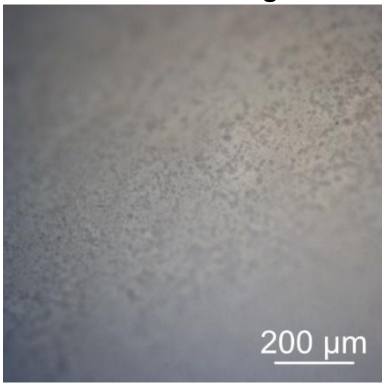

SE 10 keV

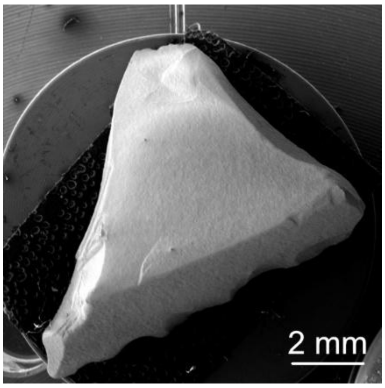

SE 1 keV

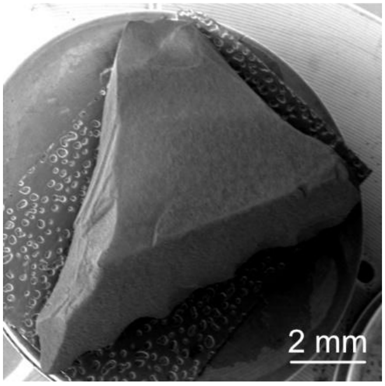

BSE 10 keV

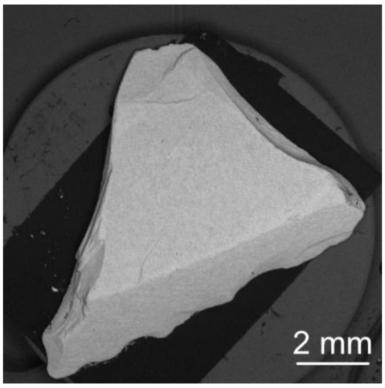

SE 10 keV (close up)

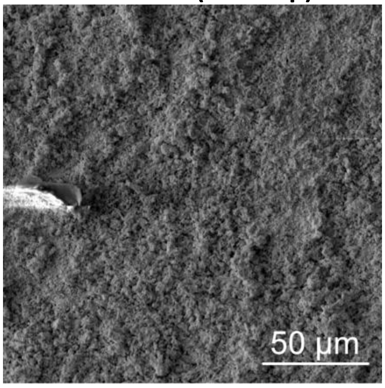

Monte Carlo simulations (CASINO)

d_e		<d_{PE}> (d_{stdv})	
1 keV	30 keV	1 keV	30 keV
8.7 nm	112 nm	8.8 nm (3.3 nm)	1066 nm (394nm)
<d_{BSE}> (d_{stdv})		β (BSE/SE)	
2.3 nm (1.7 nm)	334 nm (210 nm)	0.327	0.416

Material parameters for simulations

ρ (g/cm^3)	Z	Z_{eff}
9.2	Yb: 70 O: 8	29.72

Raman spectrum

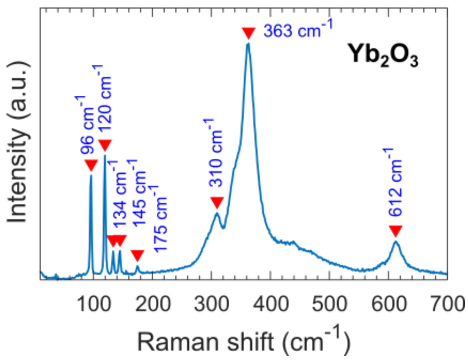

Yb_2O_3 exhibits Raman peaks that are associated with various phonon modes within the crystal lattice, including bending and stretching vibrations of Yb–O bonds. The approximate peak positions are visually highlighted in the accompanying figure [33].

[33] S. D. Pandey, K. Samanta, J. Singh, N. D. Sharma, and A. K. Bandyopadhyay, "Anharmonic behavior and structural phase transition in Yb2O3," AIP Advances, vol. 3, no. 12. AIP Publishing, Dec. 01, 2013. doi: 10.1063/1.4858421.

GaN

GaN nano powder was purchased from Thermo Fischer Scientific. The purchased powder was dispersed on sticking carbon tape.

Bright field image

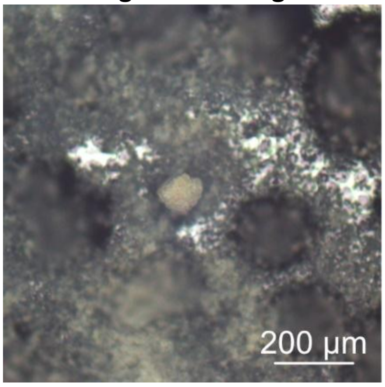

Dark field image

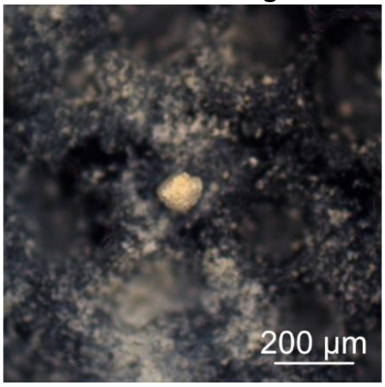

SE 10 keV

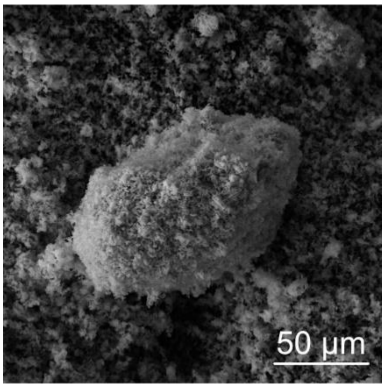

SE 1 keV

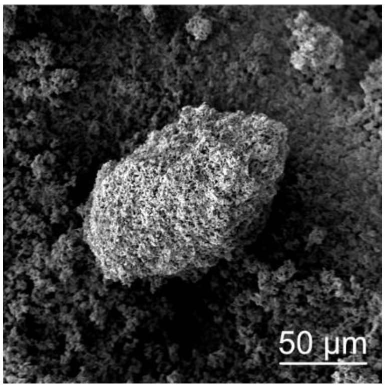

BSE 10 keV

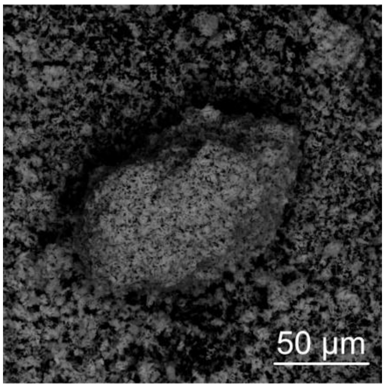

SE 10 keV (close up)

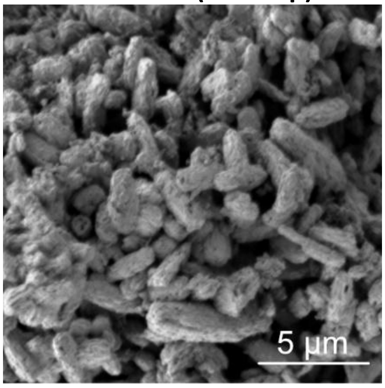

Monte Carlo simulations (CASINO)

d_e		$<d_{PE}>$ (d_{stdv})	
1 keV	30 keV	1 keV	30 keV
9.0 nm	159 nm	8.5 nm (3.2 nm)	1625 nm (590 nm)
$<d_{BSE}>$ (d_{stdv})		β (BSE/SE)	
2.3 nm (1.6 nm)	553 nm (300 nm)	0.265	0.255

Material parameters for simulations

ρ (g/cm^3)	Z	Z_{eff}
6.1	Ga: 31 N: 7	18.27

Raman spectrum

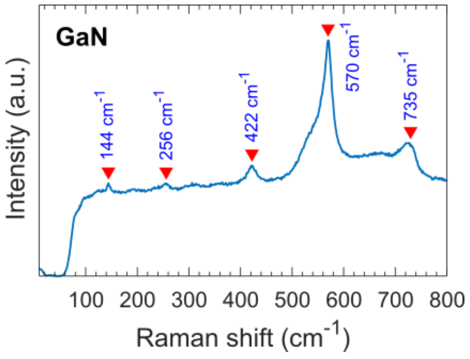

Raman spectrum of GaN includes the E_2 mode at approximately 144 cm^{-1} and 570 cm^{-1}, associated with non-polar optical phonon vibrations, and the A_1 (LO) mode near 735 cm^{-1}, linked to longitudinal optical phonon vibrations. The accompanying figure indicates the approximate positions of measured Raman lines [34].

[34] T. Azuhata, T. Sota, K. Suzuki, and S. Nakamura, “Polarized Raman spectra in GaN,” Journal of Physics: Condensed Matter, vol. 7, no. 10. IOP Publishing, pp. L129–L133, Mar. 06, 1995. doi: 10.1088/0953-8984/7/10/002.

GaP

GaP crystals were purchased from Thermo Fischer Scientific. The selected GaP crystal was fixed on a SEM specimen stub with the help of sticking carbon tape.

Bright field image

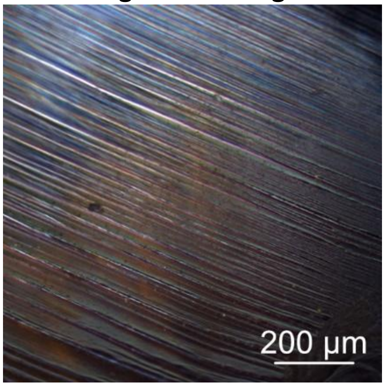

Dark field image

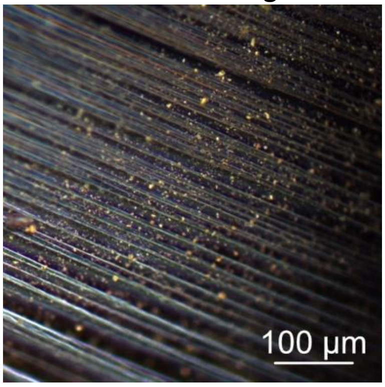

SE 30 keV

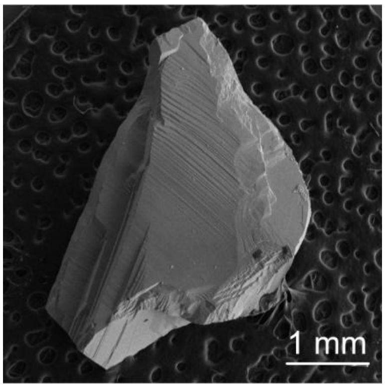

SE 1 keV

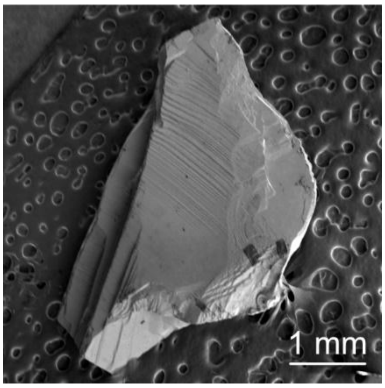

BSE 30 keV

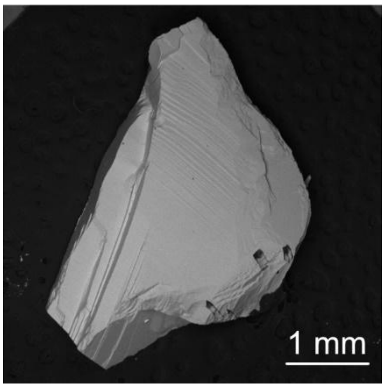

SE 1 keV (close up)

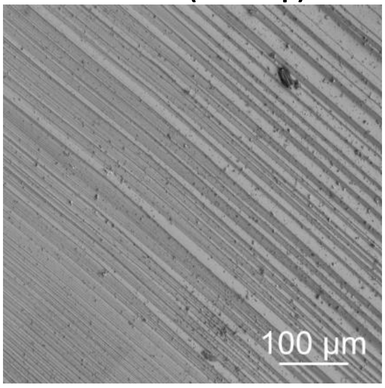

Monte Carlo simulations (CASINO)

d_e		$\langle d_{PE} \rangle$ (d_{stdv})	
1 keV	30 keV	1 keV	30 keV
13.0 nm	231 nm	12.4 nm (4.7 nm)	2398 nm (876 nm)
$\langle d_{BSE} \rangle$ (d_{stdv})		β (BSE/SE)	
3.5 nm (2.4 nm)	822 nm (443 nm)	0.274	0.253

Material parameters for simulations

ρ (g/cm^3)	Z	Z_{eff}
4.14	Ga: 31 P: 15	22.93

Raman spectrum

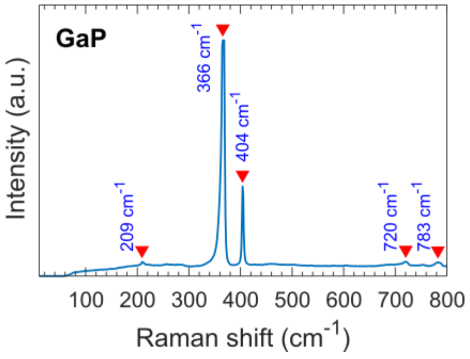

We observe in Raman spectrum of GaP the longitudinal optical (LO) phonon at approximately 404 cm^{-1}, which is associated with the vibrations of gallium and phosphorus atoms within the lattice. Additionally, the transverse optical (TO) phonon mode appears around 366 cm^{-1} [35].

[35] S. Hayashi and H. Kanamori, "Raman scattering from the surface phonon mode in GaP microcrystals," Physical Review B, vol. 26, no. 12. American Physical Society (APS), pp. 7079–7082, Dec. 15, 1982. doi: 10.1103/physrevb.26.7079.

MgF_2

We purchased crystalline MgF_2 substrates from Crystran, selected one for analysis, and mounted it on an SEM specimen stub using carbon tape.

Bright field image

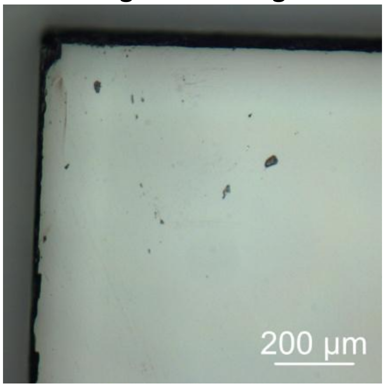

Dark field image

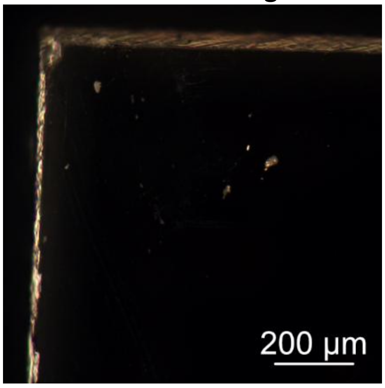

SE 10 keV

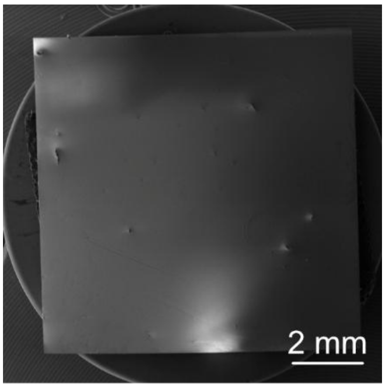

SE 1 keV (close up)

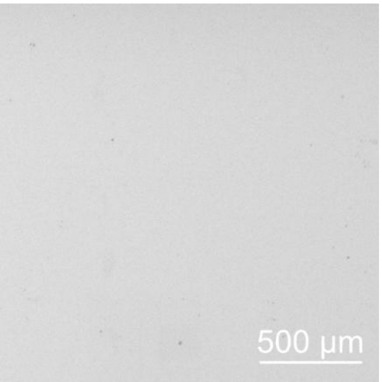

BSE 10 keV

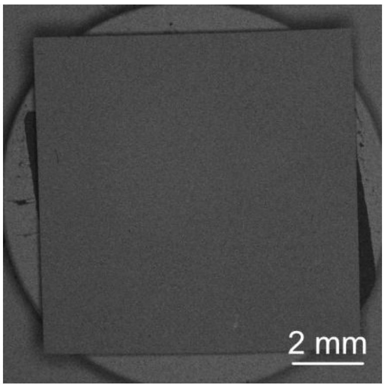

Monte Carlo simulations (CASINO)

d_e		$<d_{PE}>$ (d_{stdv})	
1 keV	30 keV	1 keV	30 keV
13.9 nm	285 nm	12.1 nm (4.2 nm)	3574 nm (1152 nm)
$<d_{BSE}>$ (d_{stdv})		β (BSE/SE)	
3.3 nm (2.1 nm)	1152 nm (594 nm)	0.140	0.089

Material parameters for simulations

ρ (g/cm^3)	Z	Z_{eff}
3.15	Mg: 12 F: 9	10.03

Raman spectrum

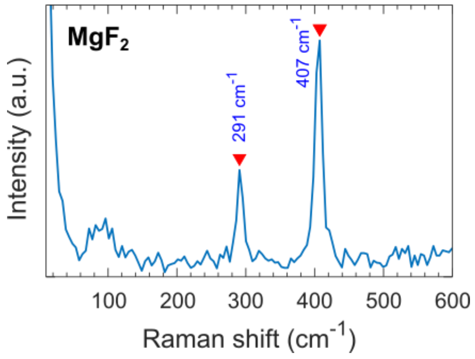

We observe Raman peaks at 291 cm^{-1} and 407 cm^{-1}, which correspond to the Eg and A_1g vibrational modes, respectively. These modes are characteristic of the rutile-type structure of MgF_2, where the A_1g mode involves symmetric stretching of the Mg–F bonds, and the Eg mode corresponds in-plane shearing vibrations of fluorine atoms within the lattice [36].

[36] X. Zhang et al., "High Pressure–Temperature Study of MgF2, CaF2, and BaF2 by Raman Spectroscopy: Phase Transitions and Vibrational Properties of AF2 Difluorides," ACS Omega, vol. 9, no. 22. American Chemical Society (ACS), pp. 23675–23687, May 22, 2024. doi: 10.1021/acsomega.4c01347

CaF_2

CaF_2 micro powder was purchased from Thermo Fischer Scientific. The purchased powder was dispersed on sticking carbon tape.

Bright field image

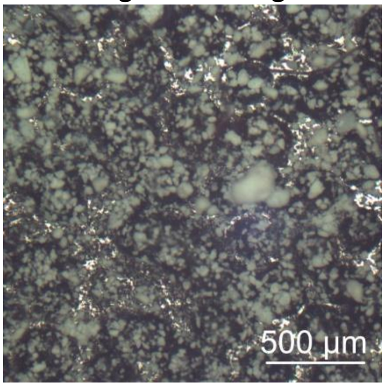

Dark field image

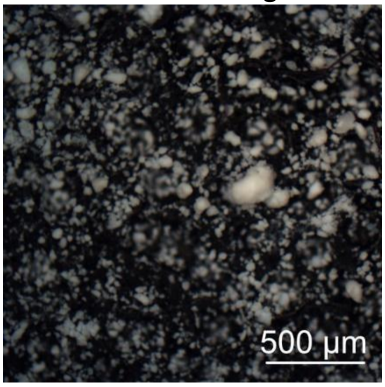

SE 10 keV

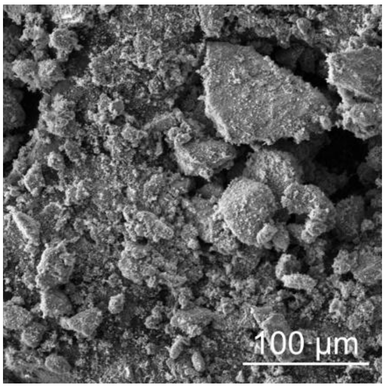

SE 1 keV

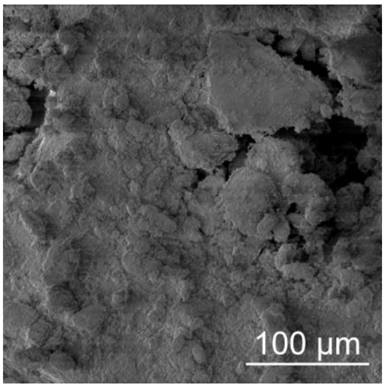

BSE 10 keV

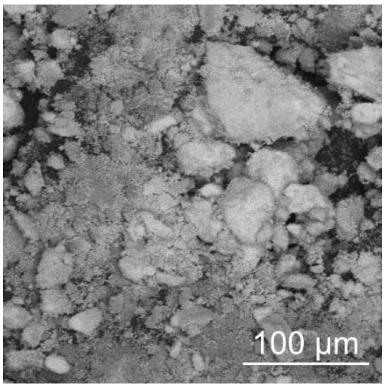

SE 10 keV (close up)

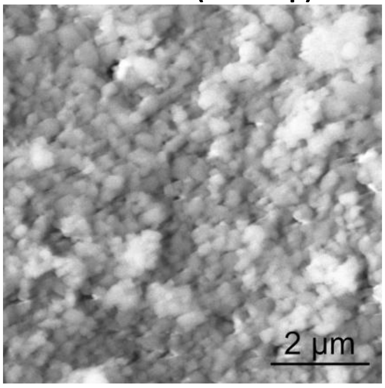

Monte Carlo simulations (CASINO)

d_e		$\langle d_{PE} \rangle$ (d_{stdv})	
1 keV	30 keV	1 keV	30 keV
13.9 nm	330 nm	3.5 nm (2.3 nm)	1087 nm (561 nm)
$\langle d_{BSE} \rangle$ (d_{stdv})		β (BSE/SE)	
2.3 nm (1.6 nm)	553 nm (300 nm)	0.183	0.146

Material parameters for simulations

ρ (g/cm³)	Z	Z_{eff}
3.18	Ca: 20 F: 9	12.8

Raman spectrum

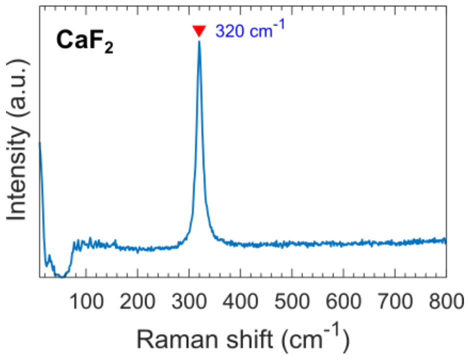

Calcium fluoride (CaF_2) exhibits a characteristic Raman spectrum with a prominent peak at approximately 320 cm^{-1}, corresponding to the T_{2g} symmetric stretching mode of the fluorite crystal structure [36].

BaF_2

We acquired crystalline BaF_2 substrates from Crystran, chose one for examination, and attached it to an SEM specimen stub using carbon tape.

Bright field image

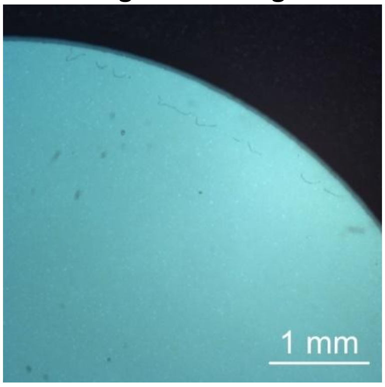

Dark field image

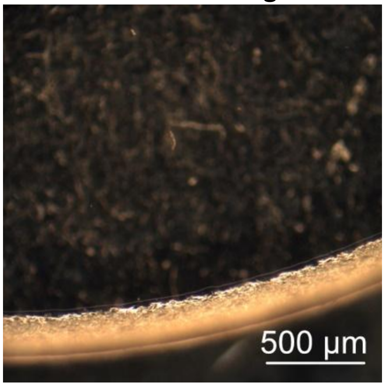

SE 10 keV

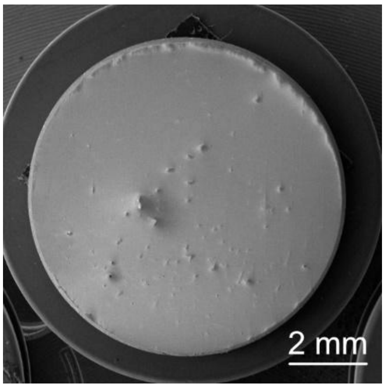

SE 1 keV

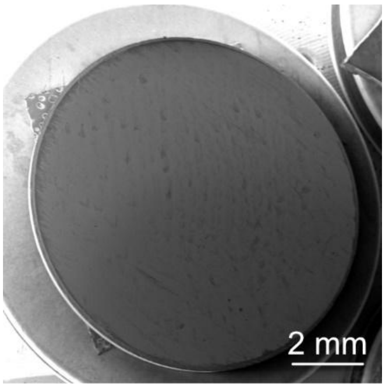

BSE 10 keV

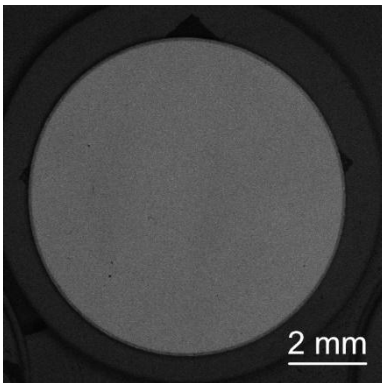

SE 1 keV (close up)

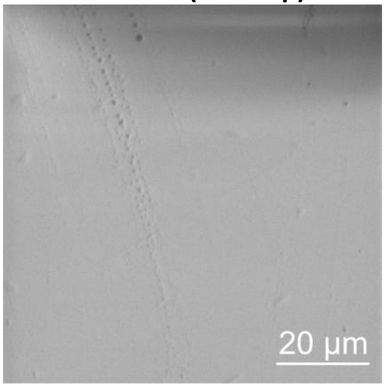

Monte Carlo simulations (CASINO)

d_e		$<d_{PE}>$ (d_{stdv})	
1 keV	30 keV	1 keV	30 keV
13.2 nm	188 nm	12.8 nm (4.8 nm)	2020 nm (744 nm)
$<d_{BSE}>$ (d_{stdv})		β (BSE/SE)	
3.5 nm (2.5 nm)	658 nm (390 nm)	0.298	0.0.354

Material parameters for simulations

ρ (g/cm^3)	Z	Z_{eff}
4.89	Ba: 56 F: 9	23.15

Raman spectrum

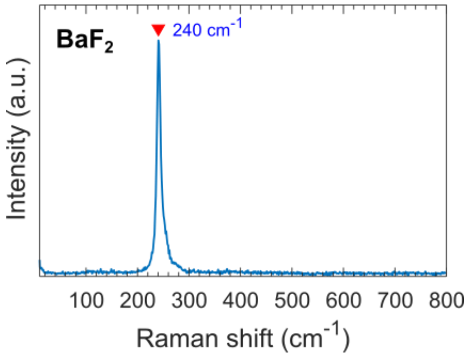

BaF_2 exhibits a characteristic Raman spectrum with a prominent peak at approximately 240 cm^{-1}, corresponding to the T_2g vibrational mode. This mode involves the symmetric stretching of the Ba–F bonds within the crystal lattice [36].

AlN

AlN micro powder was purchased from Thermo Fischer Scientific. The purchased powder was dispersed on sticking carbon tape.

Bright field image

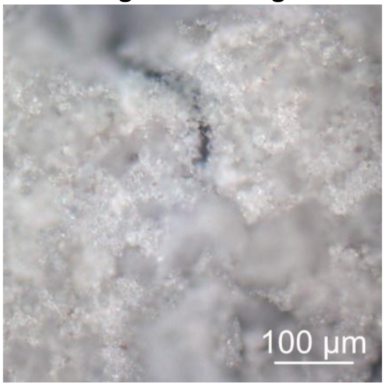

Dark field image

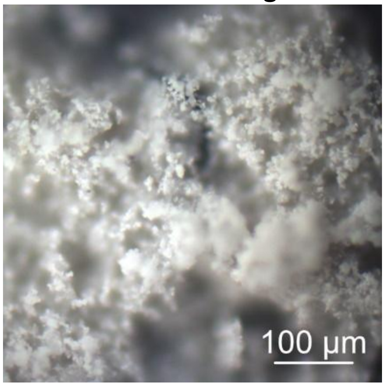

SE 10 keV

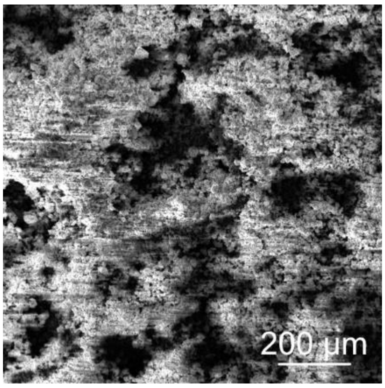

SE 1 keV

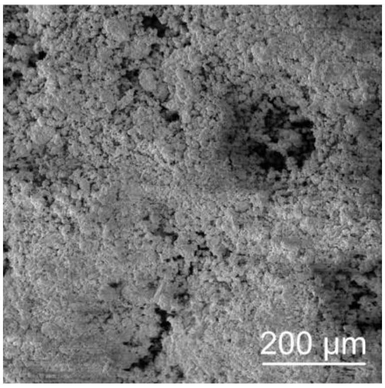

BSE 10 keV

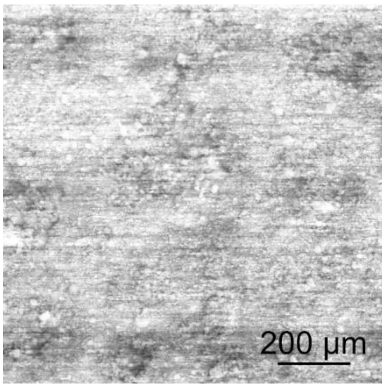

SE 10 keV (close up)

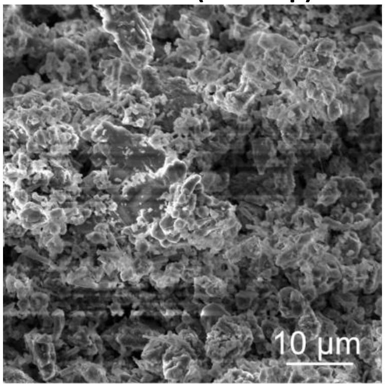

Monte Carlo simulations (CASINO)

d_e		$<d_{PE}>$ (d_{stdv})	
1 keV	30 keV	1 keV	30 keV
13.4 nm	304 nm	11.7 nm (4.0 nm)	3371 nm (1103 nm)
$<d_{BSE}>$ (d_{stdv})		β (BSE/SE)	
3.2 nm (2.1 nm)	1001 nm (568 nm)	0.146	0.099

Material parameters for simulations

ρ (g/cm^3)	Z	Z_{eff}
3.26	Al: 13 N: 7	9.94

Raman spectrum

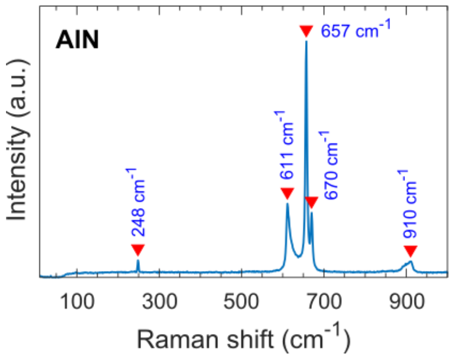

AlN exhibits a characteristic Raman spectrum for the wurtzite structure. The prominent E_2 (high) mode is observed at 657 cm^{-1}, corresponding to non-polar optical phonon vibrations within the hexagonal lattice, while E_2 (low) mode is at 248 cm^{-1}. Additionally, the A_1 (TO) mode appears at 611 cm^{-1}, the E_1 (TO) mode is at 670 cm^{-1}, and the E_1 (LO) mode is around 910 cm^{-1} [37].

[37] L. Shen, T. Cheng, L. Wu, X. Li, and Q. Cui, “Synthesis and optical properties of aluminum nitride nanowires prepared by arc discharge method,” Journal of Alloys and Compounds, vol. 465, no. 1–2. Elsevier BV, pp. 562–566, Oct. 2008. doi: 10.1016/j.jallcom.2007.11.007.

PDMS

PDMS (Polydimethylsiloxane) was purchased in the form of Gel-Film of track level 4 from Gel-Pak. The selected PDMS sheet was fixed on a SEM specimen stub with the help of sticking carbon tape. The optical images show the region of electron beam irradiation during the measurement process.

Bright field image

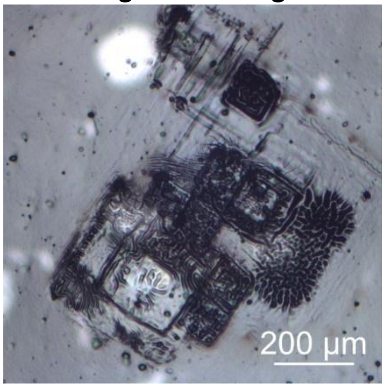

Dark field image

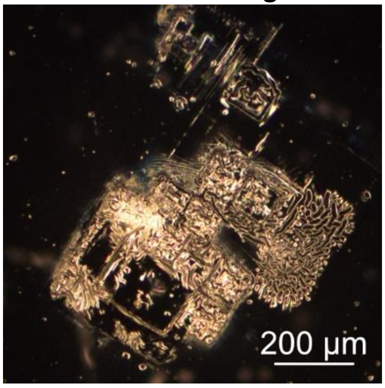

SE 10 keV

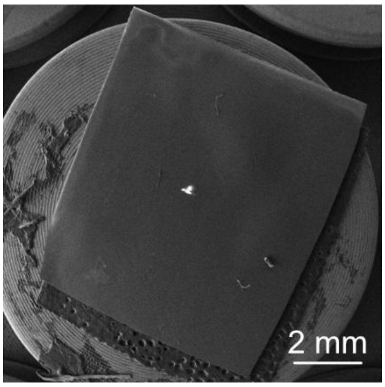

SE 1 keV

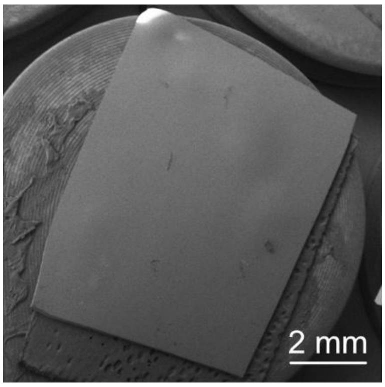

BSE 30 keV

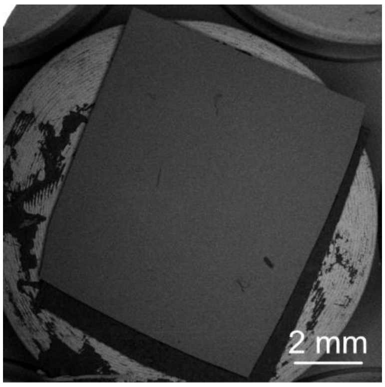

SE 1 keV (close up)

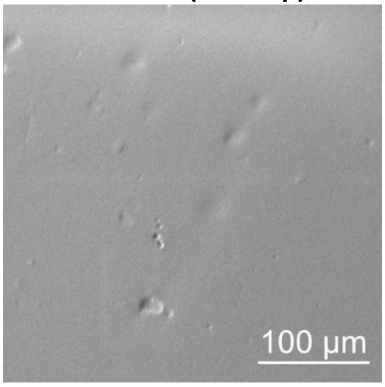

Raman spectrum

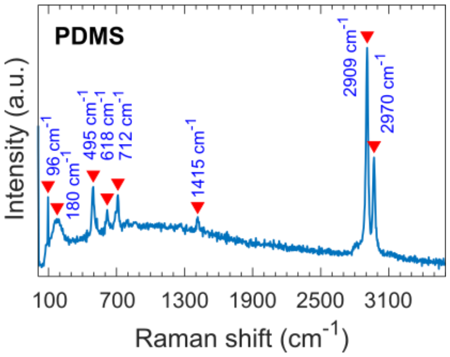

PDMS exhibits a Raman spectrum with multiple peaks as indicated in the attached , which include 96 cm^{-1} and 180 cm^{-1}, which are associated with low-frequency lattice vibrations. The peak at 495 cm^{-1} corresponds to the symmetric stretching of the Si–O–Si bonds, while the 618 cm^{-1} and 712 cm^{-1} peaks are attributed to Si–C stretching and bending vibrations. The peak at 1415 cm^{-1} represents the CH_3 deformation modes, and the high-frequency peaks at 2909 cm^{-1} and 2970 cm^{-1} correspond to C–H symmetric and asymmetric stretching vibrations in the methyl groups [38].

[38] D. Cai, A. Neyer, R. Kuckuk, and H. M. Heise, “Raman, mid-infrared, near-infrared and ultraviolet–visible spectroscopy of PDMS silicone rubber for characterization of polymer optical waveguide materials,” Journal of Molecular Structure, vol. 976, no. 1–3. Elsevier BV, pp. 274–281, Jul. 2010. doi: 10.1016/j.molstruc.2010.03.054.

PC

Polycarbonate (PC) was prepared as 6 wt.% PC solution that is mixed from 10 ml chloroform and 0.89g Poly(Bisphenol A carbonate) (Sigma-Aldrich) and drop casted onto a silicon substrate. The optical images show the region of electron beam irradiation during the measurement process.

Bright field image

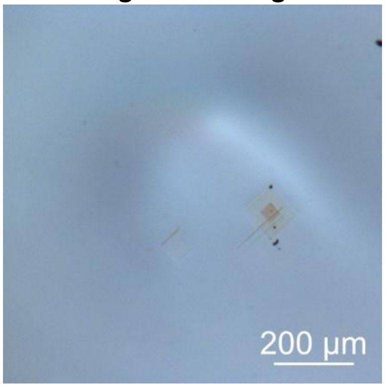

Dark field image

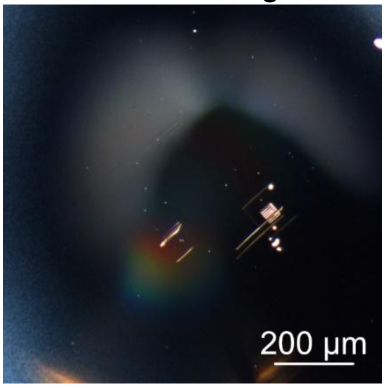

SE 30 keV

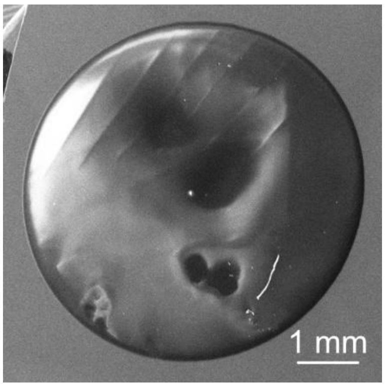

SE 1 keV

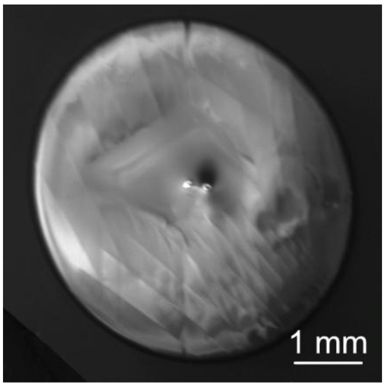

BSE 30 keV

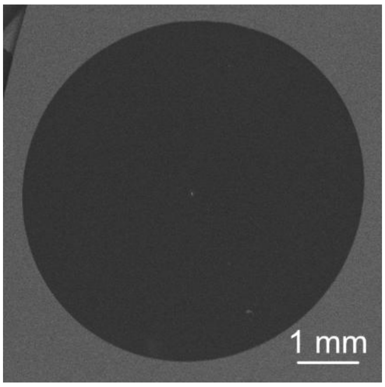

SE 1 keV (close up)

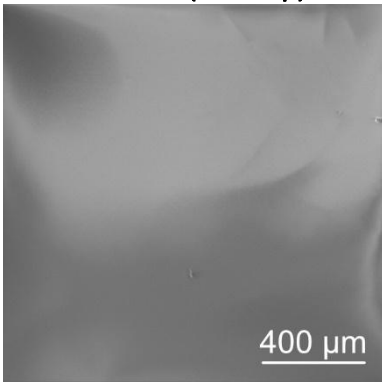

Raman spectrum

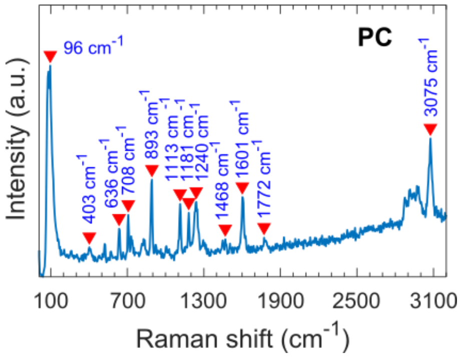

PC exhibits a Raman spectrum with multiple vibrational modes. Prominent peaks are highlighted in the accompanying figure, which are attributed to various stretching and bending vibrations of the polymer's functional groups [39].

[39] M. Petousis et al., “Silicon Carbide Nanoparticles as a Mechanical Boosting Agent in Material Extrusion 3D-Printed Polycarbonate,” Polymers, vol. 14, no. 17. MDPI AG, p. 3492, Aug. 26, 2022. doi: 10.3390/polym14173492.

C-tape

We obtained double-sided adhesive carbon tape from SPI Supplies and mounted it on an SEM stub. We do not provide Monte Carlo calculations due to its unknown chemical composition.

Bright field image

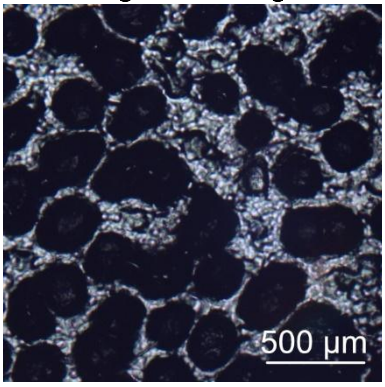

Dark field image

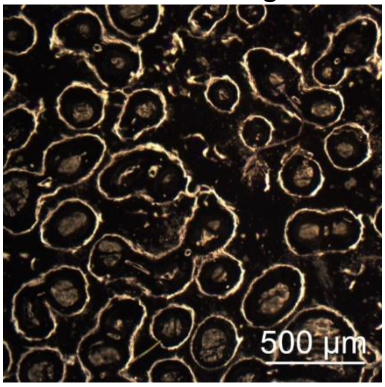

SE 30 keV

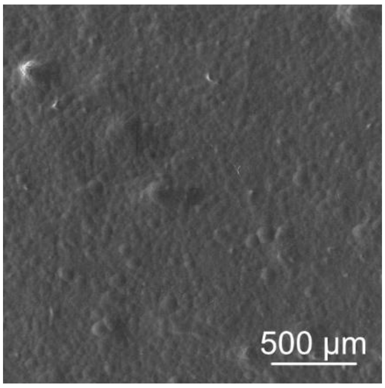

SE 1 keV

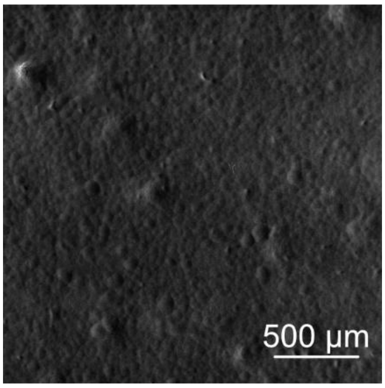

BSE 30 keV

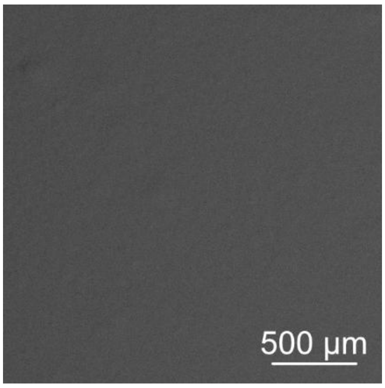

SE 1 keV (close up)

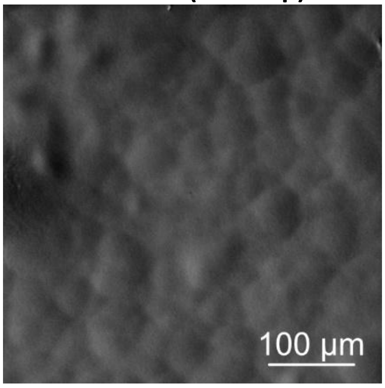

Raman spectrum

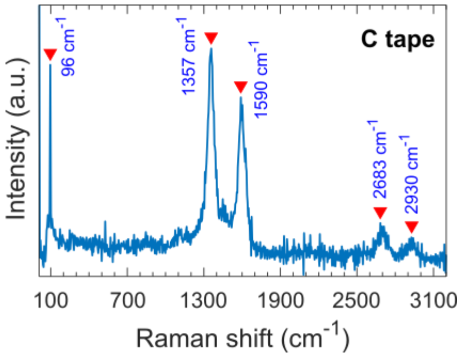

Carbon adhesive tape, commonly used in electron microscopy for sample mounting, exhibits characteristic Raman spectral features indicative of its carbon-based composition. The observed peaks at approximately 1357 cm^{-1} and 1590 cm^{-1} correspond to the D-band and G-band, respectively. The D-band is associated with the presence of defects and disorder in the carbon structure, while the G-band arises from the in-plane vibrational modes of sp^2-bonded carbon atoms. The high frequency peaks around 2683 cm^{-1} and 2930 cm^{-1} are second-order overtones [18].

HSQ

HSQ (hydrogen silsesquioxane) was drop casted on top of a silicon substrate. The silicon substrate with HSQ was fixed on a SEM specimen stub.

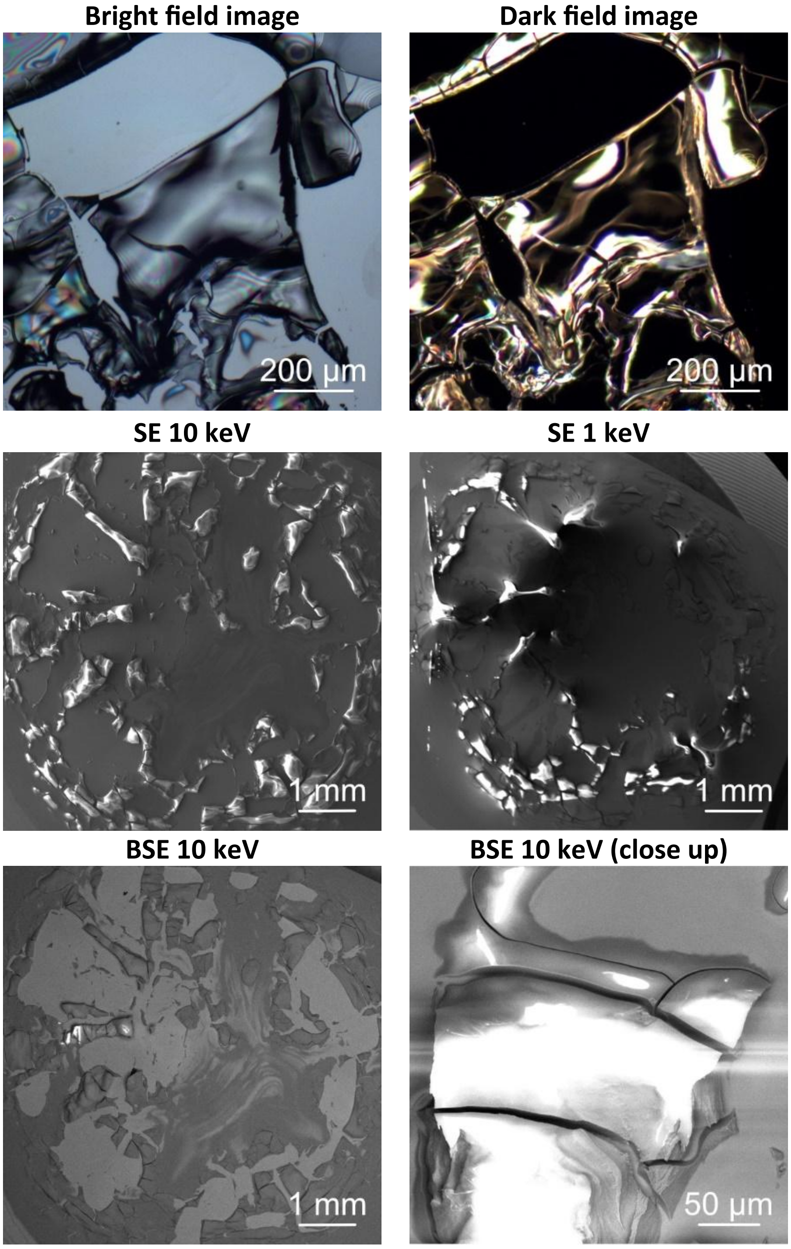

Raman spectrum

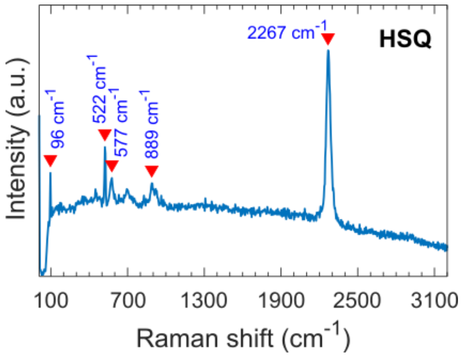

HSQ exhibits a Raman spectrum with multiple vibrational modes. Prominent peaks are highlighted in the accompanying figure, which are attributed to various stretching and bending vibrations of the polymer's functional groups [40].

[40] D. L. Olynick, B. Cord, A. Schipotinin, D. F. Ogletree, and P. J. Schuck, “Electron-beam exposure mechanisms in hydrogen silsesquioxane investigated by vibrational spectroscopy and in situ electron-beam-induced desorption,” Journal of Vacuum Science & Technology B, Nanotechnology and Microelectronics: Materials, Processing, Measurement, and Phenomena, vol. 28, no. 3. American Vacuum Society, pp. 581–587, May 01, 2010. doi: 10.1116/1.3425632.

PMMA

PMMA (Polymethylmethacrylat) was purchased as 950 PMMA A 2 from KAYAKU Advanced-Materials. PMMA was drop casted on top of a silicon substrate, which was fixed on a SEM specimen stub with the help of sticking carbon tape. The optical images show the region of electron beam irradiation during the measurement process.

Bright field image

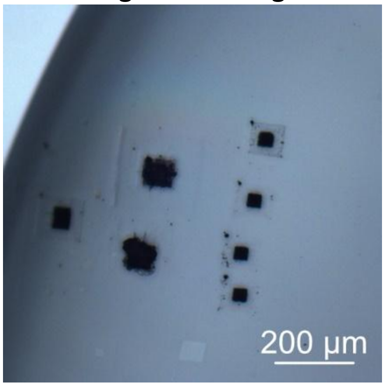

Dark field image

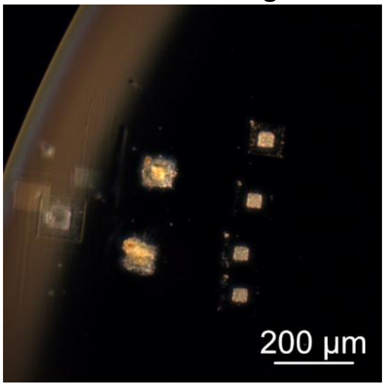

SE 30 keV

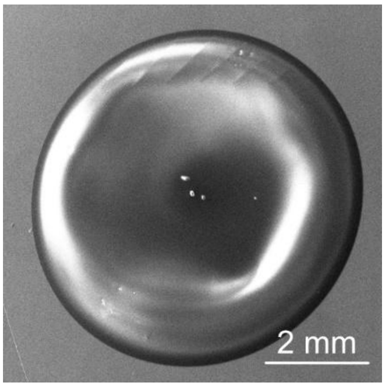

SE 1 keV

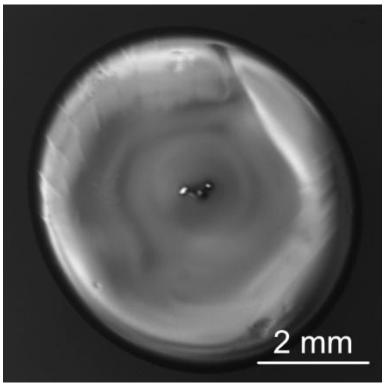

BSE 30 keV

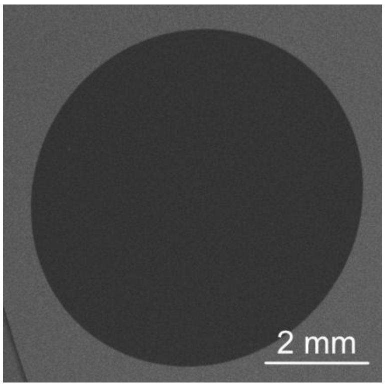

SE 1 keV (close up)

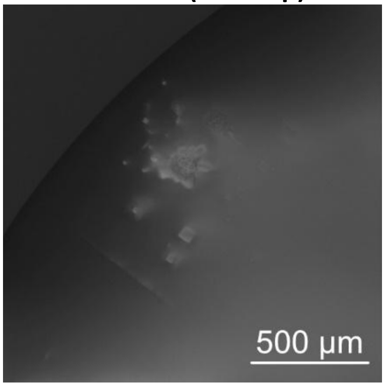

Raman spectrum

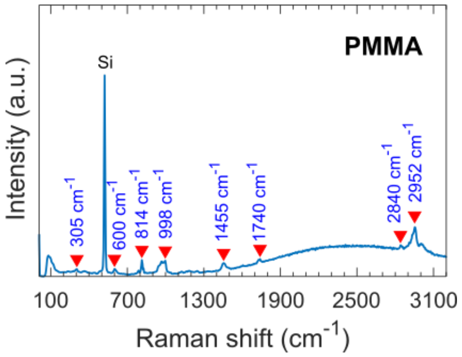

PMMA exhibits multiple prominent peaks as indicated in the corresponding figure, which are attributed to various stretching and bending vibrations of the polymer's functional groups [41].

[41] K. J. Thomas, M. Sheeba, V. P. N. Nampoori, C. P. G. Vallabhan, and P. Radhakrishnan, "Raman spectra of polymethyl methacrylate optical fibres excited by a 532 nm diode pumped solid state laser," Journal of Optics A: Pure and Applied Optics, vol. 10, no. 5. IOP Publishing, p. 055303, 2008. doi: 10.1088/1464-4258/10/5/055303.

SU-8

SU-8 2000 was purchased from KAYAKU Advanced-Materials. SU-8 was drop casted on top of a silicon substrate, which was fixed on a SEM specimen stub with the help of sticking carbon tape.

Bright field image

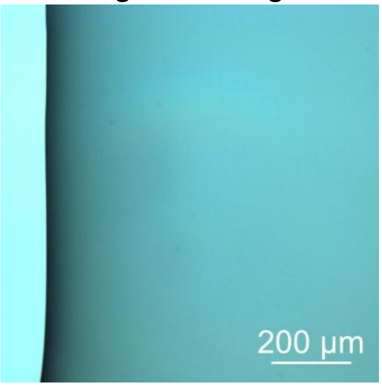

Dark field image

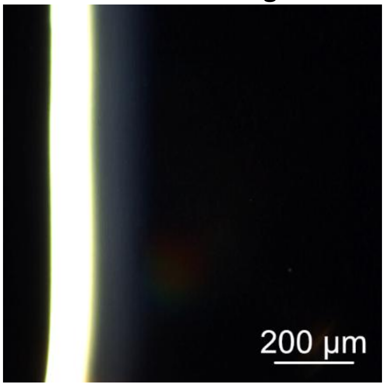

SE 30 keV

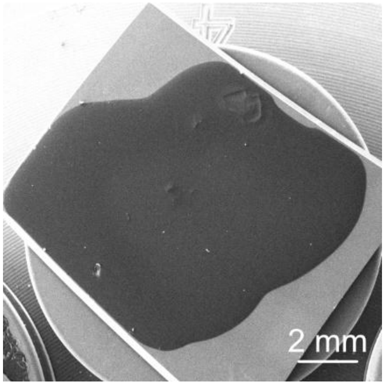

SE 1 keV

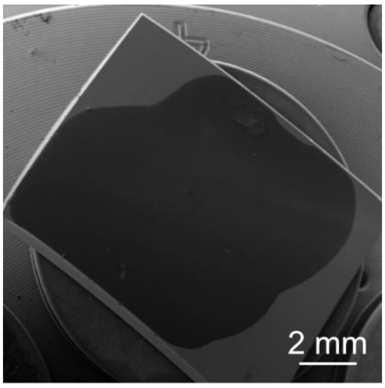

BSE 30 keV

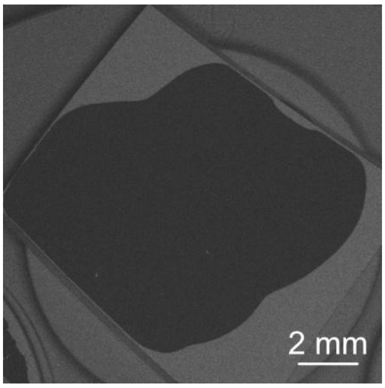

SE 1 keV (close up)

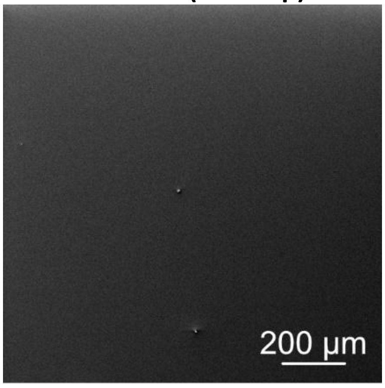

Raman spectrum

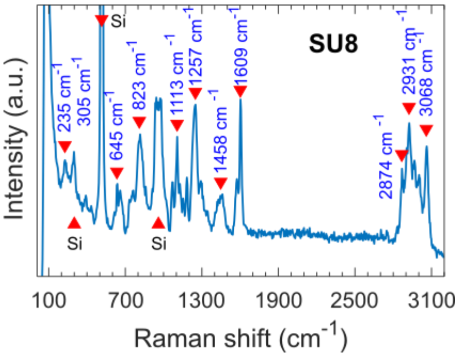

SU8 (bisphenol A) exhibits multiple prominent peaks as indicated in the corresponding figure, which are attributed to the symmetric and asymmetric stretching vibrations of the C–O–C bonds in the epoxy groups. The SU-8 sample was on Si substrate, which characteristic features are indicated in the corresponding figure [42].

[42] K. Chao, W. Schmidt, J. Qin, M. Kim, and F. Tao, “IR and Raman Dual Modality Markers Differentiate among Three bis-Phenols: BPA, BPS, and BPF,” Applied Sciences, vol. 14, no. 14. MDPI AG, p. 6064, Jul. 11, 2024. doi: 10.3390/app14146064.
